# Identifying dynamical modules from genetic regulatory systems: applications to the segment polarity network

**DOI:** 10.1186/1471-2105-8-413

**Published:** 2007-10-25

**Authors:** David J Irons, Nicholas AM Monk

**Affiliations:** 1 Department of Computer Science, University of Sheffield, UK; 2 School of Mathematical Sciences, University of Nottingham, UK

## Abstract

**Background:**

It is widely accepted that genetic regulatory systems are 'modular', in that the whole system is made up of smaller 'subsystems' corresponding to specific biological functions. Most attempts to identify modules in genetic regulatory systems have relied on the topology of the underlying network. However, it is the temporal activity (dynamics) of genes and proteins that corresponds to biological functions, and hence it is dynamics that we focus on here for identifying subsystems.

**Results:**

Using Boolean network models as an exemplar, we present a new technique to identify subsystems, based on their dynamical properties. The main part of the method depends only on the stable dynamics (attractors) of the system, thus requiring no prior knowledge of the underlying network. However, knowledge of the logical relationships between the network components can be used to describe how each subsystem is regulated. To demonstrate its applicability to genetic regulatory systems, we apply the method to a model of the *Drosophila *segment polarity network, providing a detailed breakdown of the system.

**Conclusion:**

We have designed a technique for decomposing any set of discrete-state, discrete-time attractors into subsystems. Having a suitable mathematical model also allows us to describe how each subsystem is regulated and how robust each subsystem is against perturbations. However, since the subsystems are found directly from the attractors, a mathematical model or underlying network topology is not necessarily required to identify them, potentially allowing the method to be applied directly to experimental expression data.

## Background

Genetic regulatory systems are assumed to be 'modular', with specific combinations of genes and proteins responsible for different biological functions. Although there is no authoritative definition of a 'module', one common description is a group of genes, proteins and/or molecules that combine to carry out a relatively distinct function (distinguishable from the functions associated with other modules) [1]. Rather than being a protein complex or group of co-expressed genes, such a module can be viewed as the temporal activity (dynamics) of a group of genes/proteins that controls a specific function in different environmental conditions, cell types and/or tissues. For example, the temporal activity of genes/proteins controlling progression through the cell cycle (in many different environmental conditions and cell types) can be viewed as a module. Moreover, since genetic regulatory systems are hierarchical [2], it is likely that these modules also interact in a hierarchical manner.

It is this concept of a 'dynamical module' or 'subsystem' that we consider in this paper. An important challenge in biology is how to identify such subsystems and discover how they combine hierarchically to determine cell types, tissues and organisms. A dynamic approach is the most suitable starting point for decomposing a genetic regulatory system into modules, since it is the activity profiles that control biological functions. The topology of the underlying interaction network is just a description of the interactions associated with the activity profiles.

**Figure 1:**
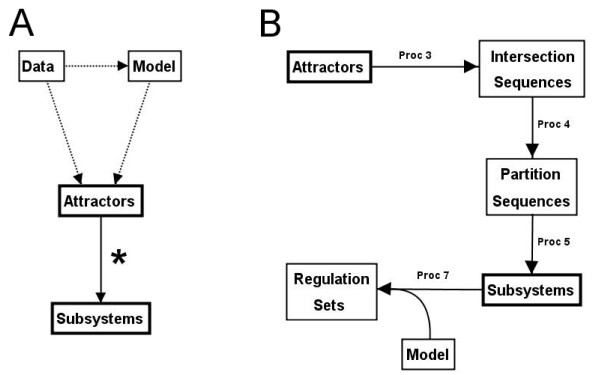
**Overview of methods**. (A) The main method of identifying subsystems only requires a set of attractors (*). These attractors can be found either from a model or directly from experimental data, meaning that a known network topology is not essential. (B) More detailed schematic of the methods, showing the procedures required at each step (given in the *Methods *section). The attractors are used to find intersection sequences and partition sequences (Stage 1), which are then used to identify subsystems (stage 2). Once the subsystems are identified, regulation sets can be found with the help of a suitable mathematical model.

Most previous attempts at modular decomposition have relied on the topology of the underlying network, deduced from protein-protein interaction and/or transcription factor binding data. From a generic network point of view, algorithms have been created which partition any network into topological modules [3]. From a biological viewpoint, transcription factor networks have been used to find 'network motifs' [4] and 'structural modules' [5], based solely on topological properties of the network. Although informative, topology based approaches do not necessarily allow the functions/dynamics associated with groups of genes/proteins to be inferred [6]; rather, activity/expression levels over a period of time are required. When expression data have been used, the resulting modules tend to be groups of genes whose expression levels change in unison. For example, 'transcription factor modules' [7,8] are groups of genes with common transcription factor binding sites and expression patterns. However, for a biological function, it may be the case that a series of interactions are involved, with different genes being affected at different points in time (e.g. genes involved in cell cycle regulation). Similar issues arise when applying clustering algorithms to expression data, which also groups together genes whose expression levels change in unison. In this paper, we present a method for identifying subsystems ('dynamical modules'), given a set of discrete state, discrete time attractors. The subsystems are found directly from the attractors (Fig. 1), without the need for topological information, making the method applicable both to models and (potentially) to experimental expression data. To demonstrate the method, we consider a class of mathematical models called Boolean network models (defined in the *Methods *section). These models are used as a starting point because of their relative simplicity and the fact that different dynamics can be easily compared. We also consider how each subsystem is regulated and how subsystems can help investigate robustness in these systems. In order to test our method, we then apply it to a model of the *Drosophila *segment polarity network.

## Results: Identifying subsystems and regulatory interactions between subsystems

Given a set of discrete-state, discrete-time attractors as an input, we have produced a method that optimally breaks the attractors up and identifies *subsystems*, each one

1. Conserved across some set of attractors,

2. Distinguishable from the stable dynamics for the rest of the system.

**Figure 2:**
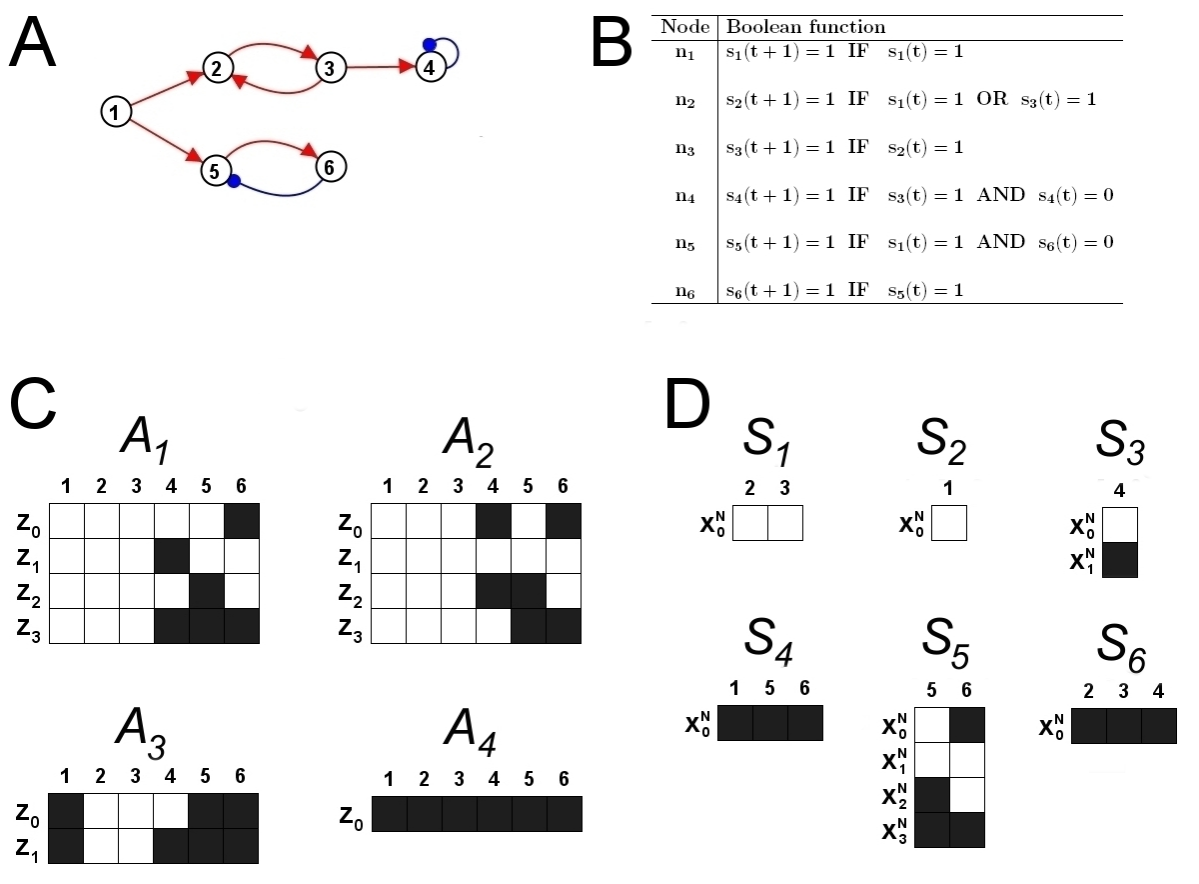
**Subsystems in the example Boolean network model**. (A, B) Network and Boolean functions for a simple Boolean network model. If the logical condition in a Boolean function is satisfied (for node *n*_*i*_), then that node takes state 1 at the following time step (*s*_*i*_(*t *+ 1) = 1). Otherwise, if the logical condition fails, *s*_*i*_(*t *+ 1) = 0. Interactions in the model are summarised by the network edges in A (red arrow = activation, blue dot = inhibition). (C) *A*_1_, ..., *A*_4 _are the 4 possible attractors for the Boolean network model in A, B, which are consistent with both synchronous and asynchronous updating schemes. Each column corresponds to a node in the model, whilst each row corresponds to an attractor state. White/Black corresponds to the node having state 1/0 in the attractor state. Once the system enters an attractor it continually cycles through those attractor states (e.g. **z**_0_, **z**_1_, **z**_2_, **z**_3_, **z**_0_, **z**_1_, **z**_2_, **z**_3_, **z**_0_, **z**_1 _after the system enters *A*_1_). (D) 6 Subsystems identified for this model, corresponding to sub-dynamics that are conserved across/distinguish sets of attractors from C. These were identified by applying the new method from this paper to the 4 attractors *A*_1_, ..., *A*_4_. Each column corresponds to a node in the model, whilst each row corresponds to a partial state. White/Black corresponds to the node having state 1/0 in the partial state.

In order to demonstrate the key features of the resulting subsystems, we use the simple example network in Fig. 2. Here, *A*_1_, ..., *A*_4 _are four attractors for the system, but it is evident that the sub-dynamics associated with some sub-networks (*S*_1_, ..., *S*_6_) can distinguish different sets of attractors and highlight parts of the system that are dynamically/functionally distinct. For example, the subsystem *S*_1 _is conserved across three attractors (*A*_1_, *A*_2 _and *A*_3_) and provides a way of distinguishing those attractors from the remaining attractors (*A*_4_). Moreover, *S*_1 _can be viewed as dynamically distinct from all remaining sub-dynamics, since the dynamics of each remaining node (1, 4, 5 and 6) has a distinguishable profile when viewed alongside *S*_1_. In particular,

**(a) **Nodes 1, 5 and 6 each exhibit different behaviours/dynamics alongside *S*_1 _(compare *A*_3 _with *A*_1 _and *A*_2_).

**(b) **The activity of node 4 oscillates out of phase with the activity of nodes 5 and 6, when viewed alongside *S*_1 _in attractors *A*_1 _and *A*_2_.

The sub-dynamics *S*_1_, ..., *S*_6 _in Fig. 2 are all subsystems (according to our method) because each one can be viewed as dynamically distinct from all the other sub-dynamics in attractors *A*_1 _– *A*_4_. The attractors themselves than can be viewed as combinations of subsystems.

The main method does not explicitly identify functional/regulatory relationships between subsystems. However, in the case of logical models such as Boolean network models, the logical functions can be used to identify suitable regulatory relationships.

For the remainder of this section we describe these new techniques in terms of Boolean network models (which we formally define in the *Methods *section). Algorithms for these new techniques are described in the *Methods *section of this paper, whilst formal proofs for all the methods can be found in Additional files 1 and 2. It should be noted that all of the definitions and algorithms can be extended to any set of discrete state, discrete time attractors, with or without a detailed mathematical model.

### Partial state sequences

Much of this paper involves looking at the sub-dynamics in Boolean network models. Therefore, for a subset of nodes *N *⊆ *V*, we consider *partial states*.

**Definition 1. **A *partial state*, **x**^*N *^∈ {0, 1}^|*N*| ^is a set of Boolean states, one for each node *n*_*i *_∈ *N *⊆ *V*. i.e. **x**^*N *^= {*s*_*i*_: *n*_*i *_∈ *N*}.

**Definition 2. **A *partial state sequence*, $P=\{x_{0}^{N},x_{1}^{N},...,x_{q-1}^{N}\}$, is an ordered set of partial states, for a node set *N *(⊆ *V*).

These partial state sequences can be used to represent sub-dynamics in Boolean network models, and therefore, can be used as a starting point when defining subsystems. In particular, we are looking for partial state sequences that occur (or cycle within) attractors.

**Definition 3. **A partial state sequence $P=\{x_{0}^{N},x_{1}^{N},...,x_{q-1}^{N}\}$*occurs *in an attractor *A *= {**z**_0_, ..., **z**_*p*-1_} if there exists integers *b*_0_, ..., *b*_*p*-1 _∈ {0, ..., *q *- 1} for which the following are true

1. For *k *= 0, ..., *p *- 1, $x_{b_{k}}^{N}$ = {*s*_*i *_∈ **z**_*k *_: *n*_*i *_∈ *N*}.

2. For each *k *∈ {0,..., *p *- 1} and *j *= *k *- 1 (mod *p*), either

**(a) ***b*_*k *_= *b*_*j *_or **(b) ***b*_*k *_= *b*_*j *_+ 1 (mod *q*)

3. Properties 1 and 2 are not true for any smaller partial state sequence $P^{\prime }=\{y_{0}^{N},y_{1}^{N},...,y_{q^{\prime }-1}^{N}\}$ and integers *c*_0_, ..., *c*_*p*-1 _∈ {0, ..., *q' *- 1} (*q' *<*q*).

As an example of a partial state sequence that occurs in an attractor, consider the partial state sequence

$$P=\{\begin{array}{ll} x_{0}^{N}=\{s_{3}=1,s_{5}=1\} & (N=\{n_{3},n_{5}\}),\\ x_{1}^{N}=\{s_{3}=1,s_{5}=0\} & \left(N=\{n_{3},n_{5}\}\right) \end{array}$$

and the attractor *A*_1 _= {**z**_0_, **z**_1_, **z**_2_, **z**_3_} in Fig. 2. As the system enters the attractor *A*_1_, we continually cycle through the component states over time (i.e. **z**_0_, **z**_1_, **z**_2_, **z**_3_, **z**_0_, **z**_1_, **z**_2_, **z**_3_, **z**_0_, ...). Therefore, since

- $x_{0}^{N}$ is contained in **z**_0 _and **z**_1 _(i.e. $x_{0}^{N}$ = {*s*_*i *_∈ **z**_0 _: *n*_*i *_∈ *N*} = {*s*_*i *_∈ **z**_1 _: *n*_*i *_∈ *N*})

- $x_{1}^{N}$ is contained in **z**_2 _and **z**_3 _(i.e. $x_{1}^{N}$ = {*s*_*i *_∈ **z**_2 _: *n*_*i *_∈ *N*} = {*s*_*i *_∈ **z**_3 _: *n*_*i *_∈ *N*})

we also continually cycle through the partial states of *P *over time (i.e. $x_{0}^{N},x_{1}^{N},x_{0}^{N},x_{1}^{N},x_{0}^{N}$, ...). We note that the time taken to change from one partial state to the next is not considered (2 time steps in this example). Ignoring such time lags could be especially important in genetic regulatory systems, where the same process may take different lengths of time under different conditions (in different attractors). For example, in cell cycle regulation, some mutations can alter the time taken for a round of cell division, without necessarily altering the relationships that exist between other groups of genes/proteins [9]. Properties 1 and 2 allow us to capture the fact that *P *continually cycles within the the attractor *A*_1 _by noting that a sequence of integers {*b*_0_, *b*_1_, *b*_2_, *b*_3_} = {0, 0, 1, 1} exists that maps the partial states in *P *onto the attractor states in *A*_1_. Property 2 ensures that the partial states in *P *occur in the correct order, whilst allowing different lengths of time between each change. Properties 2 and 3 ensure that *P *is the smallest set of partial states that occurs in the attractor *A*. This leaves a partial state sequence that just describes the 'order' in which the node states change in *A *(for nodes in *N*), ignoring any time lags associated with individual partial states and the number of times *P *cycles within *A*. Returning to the example in Fig. 2, *S*_3 _cycles twice in *A*_1 _and *A*_2_. However, just two partial states ($x_{0}^{N}$ = {*s*_4 _= 1} and $x_{1}^{N}$ = {*s*_4 _= 0}) are sufficient to capture the fact that *S*_3 _occurs in *A*_1 _and *A*_2 _(letting {*b*_0_, *b*_1_, *b*_2_, *b*_3_} = {0, 1, 0, 1} and {1, 0, 1, 0} respectively).

Given a node set *N *and a set of attractors, the *Methods *section of this paper gives detailed algorithms of how to identify the unique partial state sequences (within an order of rotation) that occur in each attractor. Essentially this is done by going through each attractor state and writing down the partial states involving the node set *N*. We then remove partial states when

**(a) **Multiple adjacent partial states are identical (in which case only one copy is kept)

**(b) **A sequence of states cycles many times within an attractor (in which case only one copy is kept).

leaving a partial state sequence that just describes the 'order' in which the node states change in that attractor.

### Method for identifying subsystems

**Figure 3:**
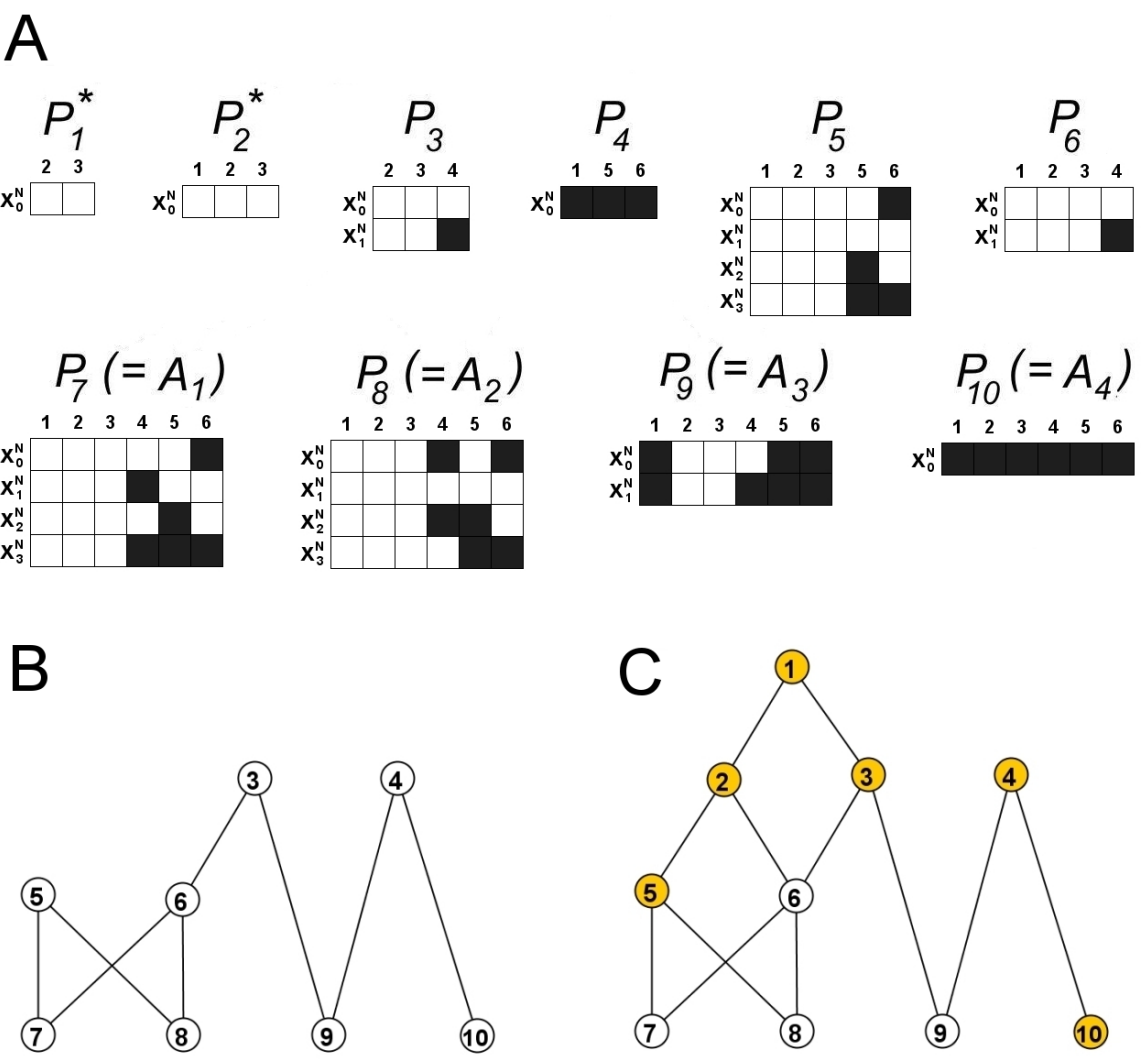
**Partition sequences in the example Boolean network model**. (A) 10 partition sequences are identified for the example model in Fig. 2. Each column in each sequence corresponds to a node in the model, whilst each row corresponds to a partial state. White/Black corresponds to the node having state 1/0 in the partial state. Eight of these (*P*_3 _– *P*_10_) are also intersection sequences, whilst the remaining two (*P*_1 _and *P*_2_, starred) are core sequences that underlie multiple intersection sequences (see Tables 1 and 2). (B, C) Examples of hierarchy amongst the sequences in A. In each case, node *i *corresponds to the partial state sequence *P*_*i*_. In each case, if a link joins a partial state sequence *P*_*x *_(top) to another *P*_*y *_(bottom), *P*_*x *_occurs in *P*_*y *_and is conserved across a greater number of attractors. (B) Hierarchy between intersection sequences. (C) Hierarchy between partition sequences. Orange nodes correspond to sequences with sub-dynamics that are distinct from those in sequences further up the hierarchy. These 6 distinct sub-dynamics are the subsystems (see Table 3). White nodes correspond to sequences that are just a combination of sequences further up the hierarchy.

The new method of identifying subsystems is a two stage process that breaks up the system's attractors, to leave partial state sequences that are optimally distinguishable from one another. A summary of these two stages, along with relevant definitions, is given below using the simple example in Fig. 2 and Fig. 3. Detailed descriptions of the algorithms are given in the *Methods *section for those wanting to implement this method. A flow diagram of the procedures involved can be seen in Fig. 1

#### Stage 1: Partition sequences

The first stage of the method involves identifying *every *partial state sequence that satisfies Definition 5. Each such *partition sequence *(for a node set *N*) is both (a) conserved across a set of attractors and (b) has a fundamentally different dynamical profile from that associated with any larger node set *M *⊇ *N*. We first introduce *intersection sequences *that are used in the definition of partition sequences and set up a hierarchy amongst them.

**Definition 4. **A partial state sequence *P *for a node set *N *is an *intersection sequence*, if there exists a subset of attractors ℂ(⊆A) for which the following hold

1. *P *occurs in every attractor *A *∈ ℂ

2. *P *does not occur in any attractor *A *∉ ℂ

3. Given a larger node set *M *⊃ *N*, there is no partial state sequence *P' *(for the node set *M*) that satisfies condition 1.

If the above properties hold, we say *P intersects *at ℂ.

In order to identify every intersection sequence, node sets (*N*) are analysed to identify which partial state sequences occur in which attractors. Any such partial state sequence (*P*) and corresponding set of attractors (ℂ) will then satisfy properties 1 and 2 of Definition 4. These pairs {*P*, ℂ} are stored for each node set *N *and then compared during the procedure to remove those that fail property 3. In the *Methods *section of this paper we give a detailed algorithm for this as well as providing some notes on improving efficiency. In practice, many node sets *N *and/or attractors can be ignored because we know that the pairs {*P*, ℂ} will fail property 3.

The set of all intersection sequences provide a hierarchical breakdown of the system's dynamics (see Fig. 3A, B and Table 1). At the top of the hierarchy are partial state sequences that contain only a few nodes but are conserved over (relatively) many attractors. Then as you go down the hierarchy, extra nodes are added but the resulting partial state sequences are conserved over fewer attractors. Property 3 above ensures that each partial state sequence is optimal in the sense that no more extra nodes can be considered without the new extended partial state sequence occurring in strictly fewer attractors.

**Table 1:** Intersection sequences in the example Boolean network model

Intersection sequences	Intersects at ...
*P*_3_	ℂ_3 _= {*A*_1_, *A*_2_, *A*_3_}
*P*_4_	ℂ_4 _= {*A*_3_, *A*_4_}
*P*_5_	ℂ_5 _= {*A*_1_, *A*_2_}
*P*_6_	ℂ_6 _= {*A*_1_, *A*_2_}
*P*_7_	ℂ_7 _= {*A*_1_}
*P*_8_	ℂ_8 _= {*A*_2_}
*P*_9_	ℂ_9 _= {*A*_3_}
*P*_10_	ℂ_10 _= {*A*_4_}

The following table gives all the intersection sequences for the simple example model (in Fig. 2 and Fig. 3). The table shows which partial state sequences (from Fig. 3) are intersection sequences, along with the attractors they occur in. The attractors (*A*_1 _- *A*_4_) can be seen in Fig. 2.

Although these sequences provide a neat hierarchical breakdown of the system's dynamics, some may be superfluous whilst important 'core' sub-dynamics may be missed. Therefore, we introduce 3 extra constraints when defining partition sequences.

**Definition 5. **A partial state sequence $P=\{x_{0}^{N},x_{1}^{N},...,x_{q-1}^{N}\}$ is a *partition sequence *if it satisfies any of the following properties, for some set of attractor cycles ℂ(⊆A)

**A : ***P ***is Core to **ℂ

The following 3 properties hold for *P*

1.*P *occurs in an intersection sequence *P'*, which intersects at ℂ (*P *can equal *P'*).

2. If an intersection sequence *Q *(for a node set *M*) intersects at D (where D∩ℂ≠∅), then there exists an intersection sequence *Q' *(for a node set *M' *⊇ *M *∪ *N*) that occurs in every attractor *A *∈ D∩ℂ

3. 1 and 2 are not true for any larger partial state sequence *P" *(for a node set *N" *⊃ *N*)

**B : ***P ***is Exclusive to **ℂ

*P *is the only intersection sequence that intersects at ℂ.

**C : ***P ***is Independently Oscillating**

*P *intersects at ℂ and cycles out of phase with another intersection sequence *Q*. i.e. ∃ *Q *that involves the node set *M *and intersects at D, for which

1. |ℂ∩D| ≥ 2

2. *N *∪ *M *= *V *(the set of all nodes)

The three parts of this definition are used to describe three ways in which a sub-dynamic (partial state sequence) can be viewed as fundamental to the make up of the attractors and/or distinguishable from the remaining sub-dynamics. For the example in Fig. 2 and Fig. 3, Table 2 shows the partial state sequences that satisfy properties A, B and C above.

**Table 2:** Partition sequences in the example Boolean network model

Partition sequences	Core to ...	Exclusive to ...	Independently Oscillating with ...
*P*_1_	{*A*_1_, *A*_2_, *A*_3_}	×	×
*P*_2_	{*A*_1_, *A*_2_}	×	×
*P*_3_	×	{*A*_1_, *A*_2_, *A*_3_}	*P*_5_
*P*_4_	{*A*_3_, *A*_4_}	{*A*_3_, *A*_4_}	×
*P*_5_	×	×	*P*_3 _and *P*_6_
*P*_6_	×	×	*P*_5_
*P*_7_	{*A*_1_}	{*A*_1_}	×
*P*_8_	{*A*_2_}	{*A*_2_}	×
*P*_9_	{*A*_3_}	{*A*_3_}	×
*P*_10_	{*A*_4_}	{*A*_4_}	×

The following table gives all the partition sequences for the simple example model (in Fig. 2 and Fig. 3). The partition sequences (*P*_1 _– *P*_10_) can be seen in Fig. 3, whilst the attractors (*A*_1 _– *A*_4_) can be seen in Fig. 2. The table also shows which properties they satisfy in Definition 5 (× implies that property is not satisfied)

Two partition sequences *P*_1 _and *P*_2 _are not intersection sequences, but are core to the dynamics associated with different sets of attractors. For example, *P*_2 _is the core dynamic spanning attractors *A*_1 _and *A*_2_, rather than *P*_5 _and *P*_6_, which both occur in *A*_1 _and *A*_2_. This is because *P*_5 _and *P*_6 _involve partially overlapping node sets *N*_5 _and *N*_6 _(i.e. *N*_5 _⊈ *N*_6_, *N*_6 _⊈ *N*_5_), and so neither corresponds to a sub-network whose dynamics are core to attractors *A*_1 _and *A*_2_. On the other hand *P*_2 _acts as a central point from which these intersections sequences branch off.

Although in this example every intersection sequence is also a partition sequences, this does not necessarily have to be the case. One example where this is not the case is given in Section S3.2 of Additional file 3. In order to identify every partition sequence, we use the full set of intersection sequences to identify every partial state sequence satisfying property **A**, **B **or **C **of Definition 5. These 3 tests are done independently and the partial state sequences found in each test are grouped together to give the full set of partition sequences. In the *Methods *section of this paper we give detailed algorithms for these procedures.

#### Stage 2: Subsystems

The second stage of the method compares the partition sequences, to identify *every *partial state sequence that satisfies Definition 6. The partition sequences allow the attractors to be broken up in a hierarchical manner. However, these sequences are not subsystems (for a start, all of the attractors themselves are partition sequences). Of more interest are the key sub-dynamics that are unique to a particular partition sequence and that distinguish one level in the hierarchy from the next. Therefore, we define subsystems to be those components that are unique to a partition sequence. i.e.

**Definition 6. **A partial state sequence *S *(for a node set *N*) is a *subsystem *if it is unique to a partition sequence. i.e. there exists a partition sequence *P *(for a node set *M*) for which

1. *S *occurs in *P*.

2. If another partition sequence *P' *(for a node set *M' *⊂ *M*) occurs in *P*, then *M' *∩ *N *= ∅.

3. 1 and 2 are not true for any partial state sequence *S'*, for a larger node set *N' *⊃ *N*.

In the *Methods *section of this paper we give detailed algorithm for identifying every such subsystem. This essentially involves running through every partition sequence *P *(for a node set *M*) and identifying the nodes (*N*) that do not belong to any other partition sequence *P'*, that occurs in *P*. The dynamics associated with this node set *N *are then a subsystem.

As an example, consider *P*_5 _in Fig. 3. Looking at the remaining partition sequences we see that only *P*_1 _and *P*_2 _occur within it. Therefore, the nodes and dynamics in *P*_5 _that are unique to *P*_5 _are the node set *N *= {*n*_5_, *n*_6_} and the subsystem *S*_5 _(from Fig. 2D). Obviously, *S*_5 _satisfies Property 1 of Definition 6 because it occurs in the partition sequence *P*_5_. Property 2 is satisfied since *P*_1 _and *P*_2 _are the only partition sequences occurring in *P*_5 _and the node sets do not intersect with *N *= {*n*_5_, *n*_6_} ({*n*_2_, *n*_3_} ∩ {*n*_5_, *n*_6_} = ∅ and {*n*_1_, *n*_2_, *n*_3_} ∩ {*n*_5_, *n*_6_} = ∅). Property 3 is satisfied since any node set *N' *⊆ *M*, *N' *⊃ *N *would fail Property 2. For the example Boolean network model, all the subsystems can be seen in Fig. 2, whilst Fig. 3C and Table 3 demonstrate which subsystem corresponds to which partition sequences.

**Table 3:** Unique components of partition sequences in the example Boolean network model

Partition sequence	Unique Component
*P*_1_	*S*_1_
*P*_2_	*S*_2_
*P*_3_	*S*_3_
*P*_4_	*S*_4_
*P*_5_	*S*_5_
*P*_6_	N/a
*P*_7_	N/a
*P*_8_	N/a
*P*_9_	N/a
*P*_10_	*S*_6_

For the partition sequences in Table 2 and Fig. 3, the following table gives components that are unique to each one. These are the subsystems shown in Fig. 2.

### Regulation of subsystems

We say a collection of subsystems S_*x *_= {*S*_1_, ..., *S*_*f*_} *triggers *an individual subsystem *S*_*y *_in an attractor *A*, if the co-occurrence of subsystems *S*_1_, ..., *S*_*f *_ensures the occurrence of *S*_*y *_in *A*.

For Boolean network models (or other discrete-state models), such interactions can be identified by considering the Boolean functions (or Logical functions). In the case of the simple example in Fig. 2, it is possible to look at the Boolean functions and say that

**(a) ***S*_2 _can *trigger *the occurrence of *S*_1 _in attractors *A*_1 _and *A*_2_.

**(b) ***S*_1 _can *trigger *the occurrence of itself (*S*_1_) in attractors *A*_1_, *A*_2 _and *A*_3_.

This is since

**(a) **The prolonged activation of node 1 (*s*_1 _= 1) is sufficient to activate nodes 2 and 3 (*s*_2 _= 1, *s*_3 _= 1).

**(b) **If nodes 2 and 3 are both on at time *t*, then this sufficient to maintain their activation for all time steps *t' *≥ *t*.

As can be seen above, different collections may act in different sets of attractors to fully explain the occurrence of a subsystem *S*_*y*_.

**Definition 7. **A set of subsystem collections $S_{1},...,S_{g}$*regulates *an (individual) subsystem *S*_*y *_if the following are true

1. For *i *= 1, ..., *g*, ∃ an attractor *A *for which S_*i *_triggers *S*_*y *_in *A*.

2. If *S*_*y *_occurs in an attractor *A*, ∃ *i *∈ {1, .., *g*} for which S_*i *_triggers *S*_*y *_in *A*.

We call the set {$S_{1},...,S_{g}$} the *regulation set *of *S*_*y*_.

In the *Methods *section, we describes how a suitable regulation set can be found for each subsystem *S*_*y*_, by looking at how each partial state within *S*_*y *_is triggered/regulated in each attractor. This approach uses the Boolean functions from a Boolean network model to identify which partial states can trigger the occurrence of those in *S*_*y*_.

For the simple example in Fig. 2, Table 4 shows a regulation set for each of the subsystems. As can be seen in this example it is often the case that subsystems play a role in their own regulation, although this does not necessarily have to be the case.

**Table 4:** Regulation of subsystems in the example Boolean network model

Subsystem	Regulation set
*S*_1_	S = {*S*_1_}
	S = {*S*_2_}
*S*_2_	S = {*S*_2_}
*S*_3_	S = {*S*_1_, *S*_3_}
	S = {*S*_2_, *S*_3_}
*S*_4_	S = {*S*_4_}
*S*_5_	S = {*S*_2_, *S*_5_}
*S*_6_	S = {*S*_4_, *S*_6_}

Table showing the collections of subsystems that regulate each individual subsystem *S*_*y *_(for the example Boolean network model and subsystems in Fig. 2). Column 2: Each row shows a collection of subsystems (S_*group*_) that can trigger *S*_*y*_.

Even without the Boolean functions, it is possible to identify relationships between subsystems. On a simple observational level, a subsystem *S*_*x *_may be *hierarchically linked *to another subsystem *S*_*y*_, because *S*_*x *_only occurs in an attractor in conjunction with the 'higher order' *S*_*y*_. i.e.

**Definition 8. **Consider two subsystems *S*_*x *_and *S*_*y*_. *S*_*x *_is *hierarchically linked *to *S*_*y *_if the following are true

- *S*_*x *_occurs in an attractor *A *⇒ *S*_*y *_occurs in an attractor *A*

Furthermore, such a link can be viewed as *direct *if it is impossible to find a subsystem *S*_*z *_for which the following is true

1. *S*_*x *_is hierarchically linked to *S*_*z*_

2. *S*_*z *_is hierarchically linked to *S*_*y*_

3. There exists attractors *A*_1 _and *A*_2 _for which

**(a) ***S*_*y *_occurs in *A*_1 _and *A*_2_

**(b) ***S*_*z *_occurs in *A*_1 _but not *A*_2_

**(c) ***S*_*x *_occurs in neither *A*_1 _nor *A*_2_

This terminology can easily be extended to collections of subsystems.

### Robustness of attractors and subsystems

Suppose *S *is a subsystem that involves a set of nodes *N *and occurs in a set of attractors ℂ. For any network state **z**_*k *_= {*s*_1_, ..., *s*_*v*_} in any attractor *A *∈ ℂ, a node *n*_*f *_can have its state *s*_*f *_perturbed to (*s*_*f *_+ 1) (mod 2), to give a new network state $z_{k}^{\left(f\right)}$ (say). By looking at how often such a network state $z_{k}^{\left(f\right)}$ converges to an attractor *A' *∈ ℂ containing the same subsystem, it is possible to measure how robust the subsystem *S *is to perturbations in the system.

Let *r*(*S*, *L*) be the probability that perturbing the state of a node *n*_*f *_∈ *L *in an attractor *A *∈ ℂ causes the system to converge to an attractor *A' *∈ ℂ. i.e.

$$r(S,L)=\frac{\sum_{A\in \mathbb{C}}\sum_{z_{k}\in A}\sum_{n_{f}\in L}\Omega (S,z_{k},n_{f})}{\left|\mathbb{C}\right|\times \left|A\right|\times \left|L\right|},$$

where

$$\Omega (S,z_{k},n_{f})=\{\begin{array}{ll} 1 & \text{if }z_{k}^{\left(f\right)}\text{ converges to an attractor }A^{\prime }\in \mathbb{C}\\ 0 & \text{otherwise}. \end{array}$$

Then, using this measure, it is possible to measure *Global Robustness *= *r*(*S*, *V*), *Internal Robustness *= *r*(*S*, *N*), and *External Robustness *= *r*(*S*, *M*) (where *V *is the set of all nodes and *M *= *V*\*N*). This allows one to distinguish the effects of perturbing nodes within and outside of the subsystem. The measure *r*(*S*, *L*) can also be adapted to give the robustness of an individual attractor *A *(by letting *S *= *A*, ℂ = {*A*} and *L *= *V*).

## Results: Application to the segment polarity network

**Figure 4:**
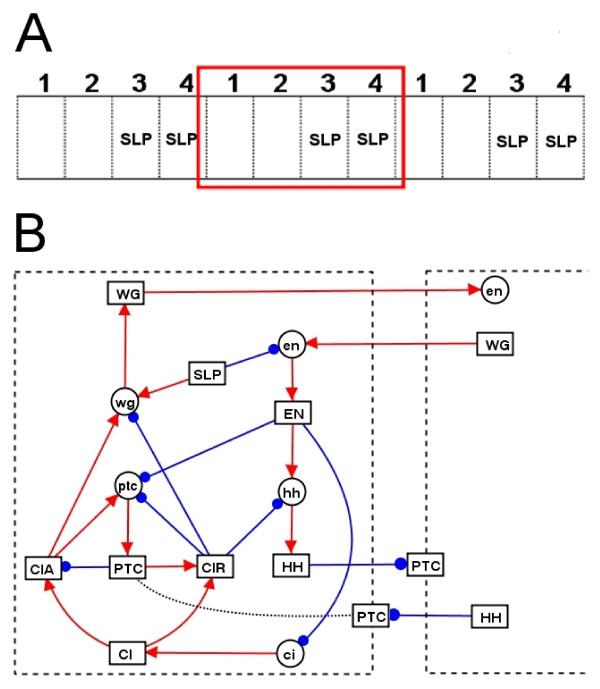
**Summary of the segment polarity network model**. (A) 1 dimensional representation of embryonic epidermis, where each block of 4 cells corresponds to one parasegment in the embryo. The only constraint on the state of each node is that the pair rule protein SLP is only expressed in cells 3 and 4 of each parasegment, because of an earlier developmental stage [14]. (B) Diagram representing the main interactions involved in the *Drosophila *segment polarity network model from [10,11]. Lines with red arrows/blue dots correspond to activation/inhibition (respectively). PTC is found on the cell surface but can trigger intra-cellular interactions. Inter-cellular interactions occur across cell boundaries.

**Figure 5:**
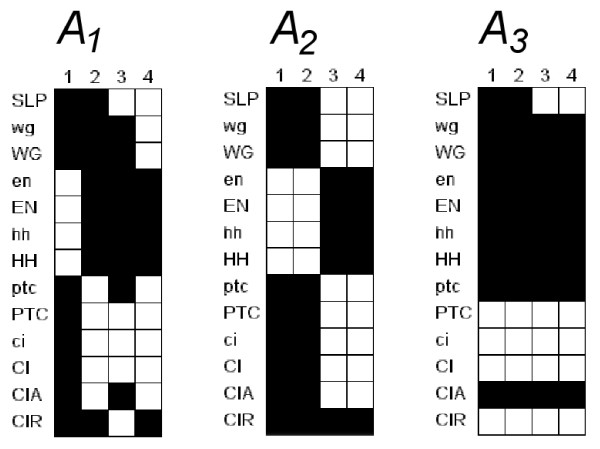
**Observed attractors for the segment polarity network model**. Attractors for the 4 cell model, where each column corresponds to a cell and each row corresponds to a gene/protein (node). Each attractor is a fixed state where each node has state 1 (= white) or 0 (= black). (*A*_1_) Attractor corresponding to the wild-type embryo. (*A*_2_, *A*_3_) Attractors corresponding to other experimentally observed phenotypes.

During the development of the fruit fly *Drosophila melanogaster*, the embryo becomes segmented, with the segment polarity genes responsible for specifying the number, spacing and polarity (direction) of these segments. In order to demonstrate the applicability of our technique to genetic regulatory systems, we analyse a previously published Boolean network model of the *Drosophila *segment polarity network from references [10] and [11]. This model corresponds to a 4-cell ring, where intercellular interactions are allowed between cells 1 and 4. This approach was kept here since the segments start off 4 cells wide and the wild type patterns repeat every 4 cells (before cell proliferation). The Boolean functions for this model are shown in Table S4.1 of Additional file 4, whilst the main interactions are summarised in Fig. 4. This 4 cell model has 10 fixed point attractors. The wild type attractor *A*_1 _is shown in Fig. 5, and is characterised by WG and EN/HH expression in 1 cell wide stripes either side of the parasegment boundary (in cell 4 and 1 resp). Two other attractors, *A*_2 _and *A*_3_, correspond to experimentally observed phenotypes and are also shown in Fig. 5. All 10 attractors are shown in Additional file 4 (Fig. S4.1).

**Figure 6:**
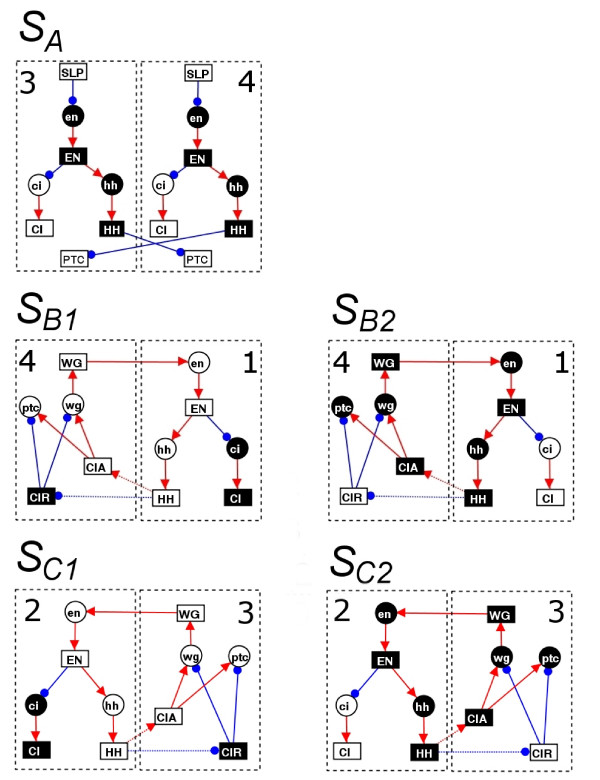
**Main subsystems for the segment polarity network model**. Diagrams representing the 5 main subsystems for the model (*S*_*A*_, *S*_*B*1_, *S*_*B*2_, *S*_*C*1_, *S*_*C*2_). Each diagram shows the nodes, states (white = 1, black = 0) and cells involved in the subsystem. Interactions between the nodes are also shown (red arrow = activation, blue dot = inhibition, dashed line = indirect interaction). All subsystems are fixed states. There are 14 other minor subsystems for this model (*S*_*D*1 _- *S*_*J*2_), which are shown in Table 5.

Applying our method, we identified 19 subsystems that satisfied Definition 6, which are shown in Fig. 6 and Table 5. *S*_*A*_, which occurs in all 10 attractors, corresponds to the cellular response to SLP (expressed in cells 3 and 4 only). Of the remaining subsystems, *S*_*B*1_, *S*_*B*2_, *S*_*C*1 _and *S*_*C*2 _appear to be the most interesting since they capture a large proportion of the global dynamics. They correspond to different states and different cells associated with the same positive feedback loop, involving Wingless (WG), Engrailed (EN), Hedgehog (HH) and Cubitus interruptus (CIA). The feedback loop can either be ON (*S*_*B*1_, *S*_*C*1_) or OFF (*S*_*B*2_, *S*_*C*2_) and either involve inter-cellular interactions between cells 4 and 1 (*S*_*B*1_, *S*_*B*2_) or cells 2 and 3 (*S*_*C*1_, *S*_*C*2_). As mentioned earlier, the wild type attractor is characterised by WG and EN/HH expression in 1 cell wide stripes either side of the parasegment boundary (in cell 4 and 1 respectively). This is captured by *S*_*B*1_.

**Table 5:** Other subsystems for the segment polarity network model

Subsystem	States
*S*_*D*1_	*wg*_1 _= 1, *WG*_1 _= 1
*S*_*D*2_	*wg*_1 _= 0, *WG*_1 _= 0
*S*_*E*1_	*wg*_2 _= 1, *WG*_2 _= 1
*S*_*E*2_	*Wg*_2 _= 0, *WG*_2 _= 0
*S*_*F*1_	*PTC*_1 _= 1
*S*_*F*2_	*PTC*_1 _= 0
*S*_*G*1_	*PTC*_2 _= 1
*S*_*G*2_	*PTC*_2 _= 0
*S*_*H*1_	*CIA*_1 _= 1, *ptc*_1 _= 1
*S*_*H*2_	*CIA*_1 _= 0, *ptc*_1 _= 0
*S*_*I*1_	*CIA*_2 _= 1, *ptc*_2 _= 1
*S*_*I*2_	*CIA*_2 _= 0, *ptc*_2 _= 0
*S*_*J*1_	*CIR*_1 _= 1, *CIR*_2 _= 1
*S*_*J*2_	*CIR*_1 _= 0, *CIR*_2 _= 0

Table showing the remaining 14 subsystems for the segment polarity network model. For each subsystem, the second column of the table shows the nodes, cells (subscript) and states involved. The five main subsystems (*S*_*A*_, *S*_*B*1_, *S*_*B*2_, *S*_*C*1 _and *S*_*C*2_) are shown in Fig. 6.

A previous study has considered the modular design of this system [12], but there are significant differences in the component 'modules'. In [12], modules were arbitrarily chosen to be important intra-cellular pathways involving (a) SLP, (b) EN/HH and (c) WG/CI. However, here we have found that the most important subsystems are inter-cellular combinations of these pathways. Firstly, (a) and (b) combine to form *S*_*A*_. Secondly, (b) and (c) combine across cell boundaries to give 2-cell positive feedback loops *S*_*B*1_, *S*_*B*2_, *S*_*C*1 _and *S*_*C*2_. Moreover, the method in the paper captures the states of these pathways.

**Figure 7:**
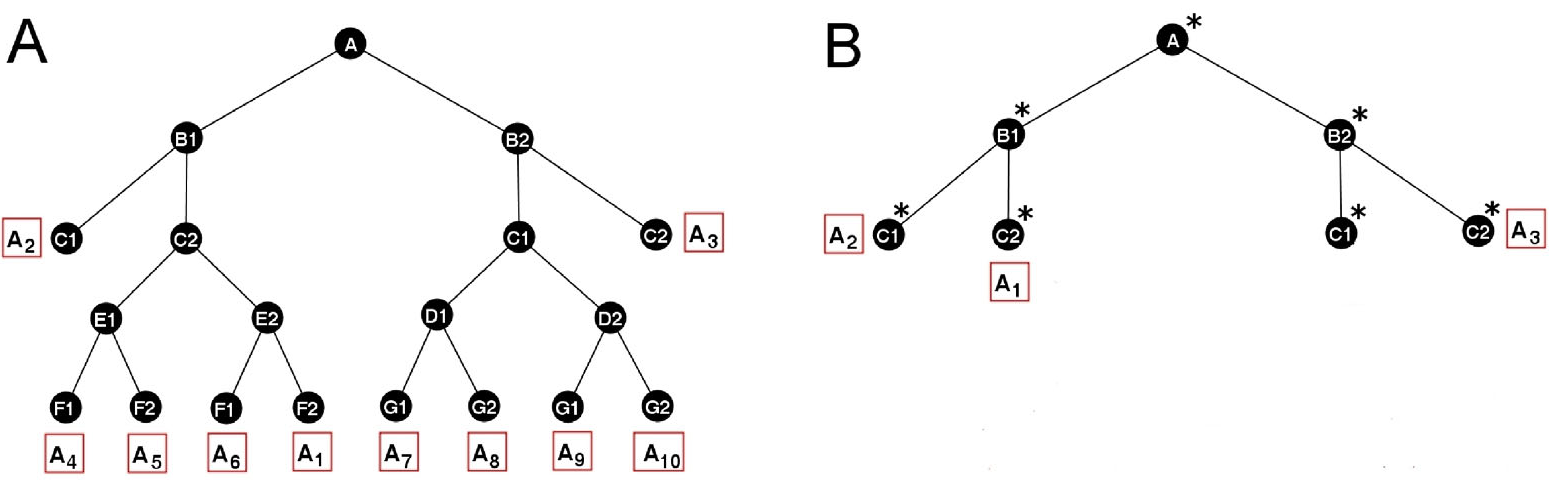
**Breakdown of attractors for the segment polarity network model**. (A) Diagram showing the subsystems involved in specifying each of the attractors *A*_1_, ..., *A*_10 _in the model (attractors shown in Additional file 4). For each path (from top to bottom), that collection of subsystems is unique to the attractor at the bottom of the path. (B) The 5 main subsystems are also sufficient to specify the 3 experimentally observed attractors *A*_1_, *A*_2 _and *A*_3_. These 5 subsystems are composed of subsystems from the original set. $S_{A}^{*}$ = *S*_*A*_∪ *S*_*D*2 _∪ *S*_*E*2 _∪ *S*_*H*2_; $S_{B1}^{*}$ = *S*_*B*1 _∪ *S*_*F*2 _∪ *S*_*J*2_; $S_{B2}^{*}$ = *S*_*B*2 _∪ *S*_*F*1 _∪ *S*_*J*1_; $S_{C1}^{*}$ = *S*_*C*1 _∪ *S*_*G*2_; $S_{C2}^{*}$ = *S*_*C*2 _∪ *S*_*G*1_

Interactions exist between these 19 subsystems, which explain how each one is regulated and which of the 10 attractors they occur in. For each of the 19 subsystems, Table 6 shows the sets of subsystems that *regulate *them, by ensuring their occurrence in an attractor (see previous section). Of the 19, the five subsystems highlighted in Fig. 6 play the largest role in regulating other subsystems with *S*_*A*_, *S*_*B*1_, *S*_*B*2_, *S*_*C*1 _and *S*_*C*2 _involved in the regulation of 12, 9, 9, 9 and 9 subsystems (respectively). Using these five main subsystems as a starting point, it is possible to generate a hierarchical breakdown of the 10 attractors in the model (Fig. 7A). The 10 attractors all contain *S*_*A*_, then split up into 4 main groups depending on the occurrence of *S*_*B*1_, *S*_*B*2_, *S*_*C*1 _and *S*_*C*2_. In two cases, where S_1 _= {*S*_*B*1_, *S*_*C*1_} or S_2 _= {*S*_*B*2_, *S*_*C*2_} occur, this is sufficient to distinguish the attractors *A*_2 _and *A*_3 _(respectively) from the others. In the remaining two cases, where S_3 _= {*S*_*B*1_, *S*_*C*2_} and S_4 _= {*S*_*B*2_, *S*_*C*1_} occur, two additional levels of specification determine the individual attractors. Additional file 4 provides further details of the interactions involved in specifying each individual attractor (and ensuring $S_{1},...,S_{4}$ occur in an attractor).

**Table 6:** Regulation of subsystems in the segment polarity network model

Subsystem	Regulation set
*S*_*A*_	S = {*S*_*A*_, *S*_*B*1_, *S*_*C*1_}
	S = {*S*_*A*_, *S*_*B*2_, *S*_*C*1_}
	S = {*S*_*A*_, *S*_*B*1_, *S*_*C*2_}
	S = {*S*_*A*_, *S*_*B*2_, *S*_*C*2_}
*S*_*B*1_	S = {*S*_*A*_, *S*_*B*1_}
*S*_*B*2_	S = {*S*_*A*_, *S*_*B*2_, *S*_*E*2_}
	S = {*S*_*A*_, *S*_*B*2_, *S*_*C*2_, *S*_*F*1_, *S*_*G*1_}
*S*_*C*1_	S = {*S*_*A*_, *S*_*C*1_}
*S*_*C*2_	S = {*S*_*A*_, *S*_*C*2_, *S*_*D*2_}
	S = {*S*_*A*_, *S*_*B*2_, *S*_*C*2_, *S*_*F*1_, *S*_*G*1_}
*S*_*D*1_	S = {*S*_*D*1_, *S*_*H*1_, *S*_*J*2_}
*S*_*D*2_	S = {*S*_*D*2_}
	S = {*S*_*B*1_}
	S = {*S*_*E*1_}
	S = {*S*_*A*_, *S*_*C*2_, *S*_*F*1_}
*S*_*E*1_	S = {*S*_*E*1_, *S*_*I*1_, *S*_*J*2_}
*S*_*E*2_	S = {*S*_*E*2_}
	S = {*S*_*C*1_}
	S = {*S*_*D*1_}
	S = {*S*_*A*_, *S*_*B*2_, *S*_*G*1_}
*S*_*F*1_	S = {*S*_*B*2_, *S*_*C*1_}
	S = {*S*_*A*_, *S*_*C*2_, *S*_*F*1_}
*S*_*F*2_	S = {*S*_*F*2_, *S*_*H*2_}
	S = {*S*_*B*1_, *S*_*C*1_}
*S*_*G*1_	S = {*S*_*B*1_, *S*_*C*2_}
	S = {*S*_*A*_, *S*_*B*2_, *S*_*G*1_}
*S*_*G*2_	S = {*S*_*G*2_, *S*_*I*2_}
	S = {*S*_*B*1_, *S*_*C*1_}
*S*_*H*1_	S = {*S*_*B*2_, *S*_*C*1_}
*S*_*H*2_	S = {*S*_*B*1_}
	S = {*S*_*E*1_}
	S = {*S*_*A*_, *S*_*C*2_, *S*_*F*1_}
*S*_*I*1_	S = {*S*_*B*1_, *S*_*C*2_}
*S*_*I*2_	S = {*S*_*C*1_}
	S = {*S*_*D*1_}
	S = {*S*_*A*_, *S*_*B*2_, *S*_*G*1_}
*S*_*J*1_	S = {*S*_*A*_, *S*_*B*2_, *S*_*C*2_, *S*_*F*1_, *S*_*G*1_}
*S*_*J*2_	S = {*S*_*B*1_}
	S = {*S*_*C*1_}
	S = {*S*_*F*2_, *S*_*G*2_}

Table showing the sets of subsystem collections responsible for regulating each individual subsystem *S*_*y*_. Each row shows a collection of subsystems (S) that can trigger the occurrence of *S*_*y*_.

Since only 3 of the 10 attractors from the model correspond to observed phenotypes, it is important to ensure that the main subsystems aren't an unrealistic artefact of the model. Re-applying our method of identifying subsystems to the reduced set of observed attractors {*A*_1_, *A*_2_, *A*_3_}, we find that the 5 main subsystems in Fig. 6 are preserved. The only change is that the new subsystems incorporated some of the old smaller subsystems. In particular we get the following 5 main subsystems

- $S_{A}^{*}$ = *S*_*A *_∪ *S*_*D*2 _∪ *S*_*E*2 _∪ *S*_*H*2_

- $S_{B1}^{*}$ = *S*_*B*1 _∪ *S*_*F*2 _∪ *S*_*J*2_

- $S_{B2}^{*}$ = *S*_*B*2 _∪ *S*_*F*1 _∪ *S*_*J*1_

- $S_{C1}^{*}$ = *S*_*C*1 _∪ *S*_*G*2_

- $S_{C2}^{*}$ = *S*_*C*2 _∪ *S*_*G*1_

Moreover, as can be seen in Fig. 7B, the 5 main subsystems still provide a breakdown of the attractors. Focusing on subsystems allows us to look at the segment polarity network from a different angle. The first thing that stands out is that the subsystems exhibit a symmetry across the cell 1/2 boundary, whereby every subsystem in cell 4/1 has a *symmetric counterpart *in cell 3/2 (compare the pairs *S*_*A*_-*S*_*A*_, *S*_*B*1_-*S*_*C*1_, *S*_*B*2_-*S*_*C*2_, *S*_*D*1_-*S*_*E*1_, *S*_*D*2_-*S*_*E*2_, *S*_*F*1_-*S*_*G*1_, *S*_*F*2_-*S*_*G*2_, *S*_*H*1_-*S*_*I*1_, *S*_*H*2_-*S*_*I*2_, *S*_*J*1_-*S*_*J*1 _and *S*_*J*2_-*S*_*J*2_). Moreover, as can be seen in Table 6, these symmetric counterparts are analogously regulated. The protein SLP is crucial to setting up this symmetry, since it sets off a chain of interactions that nullifies *hh*, HH and *en *in cells 3 and 4 (see *S*_*A *_in Fig. 6). This then blocks signalling between cells 3 and 4, since (1) HH and WG are the only proteins involved in cell – cell signalling (in this model) and (2) *en *is the only gene (in this model) that can receive signals from WG. Therefore, it appears that one of the effects of SLP is to partition the cells in the embryo into 'isolated' 4 cell wide blocks (at this developmental stage) with the cell 1/2 boundary at the centre of this block. SLP also imposes restrictions on interactions within the neighbouring cells 1 and 2. This appears to be sufficient to partition the 4 cells into two, 2-cell blocks (cell 4/1 and cell 2/3) that have relatively independent sub-dynamics. These two, 2-cell blocks are forced to choose dynamics from *S*_*B*1_, *S*_*B*2_, *S*_*C*1 _and *S*_*C*2_, which in turn specify the attractor chosen. Important interactions do occur across the 1/2 cell boundary and sub-dynamics are conserved across this boundary. However, the driving force behind the dynamics and specification of the network appear to come from the two, 2-cell wide blocks. Whether this symmetry is inherent in the system or an artefact of the model remains to be verified, since only three attractors have been observed experimentally, and so more data may be required. In order to gain added insight into both the subsystems and the attractors, we calculate the robustness of each attractor and subsystem (see Tables 7 and 8). The robustness score is between 0 and 1 and measures how often the attractor/subsystem can survive after a perturbation to any node state. *S*_*B*1 _and *S*_*C*1 _are maximally robust in that no single node perturbations can destroy them. This also implies that *S*_*B*1 _and *S*_*C*1 _draw in local sub-dynamics that only marginally differ from them. Therefore, since *S*_*B*1 _and *S*_*C*1 _both occur in *A*_2_, this partially explains why *A*_2 _is so dominant in the state space, with over 98 % of network states converging to it. On the other hand *S*_*B*2 _and *S*_*C*2 _are vulnerable to perturbations, especially to nodes within the subsystems themselves. The wild type attractor is *A*_1_, which contains *S*_*B*1 _and *S*_*C*2_. Only a small proportion of the state space (0.01 %) converges to this attractor but it is still relatively robust (0.67). It appears that this robustness is primarily due to *S*_*B*1_, the same subsystem responsible for the characteristic WG and EN/HH expression either side of the parasegment boundary (in cell 4 and 1 resp).

**Table 7:** Robustness of attractors in the segment polarity network model

Attractor	Basin of attraction	Robustness
*A*_1_	0.01 %	0.67
*A*_2_	98.63 %	1.0
*A*_3_	1.27 %	1.0
*A*_4_	0.00035 %	0.60
*A*_5_	0.00005 %	0.58
*A*_6_	0.04 %	0.69
*A*_7_	0.0003 %	0.60
*A*_8_	0.00015 %	0.58
*A*_9_	0.04 %	0.69
*A*_10_	0.01 %	0.67

Robustness of each attractor, where the score is between 0 and 1 and defined in the *Results *section. We also give the percentage of state space converging to each attractor (estimated from a sample of 2,000,000 initial network states).

**Table 8:** Robustness of subsystems in the segment polarity network model

Subsystem	Robustness		
	Global	Internal	External
*S*_*A*_	1.0	1.0	1.0
*S*_*B*1_	1.0	1.0	1.0
*S*_*B*2_	0.78	0.56	0.85
*S*_*C*1_	1.0	1.0	1.0
*S*_*C*2_	0.78	0.56	0.85
*S*_*D*1_	0.63	0.5	0.63
*S*_*D*2_	0.99	0.88	1.0
*S*_*E*1_	0.63	0.5	0.63
*S*_*E*2_	0.99	0.88	1.0
*S*_*F*1_	0.76	0.71	0.76
*S*_*F*2_	0.97	0.33	0.99
*S*_*G*1_	0.76	0.71	0.76
*S*_*G*2_	0.97	0.33	0.99
*S*_*H*1_	0.73	1.0	0.72
*S*_*H*2_	1.0	1.0	1.0
*S*_*I*1_	0.73	1.0	0.72
*S*_*I*2_	1.0	1.0	1.0
*S*_*J*1_	1.0	1.0	1.0
*S*_*J*2_	1.0	1.0	1.0

Robustness of each subsystem, where the score is between 0 and 1 and defined in the *Results *section.

## Discussion and Conclusions

Described in this paper is a framework for identifying subsystems ('dynamical modules') from a Boolean network model. This method of identifying subsystems is applicable for systems with either fixed point attractors, cyclic attractors or both. The methods are designed to be applicable to genetic regulatory systems. Therefore, we have applied the method to an existing model of the *Drosophila *segment polarity network. We identify novel subsystems acting across cell boundaries, and demonstrate how they regulate each other to give the attractors. Analysis of the subsystems also allows us to predict which ones underlie robustness in the wild type attractor.

Our methods are ideally suited to analysing multi-stable systems, where different stable states/conditions are captured within different attractors. For a node set with two or more different dynamics in multiple attractors, the different dynamics will form part of different intersection sequences, partition sequences and subsystems. Therefore, some identified subsystems will capture the key components associated with particular stable states (for some set of nodes). Meanwhile, other subsystems will capture shared/conserved dynamics. For example, in the *Drosophila *segment polarity network model, subsystems *S*_*B*1_, *S*_*B*2_, *S*_*C*1 _and *S*_*C*2 _(Fig. 6) capture different states of 2 positive feedback loops, which underlie multistationarity in this particular system. On the other hand, the subsystem *S*_*A *_captures a conserved sub-state that can co-occur alongside all of the remaining subsystems.

Once the subsystems have been identified, they can provide novel insight into aspects of the model. Firstly, Boolean functions from the model can be used to determine how each subsystem is regulated. This knowledge can then be used to provide a more detailed hierarchical breakdown of the original attractors, allowing the key differences and similarities between different attractors to be highlighted. A second advantage of looking at subsystems is that they can provide a new way of looking at robustness and adaptability. An external perturbation may switch the system from one attractor to another. However, despite this, a subsystem may be robust and remain unchanged. Looking at the effect of external perturbations on subsystems can highlight which parts of the system are most robust, or how certain subsystems can adapt/change without affecting others.

Although we have focussed on Boolean network models in this paper, the method can be adapted to other discrete-state, discrete-time models/systems. Moreover, since subsystems (in our method) are found directly from the attractors, a mathematical model or underlying network topology is not even necessarily required to identify them. Hierarchical links (see *Results*) between these subsystem can also be found without a mathematical model. These links allow dependencies between the subsystems to be discovered, providing insight into potential functional links. However, to gain a full understanding of how the subsystems interact, and the nature of these links, details of interactions between nodes (genes/proteins) are currently required. Importantly, our method can be applied directly to data on the attractors of a system when these data are incomplete. This can result either from only a subset of the full set of attractors being known, or from information being available for only a subset of nodes. In these cases, the method will still break up the attractors and identify relevant sub-dynamics. However, the more attractors there are, the more detailed and informative the eventual subsystems will be.

For many systems, data may be incomplete and/or noisy, leading to models that are also incomplete and/or unreliable. Even in the case of the extensively studied *Drosophila *segment polarity network, components may be missing and not fully understood. The most important factor affecting the quality of the results from our method (i.e. whether we find useful subsystems) is the reliability of the data/attractors. Even if there are some inaccuracies in a model or network topology, we can still make some reliable assertions, by focussing on the reliable data/attractors. In the case of the *Drosophila *segment polarity network model studied in this paper, 3 of the 10 attractors correspond to observed phenotypes. As we have already shown, applying the method directly to these 3 attractors gives us 5 'primary' subsystems ($S_{A}^{*},S_{B1}^{*},S_{B2}^{*},S_{C1}^{*},S_{C2}^{*}$, see Fig. 7B). Then, once extra attractors from any model are taken into account, these 'primary' subsystems are split up so that each new subsystem only comes from a single 'primary' subsystem (e.g. in the existing model, the subsystem $S_{A}^{*}$ splits into *S*_*A*_, *S*_*D*2_, *S*_*E*2_, *S*_*H*2_). Therefore, for any future model of this system (with extra components or interactions), the subsystems obtained from our method should also relate back to these 5 primary subsystems $S_{A}^{*},S_{B1}^{*},S_{B2}^{*},S_{C1}^{*},S_{C2}^{*}$. Therefore, modules extracted from the currently available data/models are still informative, and those that only rely on observed data can be viewed as reliable (even if they are larger/less detailed than the 'true' subsystems). New data and improved models will just increase the precision of the results/subsystems. Another way in which we can assess subsystems (from a current model) is by looking at how robust they are. As well as looking at how robust each subsystem is to perturbations in node states, we can assess how robust each subsystem is to changes in the model itself (such as changes in Boolean functions or the addition of new components). We envisage that this method can be extended in a number of ways. Firstly, the method can be applied to signal transduction pathways by having '*n' *attractors representing the state of the pathway(s) under *'n' *different environmental/cellular conditions. These attractors can then be analysed with the new method to identify subsystems within pathway(s). Additionally, there is no need to restrict ourselves to studying cellular systems and we envisage that the method will be applicable in other fields.

The main method we have introduced could be applied directly to large scale datasets, to extract novel functional information. For an individual experiment, data on the state of a set of nodes (genes/proteins) are taken from a particular tissue, developmental stage and environmental condition (e.g. normal, high/low temperature, light/dark). Moreover, expression data are typically taken at discrete time points and are often converted to binary (e.g. 1 = 'expressed', 0 = 'not expressed'). Therefore, the results of each individual experiment would correspond to an individual discrete-state discrete-time attractor. Although we have not applied our method to a large datasets in this paper, we believe that following methodology could be used as a starting point

1. Carry out multiple experiments in different environmental conditions/tissues/developmental stages, to give a range of discrete-state attractors,

2. Apply the method described in this paper, directly to this set of attractors (without using any model).

Then, identified subsystems would then be subsets of proteins, whose collective dynamics are conserved across data sets. In principle, this method could be applied to either time series data or fixed point data. However, since it is more difficult to obtain accurate data on time courses rather than steady states, we believe that the approach would be most likely to yield valuable information for fixed point/steady state data. We note here, that data from mutants are not necessary for such an approach. In fact, mutant data may not be as suitable, since each mutant would correspond to a different system. This type of analysis would be complementary to clustering, which primarily looks for groups of genes whose expression levels change in unison.

Reliability of data will be a major issue if trying to apply this method directly to experimental data. Expression data are very noisy and the less reliable the data, the less certain we will be about any identified subsystems. However, if can still be the case that novel groupings of genes/proteins could be found. Moreover, in the future, we envisage that these methods can be adapted so that we estimate the 'probability' of certain partial state sequences occurring in attractors, and then use this data to identify likely subsystems.

One limitation of this method is that it may have difficulties with systems with lots of cyclic attractors, each one containing a similar (but not identical) sub-dynamic. This is because we will get lots of similar intersection sequences (Definition 4). Since this definition provides the hierarchical backbone of the method, the method could struggle to identify informative partition sequences (Definition 5) or subsystems (Definition 6).

The method works best when (1) every attractor is a fixed point or (2) different cyclic sub-dynamics interact hierarchically or independently. This implies that the dynamics are partitioned in a strict hierarchical manner and there is no ambiguity when selecting subsystems. In case (1), an additional advantage is that all the fixed point attractors still occur when any asynchronous/stochastic updating scheme is used in a Boolean network model (once the model reaches a fixed point attractor, no update can cause the system to leave that attractor). Therefore, since subsystems are found directly from the attractors, the subsystems would also be the same. The situation of having all fixed point attractors is often relevant when considering cell type specification in developmental systems, where a cell must settle on one of a number of fixed states.

## Methods

### Boolean network models

For the purposes of this paper, a Boolean network model is a discrete time, deterministic, synchronous process acting on a directed network of *v *nodes *V *= {*n*_1_, ..., *n*_*v*_}. At each discrete time step *t *≥ 0, each node *n*_*i *_∈ *V *has a Boolean state *s*_*i*_(*t*) ∈ {0, 1} and these collectively form a network state **x **= **x**(*t*) = (*s*_1_(*t*), ..., *s*_*v*_(*t*)). The model progresses, from one time step to the next, by synchronously updating these Boolean states according to a set of Boolean functions **f **= (*f*_1_, ..., *f*_*v*_), as follows

**- x**(*t *+ 1) = **f**(**x**(*t*)) = (*f*_1_(**x**(*t*)), ..., *f*_*v*_(**x**(*t*))).

As time progresses, **x**(*t*) eventually gets trapped in an *attractor A *= {**z**_0_, ..., **z**_*p*-1_}. i.e. there is a time point *t' *for which

- For all *t *≥ *t'*, **x**(*t*) = **z**_*k *_(where *k *= *t *- *t' *(mod *p*)).

Here, each **z**_*i *_∈ *A *is called an *attractor state*

For a given model, there are typically multiple attractors and these correspond to the stable dynamics of the system. For the purposes of this paper A = {*A*_1_, ..., *A*_*r*_} is the set of all attractors. A method to identify every attractor in a given Boolean network model is given in [13]. An example of a Boolean network model, along with a sample of its attractors, can be seen in Fig. 2.

It is possible to have models where nodes are updated in an asynchronous fashion. The method of identifying subsystems (below) can still be applied to such models as long as you focus on a specific set of attractors associated with such an updating scheme. The case where node updates are chosen stochastically is not explicitly considered here.

### Algorithms

Below, we describe the main algorithms used in our new method. As can be seen in Fig. 1, there are multiple procedures/steps in our method, and so we describe each of these steps separately; namely

- Identify every *intersection sequence *(satisfying Definition 4),

- Identify every *partition sequence *(satisfying Definition 5),

- Identify every *subsystem *(satisfying Definition 6).

Finally, if a suitable mathematical model is available, we show how *regulation sets *can be found for each subsystem (satisfying Definition 7). Formal proofs for all of the procedures can be found in Additional file 1 (Main Method) and Additional file 2 (Regulation of Subsystems).

However, before describing the main procedure, we need to be able to identify the partial state sequences that occur in each attractor, given a node set *N*. Therefore, we first describe some procedures relating to partial state sequences.

### Algorithms: Partial state sequences

Given a node set *N *and an attractor *A *= {**z**_0_, **z**_1_, ..., **z**_*p*-1_}, the following procedure identifies a partial state sequence $P=\{x_{0}^{N},...,x_{q-1}^{N}\}$ that occurs in *A *(i.e. the 3 properties of Definition 3 are satisfied)

Procedure 1.

Initially let *k *= 0, *b*_0 _= 0 and $x_{0}^{N}$ = {*s*_*i *_∈ **z**_0 _: *n*_*i *_∈ *N*}. The enter the following loop

**Step 1: **If *k *= *p *- 1, let q* = *b*_*p*-1 _+ 1 and **go to **step 6

**Step 2: **Let *j *= *k *and increment *k *by 1 (let *k *= *k *+ 1)

**Step 3: **If $x_{b_{j}}^{N}$ = {*s*_*i *_∈ **z**_*k *_: *n*_*i *_∈ *N*}, then let *b*_*k *_= *b*_*j *_and **go to **step 1 (otherwise **go to **step 4)

**Step 4: **Let *b*_*k *_= *b*_*j *_+ 1

**Step 5: **Let $x_{b_{k}}^{N}$ = {*s*_*i *_∈ **z**_*k *_: *n*_*i *_∈ *N*} and **go to **step 1

**Step 6: **If $x_{b_{p-1}}^{N}=x_{b_{0}}^{N}$**and **q* > 1, reduce q* by 1 (let q* = q* - 1)

**Step 7: **Let *q *be the smallest integer for which both

**(a) ***q*|q* (this can be *q *= q*)

**(b) **$x_{f}^{N}=x_{g}^{N}$, whenever *f *≤ *b*_*p*-1_, *g *≤ *b*_*p*-1 _and *f *(mod *q*) = *g *(mod *q*)

**Step 8: **For *k *= 0, ..., *p *- 1, let *b*_*k *_= *b*_*k *_(mod *q*)

end of procedure

A formal proof for this procedure can be be found in Additional file 1 (see Theorem S1.4 in section S1.1.1). However, here, we give a brief justification.

At the end of this procedure $P=\{x_{0}^{N},...,x_{q-1}^{N}\}$ occurs in *A *and the 3 properties of Definition 3 are satisfied. Steps 2–5 ensure that properties 1 and 2 are satisfied and the partial states in *P *cycle within the attractor *A *in the correct order (as the attractor progresses over time). Steps 3, 6, 7 and 8 ensures property 3, so that *P *is the smallest possible set of partial states that cycles within *A*. In particular

**(a) **Steps 3 and 6 ensures no two adjacent partial states in *P *are identical

**(b) **Step 7 ensures that if a sequence of states cycles many times within an attractor, only one copy is kept.

This leaves a partial state sequence that just describes the 'order' in which the node states change in *A *(for nodes in *N*).

This procedure can be easily modified to look at a partial state sequence $P_{x}=\{x_{0}^{N},x_{1}^{N},...,x_{q-1}^{N}\}$ (for a node set *N*) occurring in another partial state sequence $P_{y}=\{y_{0}^{M},...,y_{r-1}^{M}\}$ (where *M *⊇ *N*).

Given a node set *N *and a set of attractors ℂ, we need to be able to find a set of partial state sequences *P*_1_, ..., *P*_*k *_that are all distinguishable from one another and optimally partition ℂ into smaller sets ${\mathbb{C}}_{1},...,{\mathbb{C}}_{k}$.

One such way is to apply the following procedure (Procedure 2). This will identify partial state sequences *P*_1_, ..., *P*_*k *_and sets of attractors ${\mathbb{C}}_{1},...,{\mathbb{C}}_{k}$ that satisfy properties **A**- **F **below

**A: **For *i *= 1, ..., *k*, *P*_*i *_involves the node set *N *(i.e. $P_{i}=\{x_{i_{0}}^{N},...,x_{i_{q-1}}^{N}\}$)

**B: **For *i *= 1, ..., *k*, *P*_*i *_occurs in every attractor *A *∈ ℂ_*i*_

**C: **For *i *= 1, ..., *k*, *P*_*i *_does not occur in any attractor *A *∉ ℂ_*i*_

**D: **For any *i*, *j *(1 ≤ *i *<*j *≤ *k*), ${\mathbb{C}}_{i}\cap {\mathbb{C}}_{j}=\emptyset$

**E: **${\mathbb{C}}_{1}\cup ...\cup {\mathbb{C}}_{k}=\mathbb{C}$

**F: **Given the node set *N*, there are no other partial state sequences *P' *∉ {*P*_1_, ..., *P*_*k*_} that occur in any attractor *A *∈ ℂ (unless *P' *and some *P*_*i *_contain the same partial states in the same order, within a rotation)

Procedure 2.

Begin with the node set *N *and set of attractors ℂ and then carry out the following steps

**Step 1: **For every attractor *A*_*j *_∈ ℂ, apply Procedure 1 to *N *and *A*_*j*_, to get a partial state sequence *Q*_*j *_that occurs in *A*_*j*_.

**Step 2: **Put the *Q*_*j*_'s into groups *i *= 1, ..., *k*, whereby two partial state sequences ${Q^{\prime }}_{x}$, ${Q^{\prime }}_{y}$ go in the same group ⇔ ${Q^{\prime }}_{x}$ is equivalent to ${Q^{\prime }}_{y}$ (i.e. ${Q^{\prime }}_{x}$ contains the same partial states in the same order, within a rotation). Here, *k *is the minimum number of groups required to hold every *Q*_*j*_.

**Step 3: **For each group, *i*, let

**i) ***P*_*i *_= any *Q*_*j *_in the group *i*

**ii) **ℂ_*i *_= {*A*_*j *_: *Q*_*j *_is part of the group *i*}

end of procedure

A formal proof for this procedure can be be found in Additional file 1 (see Theorem S1.10 in section S1.1.3). However, here, we give a brief justification of why properties **A **– **F **are satisfied at the end. If a partial state sequence $P=\{x_{0}^{N},x_{1}^{N},...,x_{q-1}^{N}\}$ occurs in an attractor *A *then so will *q *- 1 other *equivalent *partial state sequences that contain the same partial states in the same order (within a rotation). e.g. $P^{\prime }=\{x_{1}^{N},...,x_{q-1}^{N},x_{0}^{N}\}$ Moreover, if *P *occurs in an attractor *A *then no other partial state sequences for the same node set *N *can (other than the equivalent ones). Then, because

- Each partial state sequence *Q*_*j *_(from Step 1) occurs in the attractor *A*_*j *_and involves a node set *N*,

- Partial state sequences found in Step 1 are only grouped together (in Step 2) if they are equivalent,

- Attractors are only grouped together in Step 3, if equivalent partial state sequences occur in them,

properties **A**, **B**, **C **and **F **must be satisfied. Properties **D **and **E **are satisfied because each attractor is put into exactly one set in Step 3.

### Algorithms: Intersection sequences (Stage 1)

Identifying every intersection sequence is equivalent to finding every partial state sequence that satisfies the 3 properties of Definition 4 (for some set of attractors ℂ, say).

**Figure 8:**
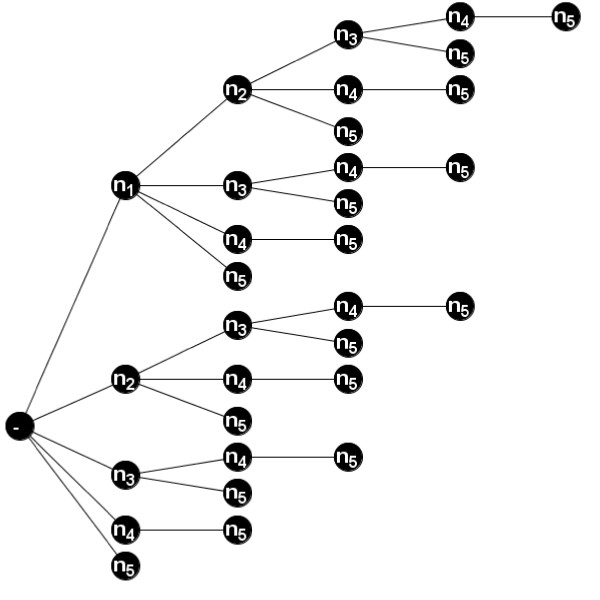
**Identifying Intersection sequences**. Every path from left to right (starting at '-') in this tree represents a different node set *N *⊆ *V *= {*n*_1_, *n*_2_, *n*_3_, *n*_4_, *n*_5_}. It is possible to search this tree and visit every node set *N *⊆ *V *(exactly once). For example, follow the path {*n*_1_} → {*n*_1_, *n*_2_} → {*n*_1_, *n*_2_, *n*_3_} → {*n*_1_, *n*_2_, *n*_3_, *n*_4_} → {*n*_1_, *n*_2_, *n*_3_, *n*_4_, *n*_5_} → {*n*_1_, *n*_2_, *n*_3_, *n*_5_} → {*n*_1_, *n*_2_, *n*_4_} → {*n*_1_, *n*_2_, *n*_4_, *n*_5_} → {*n*_1_, *n*_2_, *n*_5_} → {*n*_1_, *n*_3_} → {*n*_1_, *n*_3_, *n*_4_} → {*n*_1_, *n*_3_, *n*_4_, *n*_5_} → {*n*_1_, *n*_3_, *n*_5_} → {*n*_1_, *n*_4_} → {*n*_1_, *n*_4_, *n*_5_} → {*n*_1_, *n*_5_} → {*n*_2_} → {*n*_2_, *n*_3_} → {*n*_2_, *n*_3_, *n*_4_} → {*n*_2_, *n*_3_, *n*_4_, *n*_5_} → {*n*_2_, *n*_3_, *n*_5_} → {*n*_2_, *n*_4_} → {*n*_2_, *n*_4_, *n*_5_} → {*n*_2_, *n*_5_} → {*n*_3_} → {*n*_3_, *n*_4_} → {*n*_3_, *n*_4_, *n*_5_} → {*n*_3_, *n*_5_} → {*n*_4_} → {*n*_4_, *n*_5_} → {*n*_5_}

The method for identifying every intersection sequence can be visualised by considering the tree in Fig. 8. Searching through a tree analogous to this one (for a network with nodes *V *= {*n*_1_, ..., *n*_*v*_}), means that every node set *N *can be visited at some point. Then, using Procedure 2, we can identify the partial state sequences that occur in each attractor (for each node set *N*). Then, after the tree has been fully examined, we can pick out the partial state sequences (*P*) and sets of attractors ℂ that satisfy the 3 properties of Definition 4. In reality, many branches of the tree can be ignored, leading to improvements in efficiency (discussed below).

We first give the procedure for identifying every intersection sequence, then give an example and finally discuss ways to make the process more efficient.

#### Procedure 3

First, consider the tree in Fig. 8 and note that for every node set *N*, there exists a path from left to right (starting at '*-'*) that corresponds to it. Therefore, searching through a tree analogous to the one in Fig. 8 (for a network with nodes *V *= {*n*_1_, ..., *n*_*v*_}), every node set *N *can be visited at some point.

The procedure searches through the tree (as in Fig. 8) and carries out the following steps for each node set *N*. At the end of the procedure the set **S **contains every intersection sequence

**Step 0 (Initialisation): **Let **S **= ∅ and let *N *= ∅ (-)

**Step 1: **Move onto the next node set *N *in the tree (as in Fig. 8).

**Step 2: **For the node set *N*, apply Procedure 2 to identify partial state sequences *P*_1_, ..., *P*_*k *_and sets of attractors ${\mathbb{C}}_{1},...,{\mathbb{C}}_{k}$ satisfying

**A: **For *i *= 1, ..., *k*, *P*_*i *_involves the node set *N *(i.e. $P_{i}=\{x_{i_{0}}^{N},...,x_{i_{q-1}}^{N}\}$)

**B: **For *i *= 1, ..., *k*, *P*_*i *_occurs in every attractor *A *∈ ℂ_*i*_

**C: **For *i *= 1, ..., *k*, *P*_*i *_does not occur in any attractor *A *∉ ℂ_*i*_

**D: **For any *i*, *j *(1 ≤ *i *<*j *≤ *k*), ${\mathbb{C}}_{i}\cap {\mathbb{C}}_{j}=\emptyset$

**E: **${\mathbb{C}}_{1}\cup ...\cup {\mathbb{C}}_{k}=A$ (the set of all attractors)

**F: **Given the node set *N*, there are no other partial state sequences *P' *∉ {*P*_1_, ..., *P*_*k*_} that occur in any attractor *A *∈ A

**Step 3: **For *i *= 1, ..., *k*, add the pair {*P*_*i*_, ℂ_*i*_} to the set **S**

**Step 4: **For *i *= 1, ..., *k*, check **S **to see if there is any pair {$Q=\{y_{0}^{M},...,y_{r-1}^{M}\},D$} for which either of the following are true

**(a) ***M *⊂ *N *and $D={\mathbb{C}}_{i}$

**(b) ***M *⊃ *N *and $D={\mathbb{C}}_{i}$

If (a) is true, remove {*Q*, D} from **S**. If (b) is true, remove {*P*_*i*_, ℂ_*i*_} from **S**

**Step 5: **If the tree has been completely searched, **end procedure**. Otherwise, return to step 1.

end of procedure

A formal proof for this procedure can be be found in Additional file 1 (see Theorem S1.14 in section S1.2.1). However, here, we give a brief justification.

At the end of the procedure, **S **gives a complete set of intersection sequences (satisfying the 3 properties of Definition 4). Step 2 ensures every partial state sequence that satisfies properties 1 and 2 are identified for each node set *N*. Step 4 then ensures that only those satisfying property 3 remain in **S**.

As an example of how the procedure finds intersection sequences, consider the model in Fig. 2 and the intersection sequence *P*_3 _(from Fig. 3). When the node set *N *= {*n*_2_, *n*_3_, *n*_4_} is analysed in Step 2 of the algorithm (using Procedure 2) we find two distinct partial state sequences that satisfy properties 1 and 2 of Definition 4, namely

- *P*_3 _that intersects at ℂ_3 _= {*A*_1_, *A*_2_, *A*_3_}

- *P*_*x *_= {*s*_2 _= 0, *s*_3 _= 0, *s*_4 _= 0} that intersects at ℂ_*x *_= {*A*_4_}

The remainder of the algorithm involves deciphering whether of these partial state sequences satisfy the final property (Property 3 of Definition 4). This can be done by looking at the way the attractors are split for different node sets. For the node set *N *= {*n*_2_, *n*_3_, *n*_4_} (above) we split the attractors as follows - {*A*_1_, *A*_2_, *A*_3_} + {*A*_4_}. For larger nodes sets *M *⊃ *N*

*M*_1 _= {*n*_1_, *n*_2_, *n*_3_, *n*_4_} splits the attractors as follows - {*A*_1_, *A*_2_} + {*A*_3_} + {*A*_4_}

*M*_5 _= {*n*_2_, *n*_3_, *n*_4_, *n*_5_} splits the attractors as follows - {*A*_1_} + {*A*_2_} + {*A*_3_} + {*A*_4_}

*M*_6 _= {*n*_2_, *n*_3_, *n*_4_, *n*_6_} splits the attractors as follows - {*A*_1_} + {*A*_2_} + {*A*_3_} + {*A*_4_}

etc

Therefore, when *P*_3 _and ℂ_3 _= {*A*_1_, *A*_2_, *A*_3_} are considered in Step 4, it can never be removed from the set **S**. This is because when a larger node set *M *⊃ *N *is considered, we will never have a set of attractors $\mathbb{C}={\mathbb{C}}_{3}$ (or $D⊇{\mathbb{C}}_{3}$). Therefore, *P*_3 _satisfies the property 3 and is an intersection sequence. However, *P*_*x *_is not an intersection sequence since the corresponding set of attractors ({*A*_4_}) is still associated with a larger node set *M *⊃ *N*. Therefore it will be removed from the set **S **in Step 4 (either when *N *or *M *is analysed in the procedure).

We now explain how the procedure can be made more efficient

#### Improving efficiency(1)

Consider node sets *N *⊂ *M *and two partial state sequences $P=\{x_{0}^{N},...,x_{q-1}^{N}\}$ and $P^{\prime }=\{y_{0}^{M},...,y_{r-1}^{M}\}$ that both occur in some attractor *A*. Then, *P' *must contain *P *and hence only occur in an attractor whenever *P *does (proved in Lemma S1.12 of Additional file 1).

Therefore, if *N *⊂ *M *⊂ *V*, and *P *only occurs in a single attractor A*, neither *P *nor *P' *can be intersection sequences. This is since the attractor A* is itself a partial state sequence (for a larger node set *V*) that occurs in exactly the same set of attractors (i.e. just A*), and so property 3 of Definition 4 fails.

Therefore, if Step 2 of Procedure 3 identifies a partial state sequence $P_{i}=\{x_{0}^{N},...,x_{q-1}^{N}\}$ that only occurs in a single attractor ℂ_*i *_= {A*}, we know that re-analysing this attractor {A*} for any node set *M *⊃ *N *is pointless.

We show one way in which this knowledge can improve efficiency in Procedure 3. Suppose, attractors ${A^{\prime }}_{1},...,{A^{\prime }}_{f}$ were returned as single attractors in Step 2, when analysing an earlier node set *P *⊆ *N *(including *P *= *N*). Then, when a path is extended to the right in the tree (Fig. 8) from a node set *N *to a node set *M *⊃ *N*, Procedure 2 in Step 2 need only be applied to the smaller set of attractors

$$A^{\prime }=A\backslash \{{A^{\prime }}_{1},...,{A^{\prime }}_{f}\}$$

Moreover, if **every **attractor is returned as a single attractor in Step 2, when analysing an earlier node set *P *⊆ *N *(including *P *= *N*), there is no need to extend the path to look at node sets *M *⊃ *N*. In Fig. 8, this is equivalent to ignoring all longer paths that include extra nodes to the right. For example, if *N *= {*n*_1_, *n*_3_}, there would be no need to look at longer paths (from left to right) that give node sets *M *= {*n*_1_, *n*_3_, *n*_4_}, *M *= {*n*_1_, *n*_3_, *n*_5_} or *M *= {*n*_1_, *n*_3_, *n*_4_, *n*_5_}.

However, because all of the attractors are intersection sequence, the full node set *V *= {*n*_1_, ..., *n*_*v*_} should still be fully analysed in Steps 2 – 4 (possibly at the very end of the procedure).

#### Improving efficiency(2)

As can be seen in Fig. 8, some nodes appear less than others, with the least frequent nodes visited earlier in the tree. Therefore, it is likely to be advantageous to re-index nodes in the tree during the search. At any stage during the search, nodes along paths to the right (from a node set *N*) can be re-indexed without impairing our ability to search the tree. For example, once *N *= {*n*_1_, *n*_3_} has been reached, re-indexing nodes {*n*_4_, *n*_5_} to {*n*_5_, *n*_4_} still allows us to reach the same node sets *M *= {*n*_1_, *n*_3_, *n*_4_}, *M *= {*n*_1_, *n*_3_, *n*_5_} and *M *= {*n*_1_, *n*_3_, *n*_4_, *n*_5_}, as before. However, they would be visited in a different order (*M *= {*n*_1_, *n*_3_, *n*_5_} then *M *= {*n*_1_, *n*_3_, *n*_4_} then *M *= {*n*_1_, *n*_3_, *n*_4_, *n*_5_}).

Once a node set *N *has been analysed, re-indexing so that the next node *n*_*j *_to be visited maximises *c *(below) will speed up the search

**- **For the sets of attractors ${\mathbb{C}}_{1},...,{\mathbb{C}}_{k}$ identified in Step 2 (for the new node set *M *= *N *∪ {*n*_*j*_}), ℂ_*i *_= {*A*_*i*_} is a single attractor for *c *(≤ *k*) different values of *i*

Although this involves carrying out Step 2 multiple times (to compare different *n*_*j*_'s), selecting an *n*_*j *_that gives lots of single attractor ℂ_*i*_'s will mean less analysis later on (as discussed above). The quicker we can reach a stage where every set ℂ_*i *_in Step 2 is a single attractor, the more of the tree can be ignored during the rest of the search.

### Algorithms: Partition sequences (Stage 1)

In order to identify every partition sequences, we start with the full set of intersection sequences and all the corresponding sets of attractors (obtained from Procedure 3). i.e. every pair {*P*, ℂ} such that *P *intersects at ℂ. Then, these intersection sequences are used to identify all the partial state sequences that satisfy any of properties **A**, **B **or **C **of Definition 5. Using the complete list of intersection sequences, properties **A**, **B **and **C **are considered independently and partition sequences stored after each test.

#### Part A: Core components

From Procedure 3, we get a set **S **that contains the complete set of intersection sequences, along with the set of attractors each one intersects at (if {*P'*, ℂ} **S**, then *P' *intersects at ℂ).

Using this set **S **as an input, the following procedure identifies every partial state sequence that is core to some set of attractors (i.e every partial state sequence *P *satisfying Definition 5A)

#### Procedure 4. A

Initially, let the set **T **= ∅ (empty set)

Then, for every intersection sequence $P^{'}=\{y_{0}^{N^{\prime }},y_{1}^{N^{\prime }},...,y_{r-1}^{N^{\prime }}\}\in \{P^{'},\mathbb{C}\}\in S$, carry out the following steps

**Step 1: **From the complete set of intersection sequences (**S**), identify every *Q*_*i *_(for the node set *M*_*i*_) for which

**(a) ***Q*_*i *_intersects at D_*i*_, where $D_{i}\cap \mathbb{C}\neq \emptyset$

**(b) **There is no intersection sequence Q* (for a larger node set M* ⊃ *M*_*i*_) that intersects at $D^{*}⊇D_{i}\cap \mathbb{C}$

**Step 2: **Let *k *be the number of partial state sequences from Step 1

**Step 3: **Let *N *= *M*_1 _∩ ... ∩ *M*_*k *_(*N *⊆ *N' *since *P' *is itself identified in Step 1)

**Step 4: **If *N *≠ ∅ in Step 3, find a partial state sequence $P=\{x_{0}^{N},x_{1}^{N},...,x_{q-1}^{N}\}$ that occurs in *P' *(see Procedure 1).

**Step 5: **If *N *≠ ∅ in Step 3, add the pair {*P*, ℂ} to the set **T**

end of procedure

At the end of the procedure, **T **contains every partial state sequence *P *that is core to some set of attractors ℂ (i.e every partial state sequence *P *satisfying Definition 5A). Essentially, we take each intersection sequence *P' *in turn and then re-run though the set of all intersection sequences to find those (*Q*_*i*_, for a node set *M*_*i*_) that satisfy the following

**(a) ***Q*_*i *_co-occurs with *P' *in at least one attractor (*Q*_*i *_can be *P'*).

**(b) ***Q*_*i *_isn't contained in a larger intersection sequence (Q*, say) that co-occurs with *P' *in the same attractors

Then, the node set *N *= *M*_1 _∩ ... ∩ *M*_*k *_is the core set of nodes underlying *P' *and the attractors it occurs in (ℂ). The partial state sequence *P *that satisfies property A is then just partial state sequence (for the node set *N*) that occurs in *P'*

A formal proof for this procedure can be be found in Additional file 1 (see Theorem S1.17 in section S1.2.2). However, here, we give a brief justification.

The 3 properties of Definition 5A are satisfied, for all the identified partial state sequences *P*. Property 2 is satisfied because of the following. If an intersection sequence *Q *(for a node set *M*) intersects at a set of attractors D (where D∩ℂ≠∅), then there is an intersection sequence *Q' *(for a node set *M'*) identified in step 1 for which

- *M *⊆ *M' *⊆ *N *(because of Steps 1 and 3)

- *Q' *occurs in every attractor *A *∈ D∩ℂ (because of Step 1)

and so

- *M *∪ *N *⊇ *M'*

- *Q' *occurs in every attractor *A *∈ D∩ℂ

Property 1 is satisfied because of Step 4. Property 3 is satisfied because of the way *N *is chosen in Step 3. We note that every partial state sequence satisfying Definition 5A is identified because (a) Property 1 implies that such a sequence must occur in an intersection sequence and (b) every intersection sequence is analysed independently.

#### Part B: Exclusive (Procedure 4B)

This is simply done by searching through all intersection sequences (in **S**) and identifying those that satisfy Definition 5B.

#### Part C: Independently Oscillating (Procedure 4C)

This is simply done by searching through every pair of intersection sequences (in **S**) to see which pairs satisfy Definition 5C. Where such a pair is found, both of them are partition sequences.

### Algorithms: Subsystems (Stage 2)

In order to identify every subsystem, we start with the full set of partition sequences from Stage 1. i.e. every partial state sequence satisfying properties either A, B and C of Definition 5, The following procedure identifies every subsystem (satisfying Definition 6)

Procedure 5.

Initially, let the set **U **= ∅ (empty set)

Then, for every partition sequence $P=\{y_{0}^{M},y_{1}^{M},...,y_{r-1}^{M}\}$ (identified in **A**, **B **or **C **of the previous section), carry out the following steps

**Step 1: **From the complete set of partition sequences, identify every partition sequence *P*_*i *_(for a node set *M*_*i*_) for which

**(a) ***M*_*i *_⊂ *M*

**(b) ***P*_*i *_and *P *both co-occur in some attractor *A*

(This also implies that *P*_*i *_occurs in *P*)

**Step 2: **Let *k *be the number of partition sequences from Step 1

**Step 3: **Let *N *= *M*\(*M*_1 _∪ ... ∪ *M*_*k*_)

**Step 4: **If *N *≠ ∅, identify $S=\{x_{0}^{N},x_{1}^{N},...,x_{q-1}^{N}\}$ that occurs in *P *(see Procedure 1)

**Step 5: **If *N *≠ ∅, add *S *(identified in Step 4) to the set **U**

end of procedure

For each partition sequence *P *(for a node set *M*, say), we look to see which other partition sequences ${P^{\prime }}_{1},...,{P^{\prime }}_{k}$ (for node sets ${M^{\prime }}_{1},...,{M^{\prime }}_{k}$) occurs within it. Then the node set *N*, which is unique to *P*, can be calculated as *N *= *M*\(*M*_1 _∪ ... ∪ *M*_*k*_). If *N *≠ ∅, the partial state sequence *S *(for the node set *N*) that occurs in *P*, is stored as a subsystem.

A formal proof for this procedure can be be found in Additional file 1 (see Theorem S1.19 in section S1.3). However, here, we give a brief justification.

At the end of the procedure, **U **contains every subsystem *P *satisfying the 3 properties of Definition 6. Essentially, property 1 is satisfied because of Step 4. Property 2 is satisfied because of Step 1 and the choice of *N *in step 3. Property 3 is satisfied because *N *is the largest set for which *M*_*i *_∩ *N *= ∅ for all *i *= 1, ..., *k*. We note that every partial state sequence satisfying Definition 6 is identified because (a) Property 1 implies that such a sequence must occur in an partition sequence and (b) every partition sequence is analysed independently.

### Algorithms: Regulation of subsystems

In order to show how regulation sets can be identified for each subsystem, we first focus on how partial states are regulated in Boolean network models (although all the methods are applicable to other discrete-state discrete-time logical models).

It may be that a partial state **x**^*N *^controls some Boolean functions {*f*_*i *_: *n*_*i *_∈ *M*} and ensure the occurrence of **y**^*M *^in the following time step. i.e **x**^*N *^is a *predecessor *of **y**^*M *^(or **x**^*N *^*triggers the occurrence *of **y**^*M*^). For example, in Fig. 2, by following the Boolean functions it is possible to say that

- The occurrence of $x_{3}^{N}$ = {*s*_2 _= 1, *s*_3 _= 1} can be triggered by the occurrence of $x_{2}^{N}$ = {*s*_1 _= 1, *s*_2 _= 1}.

- The occurrence of $x_{2}^{N}$ = {*s*_1 _= 1, *s*_2 _= 1} can be triggered by the occurrence of $x_{1}^{N}$ = {*s*_1 _= 1}.

or $x_{1}^{N}$ = {*s*_1 _= 1} *triggers the occurrence of *$x_{3}^{N}$ = {*s*_2 _= 1, *s*_3 _= 1} after two time steps.

This notion of predecessors can be extended to look for predecessors within attractors and subsystems. Going backwards around an attractor it is possible to identify which partial states are responsible for triggering the occurrence of other partial states in the same attractor state at a later point in time. Returning to the example in Fig. 2, and looking at attractor *A*_1_, it is possible to go backwards around the attractor via two different routes to say

Route 1:

The occurrence of {*s*_2 _= 1, *s*_3 _= 1} in **z**_0 _can be triggered by the occurrence of {*s*_1 _= 1, *s*_2 _= 1} in **z**_3_

The occurrence of {*s*_1 _= 1, *s*_2 _= 1} in **z**_1 _can be triggered by the occurrence of {*s*_1 _= 1} in **z**_2_

The occurrence of {*s*_1 _= 1} in **z**_2 _can be triggered by the occurrence of {*s*_1 _= 1} in **z**_1_

The occurrence of {*s*_1 _= 1} in **z**_3 _can be triggered by the occurrence of {*s*_1 _= 1} in **z**_0_

Route 2:

The occurrence of {*s*_2 _= 1, *s*_3 _= 1} in **z**_0 _can be triggered by the occurrence of {*s*_2 _= 1, *s*_3 _= 1} in **z**_3_

The occurrence of {*s*_2 _= 1, *s*_3 _= 1} in **z**_1 _can be triggered by the occurrence of {*s*_2 _= 1, *s*_3 _= 1} in **z**_2_

The occurrence of {*s*_2 _= 1, *s*_3 _= 1} in **z**_2 _can be triggered by the occurrence of {*s*_2 _= 1, *s*_3 _= 1} in **z**_1_

The occurrence of {*s*_2 _= 1, *s*_3 _= 1} in **z**_3 _can be triggered by the occurrence of {*s*_2 _= 1, *s*_3 _= 1} in **z**_0_

and so we have

1. {*s*_1 _= 1} *triggers the occurrence of *{*s*_2 _= 1, *s*_3 _= 1} in the attractor state **z**_0_

2. {*s*_2 _= 1, *s*_3 _= 1} *triggers the occurrence of *{*s*_2 _= 1, *s*_3 _= 1} in the attractor state **z**_0_

(Note: Similar conclusions could also be made for **z**_1_, **z**_1 _and **z**_3_)

So, more generally, given a partial state **y**^*M *^that occurs in an attractor state **z **∈ *A*, we want to be able to find a suitable collection of predecessors $x_{1}^{N_{1}},...,x_{k}^{N_{k}}$ (that *trigger the occurrence of ***y**^*M *^in **z**).

**Procedure 6. **Given a partial state **y**^*M *^that occurs in an attractor state **z **∈ *A*, this procedure identifies partial states $x_{1}^{N_{1}},...,x_{k}^{N_{k}}$ that *trigger the occurrence of ***y**^*M *^in **z**.

Starting from the partial state **y**^*M *^in **z**, this procedure involves going backwards around the attractor *A *(as in the above example) and identifying new predecessors at each step (using a method adapted from [13]). The procedure ends when no more new predecessors $x_{j}^{N_{j}}$ (that trigger the occurrence of **y**^*M *^in **z**) can be found.

Since much of this method involves adapting a previously published algorithm (from [13]), we do not give full details here. More details of this procedure can be found in section S2.1 of Additional file 2 (in particular, Procedures S2.8 and S2.11)

end of procedure

We now want to apply these procedures to subsystems. In particular, we want to find collections of subsystems $S_{x}=\{S_{x_{1}},...,S_{x_{y}}\}$ whose co-occurrence in an attractor *triggers *chain of interactions that results in the occurrence of *S*_*y*_.

Returning to the above example (from Fig. 2), we carry out Procedure 6 on *S*_1 _= {*s*_2 _= 1, *s*_3 _= 1} in all of the attractor states in *A*_1_, *A*_2 _and *A*_3 _(which contain *S*_1_), which gives the following results.

*A*_1 _**and ***A*_2_**: ***S*_1 _= {*s*_2 _= 1, *s*_3 _= 1} can trigger the occurrence of *S*_1 _= {*s*_2 _= 1, *s*_3 _= 1} in the attractor states **z**_0_, **z**_1_, **z**_2 _and **z**_3_

*A*_1 _**and ***A*_2_**: ***S*_2 _= {*s*_1 _= 1} can trigger the occurrence of *S*_1 _= {*s*_2 _= 1, *s*_3 _= 1} in the attractor states **z**_0_, **z**_1_, **z**_2 _and **z**_3_

*A*_3_**: ***S*_1 _= {*s*_2 _= 1, *s*_3 _= 1} can trigger the occurrence of *S*_1 _= {*s*_2 _= 1, *s*_3 _= 1} in the attractor states **z**_0 _and **z**_1_

Therefore, by looking at all of the attractor states (individually) in each attractor, we can say that

**(a) ***S*_2 _can *trigger *the occurrence of *S*_1 _in attractors *A*_1 _and *A*_2_.

**(b) ***S*_1 _can *trigger *the occurrence of itself (*S*_1_) in attractors *A*_1_, *A*_2 _and *A*_3_.

This is then sufficient to give a regulation set for *S*_1_; namely S_1 _= {*S*_1_} and S_2 _= {*S*_2_}. In this very simple example, the same subsystems/partial states are involved in every attractor state within an attractor. However, it may be case that different subsystems are involved in triggering different partial states (from a subsystem *S*_*y*_) in different attractor states, and so it is necessary to consider each attractor state individually.

Procedure 7 (below) demonstrates a method of identifying a *regulation set *for a subsystem $S_{y}=\{y_{0}^{M},...,y_{p-1}^{M}\}$. This is done by looking at every attractor state in every attractor (containing *S*_*y*_) to see how what triggers the occurrence of each relevant partial state $y_{i}^{M}$.

However, since partial states within subsystems could be subject to different time lags in different attractors (see Definition 3), we first note that we need to consider the precise dynamics (the *instances*) of subsystems in attractors. i.e.

**Definition 9. **Consider a collection of subsystems S = {*S*_1_, ..., *S*_*f*_} where every $S_{i}=\{x_{i_{0}}^{N_{i}},...,x_{i_{q_{i}}}^{N_{i}}\}\in S$ involves a node set *N*_*i *_and occurs in the attractor *A *= {**z**_0_, ..., **z**_*p*-1_}

The *instance *of S in *A *is the partial state sequence $z_{0}^{M},...,z_{p-1}^{M}$, where

1. *M *= *N*_1 _∪ ... ∪ *N*_*f*_

2. For *k *= 0, ..., *p *- 1, $z_{k}^{M}$ = {*s*_*x *_∈ **z**_*k *_: *n*_*x *_∈ *M*}.

We can then use this terminology to describe whether a collection of subsystems S = {*S*_1_, ..., *S*_*f*_} *triggers *an individual subsystem *S*_*y *_in an attractor *A*. i.e If the co-occurrence of subsystems *S*_1_, ..., *S*_*f *_ensures the occurrence of *S*_*y *_in *A*.

**Definition 10. **Suppose we have

1. An attractor *A *= {**z**_0_, ..., **z**_*p*-1_}

2. A collection of subsystems $S_{x}=\{S_{x_{1}},...,S_{x_{y}}\}$ where

**(a) **$S_{x_{1}},...,S_{x_{f}}$ all occur in *A*

**(b) **$x_{0}^{N},...,x_{p-1}^{N}$ is the instance of S_*x *_in *A*

3. An individual subsystem *S*_*y *_where

**(a) ***S*_*y *_occurs in *A*

**(b) **$y_{0}^{M},...,y_{p-1}^{M}$ is the instance of *S*_*y *_in *A*

Then S_*x *_*triggers *the occurrence of *S*_*y *_in *A *if the following holds for every *i *∈ {0, ..., *p *- 1}

- $x_{i}^{N}$ triggers the occurrence of $y_{i}^{M}$ in the attractor state **z**_*i *_∈ *A*.

We now give the procedure for identifying a *regulation set *of a subsystem *S*_*y *_(Procedure 7). At the start of the procedure, we take a subsystem *S*_*y *_(involving a node set *M*_*y*_) and a set of attractors ℂ_*y*_, where

**(a) ***S*_*y *_occurs in every attractor *A *∈ ℂ_*y*_

**(b) ***S*_*y *_does not occur in any attractor *A *∉ ℂ_*y*_

Moreover, we assume we know every subsystem $S=\{x_{0}^{N},...,x_{q-1}^{N}\}$, along with the node set involved (*N*) and a list of attractors it occurs in. Each subsystem and node set *N *is a by-product of the method of identifying subsystems (described previously in this *Methods *section). A list of attractors can be found by applying Procedure 2 to the node set *N *(and the full set of attractors). This information is used in Step 2 of the procedure (below).

Procedure 7.

Initially, let the set **R **= ∅ (empty set)

For every *A*_*i *_= {**z**_0_, ..., **z**_*p*-1_} ∈ ℂ_*y *_carry out the following steps.

**Step 1: **Let the set **R**_*i *_= ∅. Let the sets **U**_0_, ..., **U**_*p*-1 _= ∅

**Step 2: **Identify every subsystem *T*_1_, ...., *T*_*h *_that occurs in *A*_*i*_. Moreover, let *M*_1_, ..., *M*_*h *_be the node sets involved in *T*_1_, ...., *T*_*h *_(resp)

**Step 3: **Identify the instance of *S*_*y *_in *A*_*i*_. i.e. $y_{0}^{M_{y}},...,y_{p-1}^{M_{y}}$.

(The procedure for this is obvious from Definition 9, given the node set *M*_*y *_and attractor *A*_*i*_)

**Step 4: **For *j *= 0, ..., *p *- 1, carry out Procedure 6 to identify predecessors of $y_{j}^{M_{y}}$ in **z**_*j *_∈ *A*_*i *_(i.e. the partial states that trigger the occurrence of $y_{j}^{M_{y}}$ in **z**_*j*_). The resulting predecessors are added to the set **U**_*j*_

**Step 5: **For every possible combination of partial states $x_{0}^{N_{0}},...,x_{p-1}^{N_{p-1}}$ satisfying

- $x_{j}^{N_{j}}$ ∈ **U**_*j *_(for *j *= 0, ..., *p *- 1)

carry out the following

**(a) **Let *N *= *N*_0 _∪ ... ∪ *N*_*p*-1_

**(b) **Let S = {*T*_*a *_: *M*_*a *_∩ *N *≠ ∅}

**(c) **Add S to the set **R**_*i*_

**Step 6: **Remove all subsystem collections S from **R**_*i *_that contain other subsystem collections S' ∈ **R**_*i*_. (i.e. $S⊃S^{\prime }$)

**Step 7: **Add the subsystem collections in **R**_*i *_to the set **R**

end of procedure

At the end of this procedure, every subsystem collection S ∈ **R**_*i *_triggers the occurrence of *S*_*y *_in the attractor *A*_*i *_(i.e. Definition 10 is satisfied). For every attractor state **z**_*j *_∈ *A*_*i*_, we identify the partial state $y_{j}^{M_{y}}$ ∈ *S*_*y *_that occurs in **z**_*j *_(Step 3). Then, starting from the partial state $y_{j}^{M_{y}}$ in the attractor state **z**_*j*_, we go backwards around the attractor, identifying suitable predecessors at each time step (this backwards process can go multiple times around the attractor). This will then give us a list of partial states that can trigger the occurrence of $y_{j}^{M_{y}}$ in **z**_*j *_(added to **U**_*j *_Step 4). Having done this for every attractor state **z**_*j *_∈ *A*_*i*_, Step 5 then pulls the results together to find collections of subsystems S = {*S*_1_, ..., *S*_*f*_} that *triggers *the occurrence of *S*_*y *_in *A *(i.e. those collections that satisfy Definition 10). In particular, finding collections for which the following is true.

For every attractor state **z**_*j *_∈ *A*_*i*_, there exists a partial state $x_{j}^{N_{j}}$ that satisfies

**(a) **$x_{j}^{N_{j}}$ triggers the occurrence of $y_{j}^{M_{y}}$ in **z**_*j*_

**(b) **$x_{j}^{N_{j}}$ only involves nodes and states from the subsystems {*S*_1_, ..., *S*_*f*_}

Step 6 just ensures that only the most informative collections are kept and there is no redundancy. Finally, since these collections of subsystems are added to the set **R **in Step 7, for every attractor (which contains *S*_*y*_), **R **= {$S_{1},...,S_{g}$} is a regulation set and satisfies the properties of Definition 7.

A formal proof for this procedure can be be found in Additional file 2 (see Theorem S2.20 in section S2.2.2). However, this procedure shows just one way of a regulation set, for a subsystem *S*_*y*_. There may be more than one possible regulation set for a subsystem and some may be more descriptive than others. In Section S2.2.1 and S2.2.2 of Additional file 2, we give some some extra constraints (and corresponding procedures) that can be applied when looking for regulation sets.

## Authors' contributions

DI designed the method of identifying subsystems, co-conceived the study and drafted the manuscript. NM co-conceived the study and drafted the manuscript. All authors read and approved the final manuscript.

## Supplementary Material

Additional file 1 Description of the procedures used to identify subsystems, along with formal proofs. This is given as a pdf file and is referred to in the main text. Click here for file

Additional file 2 Description of the procedures used to identify interactions between subsystems, along with formal proofs. This is given as a pdf file and is referred to in the main text. Click here for file

Additional file 3 Extra examples associated with the method of identifying subsystems. This is given as a pdf file. Click here for file

Additional file 4 Extra information associated with the model/analysis of the segment polarity network. This is given as a pdf file and is referred to in the main text. Click here for file
