# Supplementary material for: Identifying dynamical modules from genetic regulatory systems: applications to the segment polarity network
Source: BMC Bioinformatics. 2007 Oct 25;8:413. doi: 10.1186/1471-2105-8-413 (PMC2233651; doi:10.1186/1471-2105-8-413)
Supplement: Additional file 1 — Description of the procedures used to identify subsystems, along with formal proofs. This is given as a pdf file and is referred to in the main text. [file 1471-2105-8-413-S1.pdf]

Supporting Text 1:
Procedure to identify every subsystem

Here, we describe a 2-stage method for identifing every subsystem in a Boolean network model. As an input for the method, we only require a set attractors $\mathbb{A} = \{A_1, ..., A_r\}$, where each attractor $A = \{\mathbf{z}_0, ..., \mathbf{z}_{p-1}\} \in \mathbb{A}$ is a (repeating) series of discrete states for the whole network / system. Therefore, the same method can be applied to any set of discrete state, discrete time attractors (without a Boolean network model). *Supporting Text 3* also provides some additional examples that demonstrate the main features of the method (in a less formal manner).

The two stages are described in Sections S1.2 and S1.3 below, which involve identifying every partial state sequence P that satisfies Definitions 5 and 6 in the main text. Section S1.2 describes how to identify every *partition sequence* (satisfying Definition 5). These provide a hierarchical breakdown of the attractors and are used as the basis for identifying every *subsystems* (satisfying Definition 6) in Section S1.3. In both of these stages, we look for partial state sequences that *occur* in attractors (i.e. satisfy Definition 3 of the main text). Therefore, in Section S1.1, we first demonstrate how to identify partial state sequences that *occur* in attractors or *occur* in larger partial state sequences.

This supporting text is a more formal description / proof of the procedures given in the main manuscript. The procedures in the main manuscript are revisited here in the following sections.

Procedure 1: Corresponds to Procedure S1.3 and Theorem S1.4 in Section S1.1.1

Procedure 2: Corresponds to Procedure S1.9 and Theorem S1.10 in Section S1.1.3

Procedure 3: Corresponds to Procedure S1.13 and Theorem S1.14 in Section S1.2.1

Procedure 4: Corresponds to Procedure S1.16 and Theorem S1.17 in Section S1.2.2

Procedure 5: Corresponds to Procedure S1.18 and Theorem S1.19 in Section S1.3

One thing to note first, is that if a partial state sequence $P = \{\mathbf{x}_0^N, \mathbf{x}_1^N, ..., \mathbf{x}_{q-1}^N\}$ occurs in an attractor A, so will $q-1$ other *equivalent* partial state sequences $P' = \{\mathbf{y}_0^N, \mathbf{y}_1^N, ..., \mathbf{y}_{q-1}^N\}$ where the partial states in P have been rotated modulo q. This is since rotating the partial states in P (modulo q) will leave a partial state sequence that still satisfies the 3 properties of Definition 3. The following Definition and Theorem formalise this.

Definition S1.1. A partial state sequences $P_x = \{\mathbf{x}_0^N, \mathbf{x}_1^N, ..., \mathbf{x}_{q-1}^N\}$ is *equivalent* to another partial state sequence $P_w = \{\mathbf{w}_0^M, \mathbf{w}_1^M, ..., \mathbf{w}_{r-1}^M\}$ if the following hold

1. $M = N$
2. $q = r$
3. $\exists$ an integer c such that, $\mathbf{x}_i^N = \mathbf{w}_j^M$, for all $i \in \{0, 1, ..., q-1\}$ and $j = i + c \pmod q$

Whenever P_x is equivalent to P_w, P_w is also equivalent to P_x (replace c by $-c \pmod q$) in the above definition)

Theorem S1.2. Consider a partial state sequence $P_x = \{\mathbf{x}_0^N, ..., \mathbf{x}_{q-1}^N\}$ that occurs in an attractor $A = \{\mathbf{z}_0, ..., \mathbf{z}_{p-1}\}$

A partial state sequence $P_w = \{\mathbf{w}_0^N, ..., \mathbf{w}_{r-1}^N\}$ (for the same node set N) occurs in $A \iff P_w$ is equivalent to P_x

PROOF: see Section S1.4.1

From now on, we primarily consider only one of the the q equivalent partial states sequences, since if one occurs in an attractor, so will the other $q - 1$.

S1.1 Preliminary results and procedures

Given a node set N and attractor $A = \{\mathbf{z}_0, \mathbf{z}_1, ..., \mathbf{z}_{p-1}\}$, Section S1.1.1 provides a procedure to identify a partial state sequence $P = \{\mathbf{x}_0^N, \mathbf{x}_1^N, ..., \mathbf{x}_{q-1}^N\}$ that occurs in A (i.e. the properties of Definition 3 are satisfied). Section S1.1.2 adapts this procedure to look for partial state sequences (for a node set N) occurring in other partial state sequences (for a larger node set $M \supset N$).

Section S1.1.3 then extends these ideas to sets of attractors $\mathbb{C}$, to identify partial state sequences P_1, ..., P_k (for a node set N) that partition $\mathbb{C}$ into distinguishable groups.

S1.1.1 Partial state sequences occurring in an attractor A

Given a node set N and an attractor $A = \{\mathbf{z}_0, \mathbf{z}_1, ..., \mathbf{z}_{p-1}\}$, the following procedure identifies a partial state sequence $P = \{\mathbf{x}_0^N, ..., \mathbf{x}_{q-1}^N\}$ that occurs in A (i.e. the 3 properties of Definition 3 of the main text are satisfied)

Procedure S1.3. (Procedure 1 from main manuscript)

Initially let $k = 0$, $b_0 = 0$ and $\mathbf{x}_0^N = \{s_i \in \mathbf{z}_0 : n_i \in N\}$. The enter the following loop

Step 1
If $k = p - 1$, let $q^* = b_{p-1} + 1$ and **go to** step 6

Step 2
Let $j = k$ and increment k by 1 (let $k = k + 1$)

Step 3
If $\mathbf{x}_{b_j}^N = \{s_i \in \mathbf{z}_k : n_i \in N\}$, then let $b_k = b_j$ and **go to** step 1
(otherwise **go to** step 4)

Step 4
Let $b_k = b_j + 1$

Step 5
Let $\mathbf{x}_{b_k}^N = \{s_i \in \mathbf{z}_k : n_i \in N\}$ and **go to** step 1

Step 6
If $\mathbf{x}_{b_{p-1}}^N = \mathbf{x}_{b_0}^N$ **and** $q^* > 1$, reduce q^* by 1 (let $q^* = q^*$ - 1)

Step 7
Let q be the smallest integer for which both

(a) $q \mid q^*$ (this can be $q = q^*$)

(b) $\mathbf{x}_f^N = \mathbf{x}_g^N$, whenever $f \leq b_{p-1}$, $g \leq b_{p-1}$ and $f \pmod q = g \pmod q$

Step 8
For $k = 0, ..., p-1$, let $b_k = b_k \pmod q$

At the end of this procedure $P = \{\mathbf{x}_0^N, ..., \mathbf{x}_{q-1}^N\}$ occurs in A and the 3 properties of Definition 3 are satisfied. Steps 2-5 ensure that properties 1 and 2 are satisfied and the partial states in P cycle within the attractor A in the correct order (as the attractor progresses over time). Steps 3, 6, 7 and 8 ensures property 3, so that P is smallest possible set of partial states that cycles within A. In particular (a) no two adjacent partial states in P are identical, (b) there are no redundant partial states in P and (c) if a sequence of states cycles many times within an attractor, only one copy is kept. This leaves a partial state sequence that just describes the ′order′ in which the node states change in A (for nodes in N).

We demonstrate this formally with Theorem S1.4 (below) which is proved at the end of this supporting text (Section S1.4.1). In **C**, **C**1 and **C**2 correspond to properties 1 and 2 of Definition 3. **C**3, **C**4 and **C**5 correspond to (a), (b) and (c) discussed above.

Theorem S1.4. Consider a node set N, partial state sequence $P = \{\mathbf{x}_0^N, ..., \mathbf{x}_{q-1}^N\}$ and attractor $A = \{\mathbf{z}_0, \mathbf{z}_1, ..., \mathbf{z}_{p-1}\}$.

If any of one the following 3 statements (**A**, **B** or **C**) is true, they are all true. (i.e. **A** $\Longleftrightarrow$ **B** $\Longleftrightarrow$ **C** $\Longleftrightarrow$ **A**)

A: P occurs in A

B: Given N and A, Procedure S1.3 identifies a partial state sequence P^* that is equivalent to P (this could be $P^* = P$)

C: The following are true for $P = \{\mathbf{x}_0^N, ..., \mathbf{x}_{q-1}^N\}$ and some sequence of integers $b_0, ..., b_{p-1} \in \{0, ..., q-1\}$

1. For $k = 0, ..., p-1$, $\mathbf{x}_{b_k}^N = \{s_i \in \mathbf{z}_k : n_i \in N\}$
2. For each $k \in \{0, ..., p-1\}$ and $j = k-1 \pmod p$, either **(a)** $b_k = b_j$ or **(b)** $b_k = b_j + 1 \pmod q$
3. Given $k \in \{0, ..., p-1\}$ and $j = k-1 \pmod p$, **if** $b_k \neq b_j$ **then** $\mathbf{x}_{b_k}^N \neq \mathbf{x}_{b_j}^N$
4. For each $a \in \{0, ..., q-1\}$, $\exists$ $k \in \{0, ..., p-1\}$ such that $b_k = a$
5. There is no integer $q' \mid q$ $(q' < q)$ for which $\mathbf{x}_f^N = \mathbf{x}_g^N$ whenever f, g satisfies $f \pmod{q'} = g \pmod{q'}$

PROOF: See Section S1.4.1

S1.1.2 Partial state sequences occurring in other partial state sequences

Definition 3 of the main text involves a partial state sequence $P = \{\mathbf{x}_0^N, \mathbf{x}_1^N, ..., \mathbf{x}_{q-1}^N\}$ occurring in an attractor A. However, it is possible to adapt this definition to look at a partial state sequence P_x (for a node set N) occurring in another partial state sequence P_y (for a larger node set $M \supseteq N$). i.e.

Definition S1.5. A partial state sequence $P_x = \{\mathbf{x}_0^N, \mathbf{x}_1^N, ..., \mathbf{x}_{q-1}^N\}$ *occurs* in another partial state sequence $P_y = \{\mathbf{y}_0^M, ...,\mathbf{y}_{r-1}^M\}$ (where $M \supseteq N$) if there exists integers b_0, ..., $b_{r-1} \in \{0, ..., q-1\}$ for which the following is true

1. For $k = 0, ..., r-1$, $\quad \mathbf{x}_{b_k}^N = \{s_i \in \mathbf{y}_k^M \ : \ n_i \in N\}$
2. For each $k \in \{0, ..., r-1\}$ and $j = k-1 \pmod r$, either
   (a) $b_k = b_j$ or **(b)** $b_k = b_j + 1 \pmod q$
3. 1 and 2 are not true for any smaller partial state sequence $P' = \{\mathbf{z}_0^N, \mathbf{z}_1^N, ..., \mathbf{z}_{q'-1}^N\}$ and integers c_0, ..., $c_{r-1} \in \{0, ..., q'-1\}$ $\quad (q' < q)$

Given a node set $N \subseteq M$ and a partial state sequence $P_y = \{\mathbf{y}_0^M, \mathbf{y}_1^M, ..., \mathbf{y}_{r-1}^M\}$, Procedure S1.3 can be adapted to identify a partial state sequence $P_x = \{\mathbf{x}_0^N, \mathbf{x}_1^N, ..., \mathbf{x}_{q-1}^N\}$, that occurs in P_y.

Procedure S1.6. Initially let $k = 0$, $b_0 = 0$ and $\mathbf{x}_0^N = \{s_i \in \mathbf{y}_0^M \ : \ n_i \in M\}$. The enter the following loop.

Then enter the same loop as Procedure S1.3, except replace p with r (in Step 1) and replace $\mathbf{z}_k$ with $\mathbf{y}_k^M$ (in Steps 3 and 5)

Theorems S1.2 and S1.4 can also be adapted and proved in an analogous manner, replacing p with r and replacing $A = \{\mathbf{z}_0, ..., \mathbf{z}_{p-1}\}$ with $P_y = \{\mathbf{y}_0^M, ..., \mathbf{y}_{r'-1}^M\}$

Theorem S1.7. Consider a partial state sequence $P_x = \{\mathbf{x}_0^N, ..., \mathbf{x}_{q-1}^N\}$ that occurs in another partial state sequence $P_y = \{\mathbf{y}_0^M, ..., \mathbf{y}_{r-1}^M\}$ $(M \supset N)$

A partial state sequence $P_w = \{\mathbf{w}_0^N, ..., \mathbf{w}_{r-1}^N\}$ (for the same node set N) occurs in $P_y \iff P_w$ is equivalent to P_x

PROOF: Analogous to the proof of S1.2

Theorem S1.8. Consider a node set N and two partial state sequence $P_x = \{\mathbf{x}_0^N, ..., \mathbf{x}_{q-1}^N\}$ and attractor $P_y = \{\mathbf{y}_0^M, ..., \mathbf{y}_{r-1}^M\}$ $(M \supset N)$

P_x occurs in P_y
$\iff$
Given N and P_y, Procedure S1.6 identifies a partial state sequence P^* that is equivalent to P_x (this could be $P^* = P_x$)

PROOF: Analogous to the proof of S1.4

S1.1.3 Partial state sequences partitioning a set of attractors $\mathbb{C}$

Given a node set N and a set of attractors $\mathbb{C}$, we want to find a set of partial state sequences P_1, ..., P_k that are all distinguishable from one another and optimally partition $\mathbb{C}$ into smaller sets $\mathbb{C}_1$, ..., $\mathbb{C}_k$.

One such way is to apply the following procedure (Procedure S1.9). This will identify partial state sequences P_1, ..., P_k and sets of attractors $\mathbb{C}_1$, ..., $\mathbb{C}_k$ that satisfy properties **A- F** in Theorem S1.10

Procedure S1.9. (Procedure 2 from main manuscript)

Begin with the node set N and set of attractors $\mathbb{C}$ and then carry out the following steps

Step 1
For every attractor $A_j \in \mathbb{C}$, apply Procedure S1.3 to N and A_j, to get a partial state sequence Q_j that occurs in A_j.

Step 2
Put the Q_j's into groups $i = 1, ..., k$, whereby two partial state sequences Q'_x, Q'_y go in the same group $\iff Q'_x$ is equivalent to Q'_y.

(here k is the minimum number of groups required to hold every Q_j)

Step 3
For each group, i, let

i) P_i = any Q_j in the group i

ii) $\mathbb{C}_i = \{A_j : Q_j$ is part of the group $i\}$

Theorem S1.10. Given a node set N and set of attractors $\mathbb{C}$, Procedure S1.9 identifies partial state sequences P_1, ..., P_k and sets of attractors $\mathbb{C}_1$, ..., $\mathbb{C}_k$ satisfying

A: For $i = 1, ..., k$, P_i involves the node set N (i.e. $P_i = \{\mathbf{x}^N_{i_0}, ..., \mathbf{x}^N_{i_{q-1}}\}$)

B: For $i = 1, ..., k$, P_i occurs in every attractor $A \in \mathbb{C}_i$

C: For $i = 1, ..., k$, P_i does not occur in any attractor $A \notin \mathbb{C}_i$

D: For any i, j $(1 \leq i < j \leq k)$, $\mathbb{C}_i \cap \mathbb{C}_j = \varnothing$

E: $\mathbb{C}_1 \cup ... \cup \mathbb{C}_k = \mathbb{C}$

F: Given the node set N, there are no other partial state sequences $P' \notin \{P_1, ..., P_k\}$ that occur in any attractor $A \in \mathbb{C}$ (unless P' is equivalent to some $P_i \in \{P_1, ..., P_k\}$)

PROOF See Section S1.4.1

The following results (Lemmas S1.11 and S1.12, below), demonstrate that when a partial state sequence P_x (for a node set N) is extended with extra nodes, the new partial state sequence P_y (for a node set $M \supset N$) can only occur in a smaller subset of attractors.

Lemma S1.11. Consider two partial state sequences $P_x = \{\mathbf{x}_0^N, ..., \mathbf{x}_{q-1}^N\}$ and $P_y = \{\mathbf{y}_0^M, ..., \mathbf{y}_{r-1}^M\}$ (where $M \supseteq N$)

Then,

(a) P_x occurs in P_y, and P_y occurs in an attractor $A \implies P_x$ occurs in A

(b) P_x and P_y both occur in an attractor $A \implies P_x$ occurs in P_y

PROOF: See Section S1.4.1

Lemma S1.12. Consider two partial state sequences $P_x = \{\mathbf{x}_0^N, ..., \mathbf{x}_{q-1}^N\}$ and $P_y = \{\mathbf{y}_0^M, ..., \mathbf{y}_{r-1}^M\}$, and two sets of attractors $\mathbb{C}_x$ and $\mathbb{C}_y$ for which

1. $M \supseteq N$
2. P_x occurs in every attractor $A \in \mathbb{C}_x$
3. P_x does not occur in any attractor $A \notin \mathbb{C}_x$
4. P_y occurs in every attractor $A \in \mathbb{C}_y$
5. P_y does not occur in any attractor $A \notin \mathbb{C}_y$

Then, either

(a) $\mathbb{C}_x \cap \mathbb{C}_y = \varnothing$

(b) P_x occurs in P_y **and** $\mathbb{C}_y \subseteq \mathbb{C}_x$

PROOF: See Section S1.4.1

S1.2 Stage 1: Identifying every partition sequence

In this stage, we first identify every partial every partial state sequence that satisfies Definition 4 of the main text (*intersection sequences*). These are then used to identify every *partition sequence* satisfying Definition 5 of the main text. Therefore, we describe the two procedures separately (in Sections S1.2.1 and S1.2.2 below)

S1.2.1 Definition 4: Intersection sequences

Identifying every intersection sequence is equivalent to finding every partial state sequence that satisfies the 3 properties of Definition 4 (for some set of attractors $\mathbb{C}$, say). I first give a procedure for identifying every intersection sequence, and then discuss ways to make the process more efficient.

Procedure S1.13. (Procedure 3 from main manuscript)

First, consider the tree in Fig.S1.1 and note that for every node set N, there exists a path from left to right (starting at $'-'$) that corresponds to it. Therefore, searching through a tree analogous to the one in Fig.S1.1 (for a network with nodes $V = \{n_1, ..., n_v\}$), every node set N can be visited at some point.

The procedure searches through the tree (as in Fig.S1.1) and carries out the following steps for each node set N. At the end of the procedure the set $\mathbf{S}$ contains every intersection sequence

Step 0 : Initialise
Let $\mathbf{S} = \varnothing$ and let $N = \varnothing$ $(-)$

Step 1 :
Move onto the next node set N in the tree (as in Fig.S1.1).

Step 2 :
For the node set N, apply Procedure S1.9 to identify partial state sequences P_1, ..., P_k and sets of attractors $\mathbb{C}_1$, ..., $\mathbb{C}_k$ satisfying (from Theorem S1.10)

(a) For $i = 1, ..., k$, P_i involves the node set N (i.e. $P_i = \{\mathbf{x}_{i_0}^N, ..., \mathbf{x}_{i_{q-1}}^N\}$)

(b) For $i = 1, ..., k$, P_i occurs in every attractor $A \in \mathbb{C}_i$

(c) For $i = 1, ..., k$, P_i does not occur in any attractor $A \notin \mathbb{C}_i$

(d) For any i, j $(1 \leq i < j \leq k)$, $\mathbb{C}_i \cap \mathbb{C}_j = \varnothing$

(e) $\mathbb{C}_1 \cup ... \cup \mathbb{C}_k = \mathbb{A}$ (the set of all attractors)

(f) Given the node set N, there are no other partial state sequences $P' \notin \{P_1, ..., P_k\}$ that occur in any attractor $A \in \mathbb{A}$ (unless P' is equivalent to some $P_i \in \{P_1, ..., P_k\}$)

Step 3 :
For $i = 1, ..., k$, add the pair $\{P_i, \mathbb{C}_i\}$ to the set $\mathbf{S}$

Step 4 :
For $i = 1, ..., k$, check $\mathbf{S}$ to see if there is any pair $\{Q = \{\mathbf{y}_0^M, ..., \mathbf{y}_{r-1}^M\}, \mathbb{D}\}$ for which either of the following are true

(a) $M \subset N$ and $\mathbb{D} = \mathbb{C}_i$

(b) $M \supset N$ and $\mathbb{D} = \mathbb{C}_i$

If (a) is true, remove $\{Q,\mathbb{D}\}$ from $\mathbf{S}$. If (b) is true, remove $\{P_i, \mathbb{C}_i\}$ from $\mathbf{S}$

Step 5 :
If the tree has been completely searched, **end procedure**. Otherwise, return to step 1.

At the end of the procedure, $\mathbf{S}$ gives a complete set of intersection sequences (satisfying the 3 properties of Definition 4). Step 2 ensures every partial state sequence that satisfies properties 1 and 2 are identified for each node set N. Step 4 then ensures that only those satisfying Step 3 remain in $\mathbf{S}$. We demonstrate this formally with Theorem S1.14 (below), which is proved in Section S1.4.2

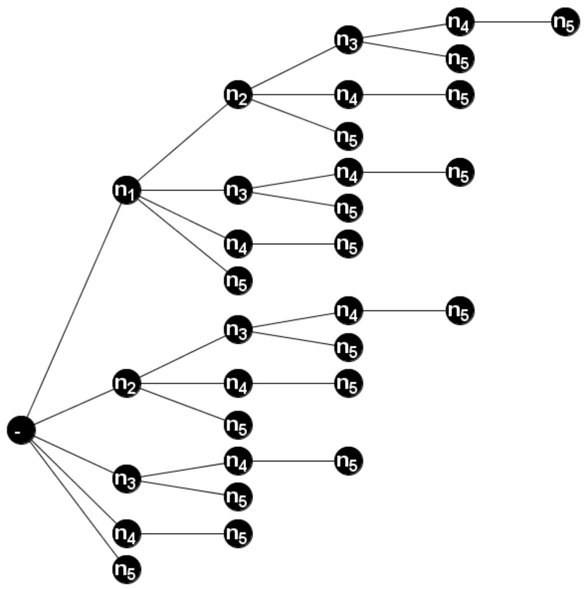

Figure S1.1: Every path from left to right (starting at '-') in this tree represents a different node set $N \subseteq V = \{n_1, n_2, n_3, n_4, n_5\}$. It is possible to search this tree and visit every node set $N \subseteq V$ (exactly once). For example, follow the path $\{n_1\} \rightarrow \{n_1, n_2\} \rightarrow \{n_1, n_2, n_3\} \rightarrow \{n_1, n_2, n_3, n_4\} \rightarrow \{n_1, n_2, n_3, n_4, n_5\} \rightarrow \{n_1, n_2, n_3, n_5\} \rightarrow \{n_1, n_2, n_4\} \rightarrow \{n_1, n_2, n_4, n_5\} \rightarrow \{n_1, n_2, n_5\} \rightarrow \{n_1, n_3\} \rightarrow \{n_1, n_3, n_4\} \rightarrow \{n_1, n_3, n_4, n_5\} \rightarrow \{n_1, n_3, n_5\} \rightarrow \{n_1, n_4\} \rightarrow \{n_1, n_4, n_5\} \rightarrow \{n_1, n_5\} \rightarrow \{n_2\} \rightarrow \{n_2, n_3\} \rightarrow \{n_2, n_3, n_4\} \rightarrow \{n_2, n_3, n_4, n_5\} \rightarrow \{n_2, n_3, n_5\} \rightarrow \{n_2, n_4\} \rightarrow \{n_2, n_4, n_5\} \rightarrow \{n_2, n_5\} \rightarrow \{n_3\} \rightarrow \{n_3, n_4\} \rightarrow \{n_3, n_4, n_5\} \rightarrow \{n_3, n_5\} \rightarrow \{n_4\} \rightarrow \{n_4, n_5\} \rightarrow \{n_5\}$

Theorem S1.14. At the end of Procedure S1.13, the following is true

P is an intersection sequence

$\Longleftrightarrow$

P is equivalent to a partial state sequence $P^* \in \{P^*, \mathbb{C}\} \in \mathbf{S}$

PROOF: See Section S1.4.2

We now explain how the the procedure can be made more efficient.

Improving efficiency(1)

In step 2, partial state sequences P_1, ..., P_k and sets of attractors $\mathbb{C}_1$, ..., $\mathbb{C}_k$ are identified that satisfy (a) to (f) (given a node set N). Now, because of the following Theorem (Theorem S1.15),

If, for a node set N**:** $\mathbb{C}_x \in \mathbb{C}_1$, ..., $\mathbb{C}_k$ is a single attractor after Step 2 (i.e. $\mathbb{C}_x = \{A_x\}$)

Then, for any node set $M \supset N$**:** Step 2 returns the same single attractor $\mathbb{C}_x = \{A_x\}$

This implies that this attractor (A_x) need not be re-analysed during the analysis of any node set $M \supset N$. However, because of part (b) of the Theorem, the full node set $V = \{n_1, ..., n_v\}$ should still be fully analysed in Steps 2 - 4 (possibly at the very end of the procedure).

Theorem S1.15. Consider a partial state sequence $P = \{\mathbf{x}_0^N, ..., \mathbf{x}_{q-1}^N\}$ for which

1. P occurs in a single attractor A
2. P does not occur in any attractor $A' \neq A$

Then, given that V is the set of all nodes,

(a) Given a node set M satisfying $N \subset M \subset V$, it is impossible to find an intersection sequence that involves the node set M and occurs in A.

(b) Given the node set V, it is possible to find an intersection sequence P' that involves the node set V and occurs in A (usually A itself).

PROOF: See Section S1.4.2

We show one way in which this knowledge can improve efficiency in Procedure S1.13. Suppose, attractors A_1', ..., A_f' were returned as single attractors in Step 2, when analysing earlier node sets $P \subseteq N$ (including $P = N$). Then, when a path is extended to the right in the tree (Fig.S1.1) from a node set N to a node set $M \supset N$, Procedure S1.9 in Step 2 need only be applied to the set of attractors

$$\mathbb{A}' = \mathbb{A} \setminus \{A_1', ..., A_f'\}$$

Moreover, if **every** attractor is returned as a single attractor in Step 2, when analysing earlier node sets $P \subseteq N$ (including $P = N$), there is no need to extend the path to look at node sets $M \supset N$. In Fig.S1.1, this is equivalent to ignoring all longer paths that include extra nodes to the right. For example, if $N = \{n_1, n_3\}$, there would be no need to look at longer paths (from left to right) that give node sets $M = \{n_1, n_3, n_4\}$, $M = \{n_1, n_3, n_5\}$ or $M = \{n_1, n_3, n_4, n_5\}$

Improving efficiency(2)

As can be seen in Fig.S1.1, some nodes appear less than others, with the least frequent nodes visited earlier in the tree. Therefore, it is likely to be advantageous to re-index nodes in the tree during the search. At any stage during the search, nodes along paths to the right (from a node set N) can be re-indexed without impairing our ability to search the tree. For example, once $N = \{n_1, n_3\}$ has been reached, re-indexing nodes $\{n_4, n_5\}$ to $\{n_5, n_4\}$ still allows us to reach the same node sets $M = \{n_1, n_3, n_4\}$, $M = \{n_1, n_3, n_5\}$ and $M = \{n_1, n_3, n_4, n_5\}$, as before. However, they would be visited in a different order ($M = \{n_1, n_3, n_5\}$ then $M = \{n_1, n_3, n_4\}$ then $M = \{n_1, n_3, n_4, n_5\}$).

Once a node set N has been analysed, re-indexing so that the next node n_j to be visited maximises c (below) will speed up the search

- For the sets of attractors $\mathbb{C}_1$, ..., $\mathbb{C}_k$ identified in Step 2 (for the new node set $M = N \cup \{n_j\}$), $\mathbb{C}_i = \{A_i\}$ is a single attractor for c ($\leq k$) different values of i

Although this involves carrying out Step 2 multiple times (to compare different n_j's), selecting an n_j that gives lots of single attractor $\mathbb{C}_i$'s will mean less analysis later on (as discussed above).

The quicker we can reach a stage where every set $\mathbb{C}_i$ in Step 2 is a single attractor, the more of the tree can be ignored during the search.

S1.2.2 Definition 5: Partition sequences

In order to find every partition sequence satisfying Definition 5, it is necessary to find every partial state sequence $P = \{\mathbf{x}_0^N, \mathbf{x}_1^N, ..., \mathbf{x}_{q-1}^N\}$ that satisfies any of the following properties (**A**, **B** or **C**), for some set of attractors $\mathbb{C}$

A : P is Core to $\mathbb{C}$

The following 3 properties hold for P

1. P occurs in an intersection sequence P', which intersects at $\mathbb{C}$ (P can equal P').
2. If an intersection sequence Q (for a node set M) intersects at $\mathbb{D}$ (where $\mathbb{D} \cap \mathbb{C} \neq \varnothing$), then there exists an intersection sequence Q' (for a node set $M' \supseteq M \cup N$) that occurs in every attractor $A \in \mathbb{D} \cap \mathbb{C}$
3. 1 and 2 are not true for any larger partial state sequence P'' (for a node set $N'' \supset N$)

B : P is Exclusive to $\mathbb{C}$

P is the only intersection sequence that intersects at $\mathbb{C}$
(excluding those partial state sequences that are equivalent to P)

C : P is Independently Oscillating

P intersects at $\mathbb{C}$ and cycles out of phase with another intersection sequence Q. i.e. $\exists\, Q$ that involves the node set M and intersects at $\mathbb{D}$, for which

1. $|\ \mathbb{C} \cap \mathbb{D}\ | \geq 2$
2. $N \cup M = V$ (the set of all nodes)

Using the complete list of intersection sequences (see above)), these can be found as follows

Part A: Core components

From Procedure S1.13, we get a set $\mathbf{S}$ that contains the complete set of intersection sequences, along with the set of attractors each one intersects at (if $\{P', \mathbb{C}\} \in \mathbf{S}$, then P' intersects at $\mathbb{C}$).

Using this set $\mathbf{S}$ as an input, the following procedure identifies every partial state sequence that is core to some set of attractors (i.e every partial state sequence P satisfying Definition 5A)

Procedure S1.16. (Procedure 4 from main manuscript)

Initially, let the set $\mathbf{T} = \varnothing$ (empty set). Then, for every intersection sequence $P' = \{\mathbf{y}_0^{N'}, \mathbf{y}_1^{N'},$ ..., $\mathbf{y}_{r-1}^{N'}\} \in \{P',\mathbb{C}\} \in \mathbf{S}$, carry out the following steps

Step 1:
From the complete set of intersection sequences ($\mathbf{S}$), identify every Q_i (for the node set M_i) for which

(a) Q_i intersects at $\mathbb{D}_i$, where $\mathbb{D}_i \cap \mathbb{C} \neq \varnothing$

(b) There is no intersection sequence Q^* (for a larger node set $M^* \supset M_i$) that intersects at $\mathbb{D}^* \supseteq \mathbb{D}_i \cap \mathbb{C}$

Step 2:
Let k be the number of partial state sequences from Step 1

Step 3:
Let $N = M_1 \cap ... \cap M_k$ ($N \subseteq N'$ since P' is itself identified in Step 1)

Step 4:
If $N = \varnothing$ in Step 3, apply Procedure S1.6 to find a partial state sequence $P = \{\mathbf{x}_0^N, \mathbf{x}_1^N, ..., \mathbf{x}_{q-1}^N\}$ that occurs in P'.

Step 5:
If $N = \varnothing$ in Step 3, add the pair $\{P,\mathbb{C}\}$ to the set $\mathbf{T}$

At the end of the procedure, $\mathbf{T}$ contains every partial state sequence P that is core to some set of attractors $\mathbb{C}$ (i.e every partial state sequence P satisfying Definition 5A). Essentially, in each loop, N is the set of nodes that is core to every intersection sequence that occurs in any attractor $A \in \mathbb{C}$. We demonstrate this formally with Theorem S1.17 (below), which is proved in Section S1.4.2

Theorem S1.17. At the end of Procedure S1.16, the following is true

P that is core to some set of attractors $\mathbb{C}$ (Definition 5A is satisfied)
$\Longleftrightarrow$
P is equivalent to a partial state sequence $P^* \in \{P^*,\mathbb{C}\} \in \mathbf{T}$

PROOF: See Section S1.4.2

Part B: Exclusive

This is simply done by searching through all intersection sequences (in $\mathbf{S}$) and identifying those that satisfy Definition 5B.

Part C: Independently Oscillating

This is simply done by searching through every pair of intersection sequences (in $\mathbf{S}$) to see which pairs satisfy Definition 5C. Where such a pair is found, both of them are partition sequences.

S1.3 Stage 2: Subsystems

Using all the partition sequences from Stage 1 as an input (i.e those partial state sequences identified in A,B and C of Section S1.2.2), the following procedure identifies every subsystem (satisfying Definition 6 of the main text)

Procedure S1.18. (Procedure 5 from main manuscript)

Initially, let the set $\mathbf{U} = \varnothing$ (empty set)

Then, for every partition sequence $P = \{\mathbf{y}_0^M, \mathbf{y}_1^M, ..., \mathbf{y}_{r-1}^M\}$ (identified in **A**, **B** or **C** of Section S1.2.2), carry out the following steps

Step 1:
From the complete set of partition sequences, identify every partition sequence P_i (for a node set M_i) for which

(a) $M_i \subset M$

(b) P_i and P both occur in some attractor A

(Here, (a), (b) and Lemma S1.11 imply that P_i occurs in P)

Step 2:
Let k be the number of partition sequences from Step 1

Step 3:
Let $N = M \setminus (M_1 \cup ... \cup M_k)$

Step 4:
If $N \neq \varnothing$, use Procedure S1.6 to identify $S = \{\mathbf{x}_0^N, \mathbf{x}_1^N, ..., \mathbf{x}_{q-1}^N\}$ that occurs in P

Step 5:
If $N \neq \varnothing$, add S (identified in Step 4) to the set $\mathbf{U}$

At the end of the procedure, $\mathbf{U}$ contains every subsystem P satisfying the 3 properties of Definition 6. Essentially, property 1 is satisfied because of Step 4. Property 2 is satisfied because of Step 1 and the choice of N in step 3. Property 3 is satisfied because N is the largest set for which $M_i \cap N = \varnothing$ for all $i = 1, ..., k$. We demonstrate this formally with Theorem S1.19 (below), which is proved in Section S1.4.3

Theorem S1.19. Assume Procedure S1.18 begins with every partition sequence. Then, at the end of Procedure S1.18, the following is true

S is a subsystem (Definition 6 is satisfied)
$\iff$
S is equivalent to a partial state sequence $S^* \in \mathbf{U}$

PROOF: See Section S1.4.3

The following Theorem demonstrates that the set of all subsystems gives a complete coverage of the attractors

Theorem S1.20. Given an attractor A and node n_i, there exists a subsystem $S = \{\mathbf{x}_0^N, \mathbf{x}_1^N, ..., \mathbf{x}_{q-1}^N\}$ for which

(a) $n_i \in N$

(b) S occurs in A

PROOF : See Section S1.4.3

S1.4 Proofs for earlier results

For the Theorems and Lemmas in Section S1.1, Section S1.2 and Section S1.3, the proofs are given (in Sections S1.4.1, S1.4.2 and S1.4.3 respectively).

Some of these proofs refer to Lemmas S1.21 - S1.25. These can be found in Section S1.4.4.

S1.4.1 Proofs for Section S1.1

Theorem. S1.2

Consider a partial state sequence $P_x = \{\mathbf{x}_0^N, ..., \mathbf{x}_{q-1}^N\}$ that occurs in an attractor $A = \{\mathbf{z}_0, ..., \mathbf{z}_{p-1}\}$

A partial state sequence $P_w = \{\mathbf{w}_0^N, ..., \mathbf{w}_{r-1}^N\}$ (for the same node set N) occurs in $A \iff P_w$ is equivalent to P_x

PROOF:

Case: $\Longrightarrow$:

We assume $P_x = \{\mathbf{x}_0^N, ..., \mathbf{x}_{q-1}^N\}$ and $P_w = \{\mathbf{w}_0^N, ..., \mathbf{w}_{r-1}^N\}$ both occur in A. Therefore, by Definition 3 (main text), it is impossible to find a partial state sequence P' (for the same node set N) that satisfies properties 1 and 2 and has less partial states ($q' < q$).

Therefore, since P_x and P_w both involve the same node set N and both satisfy properties 1 and 2 of Definition 3, they must contain the same number of partial states. i.e.

A: $q = r$

Since P_x and P_w both occur in A, Lemmas S1.21 and S1.23 (in Section S1.4.4) and **A** imply that there exists integers l and m for which

B: For every $i \geq 0$, $\mathbf{x}_{l+i \pmod q}^N = \mathbf{w}_{m+i \pmod q}^N$

To show that P_x is equivalent to P_w, I need to show that the 3 properties of Definition S1.1 are satisfied. Property 1 is obvious because P_x and P_w involve the same node set N. Property 2 is satisfied because of $\mathbf{A}$. Property 3 is satisfied because of the following.

Let $c = m$ - $l \pmod q$.

Then for all $u = \{0, 1, ..., q-1\}$ and $i = q - l + u$, $\mathbf{B}$ implies

- $\mathbf{x}_u^N = \mathbf{x}_{l+i-q}^N = \mathbf{w}_{m+i-q \pmod q}^M = \mathbf{w}_{m-l+u \pmod q}^M = \mathbf{w}_{u+c \pmod q}^M = \mathbf{w}_v^M$

(where $v = u + c \pmod q$)

Therefore P_x is equivalent to P_w

Case: $\Longleftarrow$:

We assume that $P_x = \{\mathbf{x}_0^N, ..., \mathbf{x}_{q-1}^N\}$ occurs in $A = \{\mathbf{z}_0, ..., \mathbf{z}_{p-1}\}$,
and that $P_w = \{\mathbf{w}_0^N, ..., \mathbf{w}_{r-1}^N\}$ is equivalent to $P_x = \{\mathbf{x}_0^N, ..., \mathbf{x}_{q-1}^N\}$.

From Definition 3 of the main text,

C: The following are true for $P_x = \{\mathbf{x}_0^N, ..., \mathbf{x}_{q-1}^N\}$ and some sequence of integers $b_0, ..., b_{p-1} \in \{0, ..., q-1\}$

1. For $k = 0, ..., p-1$, $\mathbf{x}_{b_k}^N = \{s_i \in \mathbf{z}_k : n_i \in N\}$
2. For each $k \in \{0, ..., p-1\}$ and $j = k - 1 \pmod p$, either
   (a) $b_k = b_j$ or **(b)** $b_k = b_j + 1 \pmod q$
3. 1 and 2 are not true for any smaller partial state sequence $P' = \{\mathbf{y}_0^N, \mathbf{y}_1^N, ..., \mathbf{y}_{q'-1}^N\}$ and integers $d_0, ..., d_{p-1} \in \{0, ..., q'-1\}$ $(q' < q)$

From Definition S1.1, $q = r$ and there exists an integer c such that

D: $\mathbf{x}_i^N = \mathbf{w}_j^N$, for all $i \in \{0, 1, ..., q-1\}$ and $j = i + c \pmod q$

We want to show that P_w occurs in A (i.e. the 3 properties of Definition 3 are satisfied)

Letting $c_k = b_k + c \pmod q$, for $k = 0, ..., p-1$, $\mathbf{C}$ and $\mathbf{D}$ imply

1. For $k = 0, ..., p-1$, $\mathbf{w}_{c_k}^N = \mathbf{w}_{b_k+c \pmod q}^N\ \mathbf{x}_{b_k}^N = \{s_i \in \mathbf{z}_k : n_i \in N\}$
2. For each $k \in \{0, ..., p-1\}$ and $j = k - 1 \pmod p$, either

   (a) $c_k = b_k + c \pmod q = b_j + c \pmod q = c_j$

   (b) $c_k = b_k + c \pmod q = b_j + 1 + c \pmod q = c_j + 1 \pmod q$

Therefore, properties 1 and 2 of Definition 3 are satisfied (from 1 and 2 above). Property 3 is satisfied because P_w and P_x have the same number of partial states ($q = r$). Therefore, if property 3 failed for P_w, it would also fail for P_x (which is impossible since P_x occurs in A)

□

Theorem. S1.4

Consider a node set N, partial state sequence $P = \{\mathbf{x}_0^N, ..., \mathbf{x}_{q-1}^N\}$ and attractor $A = \{\mathbf{z}_0, \mathbf{z}_1, ..., \mathbf{z}_{p-1}\}$.

If any of one the following 3 statements (**A**, **B** or **C**) is true, they are all true. (i.e. $\mathbf{A} \iff \mathbf{B} \iff \mathbf{C} \iff \mathbf{A}$)

A: P occurs in A

B: Given N and A, Procedure S1.3 identifies a partial state sequence P^* that is equivalent to P (this could be $P^* = P$)

C: The following are true for $P = \{\mathbf{x}_0^N, ..., \mathbf{x}_{q-1}^N\}$ and some sequence of integers $b_0, ..., b_{p-1} \in \{0, ..., q-1\}$

1. For $k = 0, ..., p-1$, $\mathbf{x}_{b_k}^N = \{s_i \in \mathbf{z}_k : n_i \in N\}$
2. For each $k \in \{0, ..., p-1\}$ and $j = k - 1 \pmod p$, either **(a)** $b_k = b_j$ or **(b)** $b_k = b_j + 1 \pmod q$
3. Given $k \in \{0, ..., p-1\}$ and $j = k - 1 \pmod p$, **if** $b_k \neq b_j$ **then** $\mathbf{x}_{b_k}^N \neq \mathbf{x}_{b_j}^N$
4. For each $a \in \{0, ..., q-1\}$, $\exists$ $k \in \{0, ..., p-1\}$ such that $b_k = a$
5. There is no integer $q' \mid q$ $(q' < q)$ for which $\mathbf{x}_f^N = \mathbf{x}_g^N$ whenever f, g satisfies $f \pmod{q'} = g \pmod{q'}$

PROOF

Here, it is sufficient to prove the following 3 cases: $\mathbf{C} \implies \mathbf{A}$ and $\mathbf{A} \implies \mathbf{B}$ and $\mathbf{B} \implies \mathbf{C}$

Case: $\mathbf{C} \implies \mathbf{A}$

This follows from Lemma S1.23 (in Section S1.4.4).

Case: $\mathbf{A} \implies \mathbf{B}$

Suppose P occurs in A.

Given N and A, Procedure S1.3 identifies a partial state sequence $P^* = P^* = \{\mathbf{w}_0^N, ..., \mathbf{w}_{r-1}^N\}$

Therefore, by Lemmas S1.23 and S1.25 (in Section S1.4.4), P^* also occurs in A.

Therefore, by Theorem S1.2, P^* is equivalent to P (since they both involve the same node set N and occur in the same attractor A)

Case: $\mathbf{B} \implies \mathbf{C}$

Applying Procedure S1.3 to N and $A = \{\mathbf{z}_0, \mathbf{z}_1, ..., \mathbf{z}_{p-1}\}$, gives a partial state sequence $P^* = \{\mathbf{w}_0^N, ..., \mathbf{w}_{r-1}^N\}$. Then since P^* is equivalent to $P = \{\mathbf{x}_0^N, ..., \mathbf{x}_{q-1}^N\}$

D: $q = r$

E: $\exists$ an integer d such that, $\mathbf{x}_i^N = \mathbf{w}_j^N$, for all $i \in \{0, 1, ..., q-1\}$ and $j = i + d \pmod q$

Now by Lemma S1.25 (in Section S1.4.4),

F: The following are true for $P^* = \{\mathbf{w}_0^N, ..., \mathbf{w}_{q-1}^N\}$ and integers $c_0, ..., c_{p-1} \in \{0, ..., q-1\}$

1. For $k = 0, ..., p-1$, $\mathbf{w}_{c_k}^N = \{s_i \in \mathbf{z}_k : n_i \in N\}$
2. For each $k \in \{0, ..., p-1\}$ and $j = k-1 \pmod p$, either **(a)** $c_k = c_j$ or **(b)** $c_k = c_j + 1 \pmod q$
3. Given $k \in \{0, ..., p-1\}$ and $j = k-1 \pmod p$, **if** $c_k \neq c_j$ **then** $\mathbf{w}_{c_k}^N \neq \mathbf{w}_{c_j}^N$
4. For each $a \in \{0, ..., q-1\}$, $\exists$ $k \in \{0, ..., p-1\}$ such that $c_k = a$
5. There is no integer $q' \mid q$ $(q' < q)$ for which $\mathbf{w}_f^N = \mathbf{w}_g^N$ whenever f, g satisfies $f \pmod{q'} = g \pmod{q'}$

For $k = 0, ..., p-1$, let $b_k = c_k - d \pmod q$

Then we can show that properties **C**1-**C**5 are true for $P = \{\mathbf{x}_0^N, ..., \mathbf{x}_{q-1}^N\}$ and integers $b_0, ..., b_{p-1} \in \{0, ..., q-1\}$

*Property **C1**:*

For $k = 0, ..., p-1$, $\mathbf{x}_{b_k}^N = \mathbf{w}_j^N$ where $j = b_k + d \pmod q$ (by **E**)

Therefore (from **F1**), $\mathbf{x}_{b_k}^N = \mathbf{w}_{c_k}^N = \{s_i \in \mathbf{z}_k : n_i \in N\}$

*Property **C2**:*

For each $k \in \{0, ..., p-1\}$ and $j = k-1 \pmod p$, **E** and **F2** imply either

(a) $b_k = c_k - d \pmod q = c_j - d \pmod q = b_j$

(b) $b_k = c_k - d \pmod q = c_j + 1 - d \pmod q = b_j + 1 \pmod q$

*Property **C3**:*

Consider any $k \in \{0, ..., p-1\}$ and $j = k-1 \pmod p$.

Then if $b_k \neq b_j$, we have (following from **C**2and **F2**)

(i) $q > 1$

(ii) $b_k = c_k - d \pmod q = c_j + 1 - d \pmod q = b_j + 1 \pmod q$

(iii) $c_k \neq c_j$ (from (i) and (ii))

Therefore, from **F3**, $\mathbf{x}_{c_k}^N \neq \mathbf{x}_{c_j}^N$.

Therefore, $\mathbf{w}_{b_k}^N \neq \mathbf{w}_{b_j}^N$ (since $\mathbf{x}_{b_i}^N = \mathbf{w}_{c_i}^N$ for $i = 0, ..., p-1$: see **C**1)

Property ***C4***:

For each $a \in \{0, ..., q-1\}$, $\exists \quad k \in \{0, ..., p-1\}$ such that $c_k = a$ (from **F4**).

Therefore there also exists $k' \in \{0, ..., p-1\}$ such that

(i) $c_{k'} = a + d \pmod q$

(ii) $b_{k'} = c_{k'} - d \pmod q = a$

Property ***C5***:

Suppose there exists an integer $q' \mid q \quad (q' < q)$ for which $\mathbf{x}_f^N = \mathbf{x}_g^N$ whenever $f \pmod{q'} = g \pmod{q'}$.

Consider any f', g' satisfying $f' \pmod{q'} = g' \pmod{q'}$. Then

(a) $f' - d \pmod{q'} = g' - d \pmod{q'}$

(b) $(f' - d \pmod q) \pmod{q'} = (g' - d \pmod q) \pmod{q'}$

(by (a) and the fact q is a multiple of q')

(c) $\mathbf{x}^N_{f'-d \pmod q} = \mathbf{x}^N_{g'-d \pmod q}$

(by (b) and the fact $\mathbf{x}_f^N = \mathbf{x}_g^N$ whenever $f \pmod{q'} = g \pmod{q'}$)

Therefore, from (a) - (c) and **E**

- $\mathbf{w}^N_{f'} = \mathbf{x}^N_{f'-d \pmod q} = \mathbf{x}^N_{g'-d \pmod q} = \mathbf{w}^N_{g'}$

However, since this true for any f', g' satisfying $f' \pmod{q'} = g' \pmod{q'}$, it contradicts **F**5.

Therefore, there cannot be any integer $q' \mid q \quad (q' < q)$ for which $\mathbf{x}_f^N = \mathbf{x}_g^N$ whenever $f \pmod{q'} = g \pmod{q'}$

□

Theorem. S1.10

Given a node set N and set of attractors $\mathbb{C}$, Procedure S1.9 identifies partial state sequences P_1, ..., P_k and sets of attractors $\mathbb{C}_1$, ..., $\mathbb{C}_k$ satisfying

A: For $i = 1, ..., k$, P_i involves the node set N (i.e. $P_i = \{\mathbf{x}^N_{i_0}, ..., \mathbf{x}^N_{i_{q-1}}\}$)

B: For $i = 1, ..., k$, P_i occurs in every attractor $A \in \mathbb{C}_i$

C: For $i = 1, ..., k$, P_i does not occur in any attractor $A \notin \mathbb{C}_i$

D: For any i, j $(1 \leq i < j \leq k)$, $\mathbb{C}_i \cap \mathbb{C}_j = \varnothing$

E: $\mathbb{C}_1 \cup ... \cup \mathbb{C}_k = \mathbb{C}$

F: Given the node set N, there are no other partial state sequences $P' \notin \{P_1, ..., P_k\}$ that occur in any attractor $A \in \mathbb{C}$ (unless P' is equivalent to some $P_i \in \{P_1, ..., P_k\}$)

PROOF

Note that from Theorem S1.4, any partial state sequence Q_j created in Step 1 of the procedure must occur in A_j (when applying Procedure S1.3 to N and $A_j \in \mathbb{C}$)

Property A

Every partial state sequence in the procedure is created by applying Procedure S1.3 to N and some attractor $A \in \mathbb{C}$. This will only give partial state sequences involving the input node set N.

Properties B, C, D and F

Since all partial state sequences Q_j involve the node set N, Theorem S1.2 implies that if Q'_x is equivalent to Q'_y, then

- Q'_x occurs in an attractor $A \iff Q'_y$ occurs in an attractor A

Moreover, Theorem S1.2 implies that if Q'_x is **not** equivalent to Q'_y, then Q'_x and Q'_y never occur in the same attractor.

Therefore, from Step 2 and 3,

B: For $i = 1, ..., k$, P_i occurs in every attractor $A \in \mathbb{C}_i$

C: For $i = 1, ..., k$, P_i does not occur in any attractor $A \notin \mathbb{C}_i$

D: For any i, j $(1 \leq i < j \leq k)$, $\mathbb{C}_i \cap \mathbb{C}_j = \varnothing$

F: Given the node set N, there are no other partial state sequences $P' \notin \{P_1, ..., P_k\}$ that occur in any attractor $A \in \mathbb{C}$ (unless P' is equivalent to some $P_i \in \{P_1, ..., P_k\}$)

Property E

In Step 1, every $A_j \in \mathbb{C}$ is considered and a partial state sequence Q_j that occurs in A_j is created.

Since every Q_j is put into a group i and $\mathbb{C}_i = \{A_j : Q_j \text{ is part of the group } i\}$ (by Step 3), there exists $i \in \{1, ..., k\}$ for which $A_j \in \mathbb{C}_i$.

Therefore, since the procedure only uses attractors from $\mathbb{C}$

$\mathbb{C}_1 \cup ... \cup \mathbb{C}_k = \mathbb{C}$ (as required)

□

Lemma. S1.11

Consider two partial state sequences $P_x = \{\mathbf{x}_0^N, ..., \mathbf{x}_{q-1}^N\}$ and $P_y = \{\mathbf{y}_0^M, ..., \mathbf{y}_{r-1}^M\}$ (where $M \supseteq N$). Then,

(a) P_x occurs in P_y, and P_y occurs in an attractor $A \implies P_x$ occurs in A

(b) P_x and P_y both occur in an attractor $A \implies P_x$ occurs in P_y

PROOF

Part a

Since $P_x = \{\mathbf{x}_0^N, ..., \mathbf{x}_{q-1}^N\}$ occurs in $P_y = \{\mathbf{y}_0^M, ..., \mathbf{y}_{r-1}^M\}$ ($M \supseteq N$), and P_y occurs in an $A = \{\mathbf{z}_0, ..., \mathbf{z}_{p-1}\}$, Lemmas S1.23 and S1.24 (in Section S1.4.4) imply that

A: The following is true for $P_x = \{\mathbf{x}_0^N, ..., \mathbf{x}_{q-1}^N\}$ and some sequence of integers $b_0, ..., b_{r-1} \in \{0, ..., q-1\}$

1. For $k' = 0, ..., r-1$, $\mathbf{x}_{b_{k'}}^N = \{s_i \in \mathbf{y}_{k'}^M : n_i \in N \ (\subseteq M)\}$
2. For each $k' \in \{0, ..., r-1\}$ and $j' = k'-1 \pmod r$, either **(a)** $b_{k'} = b_{j'}$ or **(b)** $b_{k'} = b_{j'} + 1 \pmod q$
3. Given $k' \in \{0, ..., r-1\}$ and $j' = k'-1 \pmod r$, **if** $b_{k'} \neq b_{j'}$ **then** $\mathbf{x}_{b_{k'}}^N \neq \mathbf{x}_{b_{j'}}^N$
4. For each $a \in \{0, ..., q-1\}$, $\exists$ $k' \in \{0, ..., r-1\}$ such that $b_{k'} = a$
5. There is no integer $q' \mid q$ $(q' < q)$ for which $\mathbf{x}_f^N = \mathbf{x}_g^N$ whenever f, g satisfies $f \pmod{q'} = g \pmod{q'}$

B: The following is true for $P_y = \{\mathbf{y}_0^M, ..., \mathbf{y}_{r-1}^M\}$ and some sequence of integers $c_0, ..., c_{p-1} \in \{0, ..., r-1\}$

1. For $k = 0, ..., p-1$, $\mathbf{y}_{c_k}^N = \{s_i \in \mathbf{z}_k : n_i \in M\}$
2. For each $k \in \{0, ..., p-1\}$ and $j = k-1 \pmod p$, either **(a)** $c_k = c_j$ or **(b)** $c_k = c_j + 1 \pmod r$
3. Given $k \in \{0, ..., p-1\}$ and $j = k-1 \pmod p$, **if** $c_k \neq c_j$ **then** $\mathbf{y}_{c_k}^M \neq \mathbf{y}_{c_j}^M$
4. For each $a \in \{0, ..., r-1\}$, $\exists$ $k \in \{0, ..., p-1\}$ such that $c_k = a$
5. There is no integer $r' \mid r$ $(r' < r)$, for which $\mathbf{y}_f^M = \mathbf{y}_g^M$ whenever f, g satisfies $f \pmod{r'} = g \pmod{r'}$

For $k = 0, ..., p-1$, let $d_k = b_{c_k}$

Then $d_k \in \{0, ..., q-1\}$, since $c_k \in \{0, ..., r-1\}$ and $b_i \in \{0, ..., q-1\}$ for any $i \in \{0, ..., r-1\}$.

Then, following from **A** and **B** (and the fact that $N \subseteq M$)

C: The following is true for $P_x = \{\mathbf{x}_0^N, ..., \mathbf{x}_{q-1}^N\}$ and the sequence of integers $d_0, ..., d_{p-1} \in \{0, ..., q-1\}$ (proved below)

1. For $k = 0, ..., p-1$, $\mathbf{x}_{d_k}^N = \{s_i \in \mathbf{z}_k : n_i \in N\}$
2. For each $k \in \{0, ..., p-1\}$ and $j = k-1 \pmod p$, either **(a)** $d_k = d_j$ or **(b)** $d_k = d_j + 1 \pmod q$
3. Given $k \in \{0, ..., p-1\}$ and $j = k-1 \pmod p$, **if** $d_k \neq d_j$ **then** $\mathbf{x}_{d_k}^N \neq \mathbf{x}_{d_j}^N$
4. For each $a \in \{0, ..., q-1\}$, $\exists$ $k \in \{0, ..., p-1\}$ such that $d_k = a$
5. There is no integer $q' \mid q$ $(q' < q)$ for which $\mathbf{x}_f^N = \mathbf{x}_g^N$ whenever f, g satisfies $f \pmod{q'} = g \pmod{q'}$

Lemma S1.23 (in Section S1.4.4) then implies that P_x occurs in A (as required).

It just remains to show properties **C**1 - **C**5 are true

*Property **C1**:*

Let $k' = c_k$ in **A**1. Then, from **A**1 and **B**1

- For $k = 0, ..., p-1$, $\mathbf{x}_{d_k}^N = \mathbf{x}_{b_{k'}}^N = \{s_i \in \mathbf{y}_{k'}^M : n_i \in N\} = \{s_i \in \mathbf{y}_{c_k}^M : n_i \in N\} = \{s_i \in \mathbf{z}_k : n_i \in N\ (\subseteq M)\}$

*Property **C2**:*

We consider all possible cases from **A**2 and **B**2.

Given any $c_j \in \{1, ..., r\}$, let

- $k' = c_j + 1 \pmod r$ (in **A**2)
- $j' = k' - 1 \pmod r = c_j$ (in **A**2)

Then, for each $k \in \{0, ..., p-1\}$ and $j = k-1 \pmod p$, either

Case: B2(a)

$$d_k = b_{c_k} = b_{c_j} = d_j$$

Case: B2(b) and A2(a)

$$d_k = b_{c_k} = b_{c_j+1 \pmod r} = b_{k'} = b_{j'} = b_{c_j} = d_j$$

Case: B2(b) and A2(b)

$$d_k = b_{c_k} = b_{c_j+1 \pmod r} = b_{k'} = b_{j'} + 1 \pmod q = b_{c_j} + 1 \pmod q = d_j + 1 \pmod q$$

*Property **C**3:*

Given any $c_j \in \{1, ..., r\}$, let

- $k' = c_j + 1 \pmod{r}$ (in **A**3)
- $j' = k' - 1 \pmod{r} = c_j$ (in **A**3)

Then, given $k \in \{0, ..., p-1\}$ and $j = k - 1 \pmod{p}$, **if** $d_k \neq d_j$

(a) $b_{c_k} \neq b_{c_j}$

(b) $c_k \neq c_j$ (otherwise (a) would be incorrect)

(c) $c_k = c_j + 1 \pmod{r}$ (by (b) and **B**2)

(d) $k' \neq j'$ (by (b), (c) and choice of k', j')

(e) $\mathbf{x}^N_{b_{k'}} \neq \mathbf{x}^N_{b_{j'}}$ (by **A**3 and (d))

(f) $\mathbf{x}^N_{b_{c_k}} \neq \mathbf{x}^N_{b_{c_j}}$ (by (c), (e) and choice of k', j')

(g) $\mathbf{x}^N_{d_k} \neq \mathbf{x}^N_{d_j}$

(as required)

*Property **C**4:*

By **A**4. For each $a \in \{0, ..., q-1\}$, $\exists$ $k' \in \{0, ..., r-1\}$ such that $b_{k'} = a$

By **B**4. For each $k' \in \{0, ..., r-1\}$, $\exists$ $k \in \{0, ..., p-1\}$ such that $c_k = k'$

Therefore, for each $a \in \{0, ..., q-1\}$, $\exists$ $k \in \{0, ..., p-1\}$ such that $d_k = b_{c_k} = b_{k'} = a$

*Property **C**5:*

This follows directly from **A**5.

Part b

Suppose $P_x = \{\mathbf{x}^N_0, ..., \mathbf{x}^N_{q-1}\}$ and $P_y = \{\mathbf{y}^M_0, ..., \mathbf{y}^M_{r-1}\}$ $(M \supseteq N)$ both occur in an attractor $A = \{\mathbf{z}_0, ..., \mathbf{z}_{p-1}\}$

Applying Procedure S1.6 to P_y and N identifies a partial state sequence $P^* = \{\mathbf{w}^N_0, ..., \mathbf{w}^N_{q^*-1}\}$ that occurs in P_y (by Theorem S1.8)

Therefore, by **part a** of this Lemma, P^* occurs in A (since P^* occurs in P_y and P_y occurs in A)

Therefore, by Theorem S1.2, P_x is equivalent to P^* (since P^* and P_x both occur in A)

Therefore, by Theorem S1.7, P_x occurs in P_y

□

Lemma. S1.12

Consider two partial state sequences $P_x = \{\mathbf{x}_0^N, ..., \mathbf{x}_{q-1}^N\}$ and $P_y = \{\mathbf{y}_0^M, ..., \mathbf{y}_{r-1}^M\}$, and two sets of attractors $\mathbb{C}_x$ and $\mathbb{C}_y$ for which

1. $M \supseteq N$
2. P_x occurs in every attractor $A \in \mathbb{C}_x$
3. P_x does not occur in any attractor $A \notin \mathbb{C}_x$
4. P_y occurs in every attractor $A \in \mathbb{C}_y$
5. P_y does not occur in any attractor $A \notin \mathbb{C}_y$

Then, either

(a) $\mathbb{C}_x \cap \mathbb{C}_y = \varnothing$

(b) P_x occurs in P_y **and** $\mathbb{C}_y \subseteq \mathbb{C}_x$

PROOF

Suppose there exists an attractor $A \in \mathbb{C}_x \cap \mathbb{C}_y$ (i.e. (a) is not true).

Then P_x and P_y both occur in A. Therefore, by Lemma S1.11, P_x occurs in P_y.

Now consider any attractor $A \in \mathbb{C}_y$. Since P_x occurs in P_y, and P_y occurs in A, Lemma S1.11 implies that P_x also occurs in A. Therefore $A \in \mathbb{C}_x$ (and so $\mathbb{C}_y \subseteq \mathbb{C}_x$).

□

S1.4.2 Proofs for Section S1.2

Theorem. S1.14

At the end of Procedure S1.13, the following is true

P is an intersection sequence
$\Longleftrightarrow$
P is equivalent to a partial state sequence $P^* \in \{P^*, \mathbb{C}\} \in \mathbf{S}$

PROOF:

Case $\Longrightarrow$

Since $P = \{\mathbf{x}_0^N, ..., \mathbf{x}_{q-1}^N\}$ is an intersection sequence, Definition 4 (in the main text) implies that there is a set of attractors $\mathbb{C}$ for which

1. P occurs in every attractor $A \in \mathbb{C}$
2. P does not occur in any attractor $A \notin \mathbb{C}$

Moreover, P must be equivalent to some partial state sequence $P^* = \{\mathbf{w}_0^N, ..., \mathbf{w}_{q-1}^N\}$ identified in Step 2 of Procedure S1.13, when the node set N is analysed (because of (f) in Step 2)

Therefore, by Theorem S1.2,

1. P^* occurs in every attractor $A \in \mathbb{C}$
2. P^* does not occur in any attractor $A \notin \mathbb{C}$

and so $\{P^*, \mathbb{C}\}$ is added to $\mathbf{S}$ in Step 3 (of Procedure S1.13).

It just remains to show that $\{P^*, \mathbb{C}\}$ is never removed from $\mathbf{S}$ in Step 4 of any loop. $\{P^*, \mathbb{C}\}$ could only be removed in Step 4 if there was a pair $\{Q = \{\mathbf{y}_0^M, ..., \mathbf{y}_{r-1}^M\}, \mathbb{D}\}$ for which

(i) $M \supset N$

(ii) $\mathbb{D} = \mathbb{C}$

(iii) Q occurs in every attractor $A \in \mathbb{D} = \mathbb{C}$ (because of Step 2(b) and (ii))

However, this would be impossible since P is an intersection sequence, and so property 3 of Definition 4 implies that

- Given a larger set $M \supset N$, there is no partial state sequence Q for the node set M that occurs in every attractor $A \in \mathbb{C}$ $(= \mathbb{D})$

Case $\Longleftarrow$

We show that P^* is an intersection sequence, that intersects at $\mathbb{C}$ (i.e. the 3 properties of Definition 4 (main text) hold for P^* and $\mathbb{C}$). It then follows from Theorem S1.2 and Definition 4 (main text) that the same must be true for any P, which is equivalent to P^*.

Properties 1 and 2 are true because of (b) and (c) in Step 2 of Procedure S1.13. Therefore,

(b) $P^* = \{\mathbf{w}_0^N, ..., \mathbf{w}_{q-1}^N\}$ occurs in every attractor $A \in \mathbb{C}$

(c) $P^* = \{\mathbf{w}_0^N, ..., \mathbf{w}_{q-1}^N\}$ does not occur in any attractor $A \notin \mathbb{C}$

Now, we show that property 3 holds (noting that every node set M is analysed at some point in the procedure).

If a partial state sequence Q (for a node set $M \supset N$) occurs in every $A \in \mathbb{C}$, (b), (c) and Lemma S1.12 implies that

- Q occurs in every attractor $A \in \mathbb{C}$
- Q does not occur in any attractor $A \notin \mathbb{C}$

Let M be the largest such set (i.e. it is impossible to identify another partial state sequence Q' for a node set $L \supset M \supset N$). Then, when M is analysed in the procedure, we get

Step 2: Q would be identified (or an equivalent partial state sequence would be identified).

Step 3: $\{Q,\mathbb{C}\}$ would be added to $\mathbf{S}$

Step 4: Either

A : Case M is analysed after N
$\{P^*,\mathbb{C}\}$ is removed from $\mathbf{S}$

B : Case M is analysed before N
$\{Q,\mathbb{C}\}$ is kept in $\mathbf{S}$ and $\{P^*,\mathbb{C}\}$ is removed from $\mathbf{S}$ when it is analysed later

Therefore, in order for $\{P^*,\mathbb{C}\}$ to be in $\mathbf{S}$ at the end of the procedure, there cannot be any partial state sequence Q (for a larger node set $M \supset N$) that occurs in every $A \in \mathbb{C}$. Therefore Property 3 of Definition 4 is satisfied.

$\square$

Theorem. S1.15

Consider a partial state sequence $P = \{\mathbf{x}_0^N, ..., \mathbf{x}_{q-1}^N\}$ for which

1. P occurs in a single attractor $A = \{\mathbf{z}_0, ..., \mathbf{z}_{p-1}\}$
2. P does not occur in any attractor $A' \neq A$

Then, given that V is the set of all nodes,

(a) Given a node set M satisfying $N \subset M \subset V$, it is impossible to find an intersection sequence that involves the node set M and occurs in A.

(b) Given the node set V, it is possible to find an intersection sequence P' that involves the node set V and occurs in A (usually A itself).

PROOF:

Applying Procedure S1.3 for the node set V and an attractor A, gives a partial state sequence P' that involves the node set V and occurs in A (by Theorem S1.4)

Now consider any partial state sequence $Q = \{\mathbf{y}_0^M, ..., \mathbf{y}_{r-1}^M\}$ that involves a node set M ($N \subset M$) and occurs in the attractor A. Then, from 1., 2. and Lemma S1.12,

- P occurs in Q
- Q does not occur in any attractor $A' \neq A$

Therefore, A is the only attractor for which Q occurs in A (this includes $Q = P'$, if $M = V$)

Therefore,

(a) Given a node set M satisfying $N \subset M \subset V$, any partial state sequence Q that occurs in A will fail Definition 4 (main text). Since,

1. Q occurs in A (from above)
2. Q does not occur in any attractor $A' \neq A$ (from above)
3. There exists a partial state sequence P' (for a larger node set $V \supset M$) that occurs in A.

(b) P' is an intersection sequences since P' and A satisfy the 3 properties of Definition 4 (main text). Namely,

1. P' occurs in A (from above)
2. P' does not occur in any attractor $A' \neq A$ (from above)
3. There is no node set larger than V

Therefore, parts (a) and (b) of the Theorem are true.

□

Theorem. S1.17

At the end of Procedure S1.16, the following is true

P that is core to some set of attractors $\mathbb{C}$ (Definition 5A is satisfied)
$\iff$
P is equivalent to a partial state sequence $P^* \in \{P^*, \mathbb{C}\} \in \mathbf{T}$

PROOF:

Case $\Longleftarrow$

We show that the 3 properties of Definition 5A hold for any $P^* \in \{P^*, \mathbb{C}\} \in \mathbf{T}$.

It then follows from Theorem S1.2, Theorem S1.7, Definition 5A and Definition S1.1 that the same must be true for any P, which is equivalent to P^*.

$\{P^* = \{\mathbf{x}_0^N, ..., \mathbf{x}_{q-1}^N\}, \mathbb{C}\}$ is added to $\mathbf{T}$ when Steps 1 - 5 of Procedure S1.16 are carried out for some intersection sequence P' (that intersects at $\mathbb{C}$). We consider Steps 1 - 5 for P', when showing the 3 properties of Definition 5A hold for P^*

Property 1 is satisfied because Step 4 implies P^* occurs in P' (which intersects at $\mathbb{C}$)

Property 2 is satisfied because of the following.

Consider any intersection sequence Q (for a node set M) that intersects at $\mathbb{D}$ (where $\mathbb{D} \cap \mathbb{C} \neq \varnothing$). We need to show that there is an intersection sequence Q' (for a node set $M' \supseteq M \cup N$) that occurs in every node set $A \in \mathbb{D} \cap \mathbb{C}$.

Let Q' be any intersection sequence (for a node set M') satisfying

(a) $M' \supseteq M$

(b) Q' intersects at $\mathbb{D}' \supseteq \mathbb{D} \cap \mathbb{C}$ (this also implies $\mathbb{D}' \cap \mathbb{C} \neq \varnothing$)

(c) There is no intersection sequence Q^* (for a larger node set $M^* \supset M' \supseteq M$) that intersects at $\mathbb{D}^* \supseteq (\mathbb{D}' \cap \mathbb{C}) \supseteq (\mathbb{D} \cap \mathbb{C})$

Now, at least one Q' must exist since Q itself satisfies (a) and (b). Therefore, either Q satisfies (a), (b) and (c) or we can find $Q' = Q^*$ (for the largest possible node set $M^* \supseteq M$) that does. Q' is then identified in Step 1 of the procedure and so

(d) $M' \supseteq N$ (from Step 3)

(e) $M' \supseteq M \cup N$ (from (a) and (d))

Moreover, because of (b) and Definition 4

(f) Q' occurs in every $A \in \mathbb{D} \cap \mathbb{C}$

Therefore, because of (e) and (f), we have found a suitable Q'.

Property 3 is satisfied because of the following. Consider any partial state sequence P'', for a larger node set $N'' \supset N$. Then, since $N = M_1 \cap ... \cap M_k$ in Step 3, there must be a node set M_i ($i \in \{1, ..., k\}$) satisfying

- $M_i \not\supseteq N''$

Then, for the corresponding intersection sequence Q_i (for the node set M_i) identified in Step 1

(i) Q_i intersects at $\mathbb{D}_i$, where $\mathbb{D}_i \cap \mathbb{C} \neq \varnothing$ (by Step 1a)

(ii) There is no intersection sequence Q^* (for a larger node set $M^* \supset M_i$) that intersects at $\mathbb{D}^* \supseteq (\mathbb{D}_i \cap \mathbb{C})$ (by Step 1b)

(ii) There is no intersection sequence, Q^* (for a larger node set $M^* \supset M_i$) that occurs in every attractor $A \in \mathbb{D}_i \cap \mathbb{C}$ (by (ii) and Definition 4)

Therefore, it is impossible to find an intersection sequence Q^* (for a node set $M^* \supseteq M_i \cup N''$) that occurs in every attractor $A \in \mathbb{D}_i \cap \mathbb{C}$. Therefore, P'' cannot be core to $\mathbb{C}$, since property 2 of Definition 5A would fail (for $Q = Q_i$).

Case $\Longrightarrow$

Suppose $P = \{\mathbf{x}_0^N, ..., \mathbf{x}_{q-1}^N\}$ is core to some set of attractors $\mathbb{C}$ (say). Then, Definition 5A (main text) implies that

1. P occurs in an intersection sequence P', which intersects at $\mathbb{C}$ (P can equal P').
2. If an intersection sequence Q (for a node set M) intersects at $\mathbb{D}$ (where $\mathbb{D} \cap \mathbb{C} \neq \varnothing$), then there exists an intersection sequence Q' (for a node set $M' \supseteq M \cup N$) that occurs in every attractor $A \in \mathbb{D} \cap \mathbb{C}$
3. 1 and 2 are not true for any larger partial state sequence P'' (for a node set $N'' \supset N$)

Now, $P' = \{\mathbf{y}_0^{N'}, \mathbf{y}_1^{N'}, ..., \mathbf{y}_{r-1}^{N'}\}$ (from 1. above) will be analysed in Procedure S1.16.

In Step 1, intersection sequences $Q_1, ..., Q_k$ (for node sets $M_1, ..., M_k$ resp) are identified where (for $i = 1, ..., k$)

A: Q_i intersects at $\mathbb{D}_i$, where $\mathbb{D}_i \cap \mathbb{C} \neq \varnothing$

B: There is no intersection sequence Q_i^* (for a node set $M_i^* \supset M_i$) that intersects at $\mathbb{D}_i^* \supseteq (\mathbb{D}_i \cap \mathbb{C})$

C: There is no intersection sequence Q_i^* (for a node set $M_i^* \supset M_i$) that occurs in every attractor $A \in \mathbb{D}_i \cap \mathbb{C}$ (by **B** and Definition 4)

Moreover, $P' \in \{Q_1, ..., Q_k\}$, since

A′: P' intersects at $\mathbb{C}$, where $\mathbb{C} \cap \mathbb{C} = \mathbb{C} \neq \varnothing$

B′: There is no intersection sequence P'' (for a node set $M'' \supset N'$) that intersects at $\mathbb{D}' \supseteq \mathbb{C}$ (by Definition 4)

Now, because of **A** and 2. above, the following are true for $i = 1, ..., k$

D: There exists an intersection sequence Q_i^* (for a node set $M_i^* \supseteq M_i \cup N$) that occurs in every attractor $A \in \mathbb{D}_i \cap \mathbb{C}$

Therefore, comparing **C** and **D**, it must be the case that $N \subseteq M_i$ (for $i = 1, ..., k$). Therefore,

E: $N \subseteq (M_1 \cap ... \cap M_k) \subseteq N'$ (since $P' \in \{Q_1, ..., Q_k\}$)

Now, in Steps 3 - 5 of Procedure S1.16 (when P' is analysed), we identify a node set N^* and partial state sequence P^* where

- $N^* = (M_1 \cap ... \cap M_k) \subseteq N'$
- $P^* = \{\mathbf{w}_0^{N^*}, ..., \mathbf{w}_{q^*-1}^{N^*}\}$ occurs in P'
- The pair $\{P^*, \mathbb{C}\}$ is added to the set $\mathbf{T}$

Therefore, by part $\Longleftarrow$ of this Theorem,

- P^* is core to $\mathbb{C}$

Therefore, P and P^* both occur in P' and are both core to $\mathbb{C}$.

Therefore, because of 2. and 3. and Theorem S1.7, it must be the case that $N = N^*$ and P is equivalent to P^* ($\in \{P^*,\mathbb{C}\} \in \mathbf{T}$) as required

□

S1.4.3 Proofs for Section S1.3

Theorem. S1.19

Assume Procedure S1.18 begins with every partition sequence. Then, at the end of Procedure S1.18, the following is true

S is a subsystem (Definition 6 is satisfied)
$\Longleftrightarrow$
S is equivalent to a partial state sequence $S^* \in \mathbf{U}$

PROOF:

Case $\Longleftarrow$

We show that the 3 properties of Definition 6 hold for any $S^* \in \mathbf{U}$.

It then follows from Theorem S1.2, Theorem S1.7, Definition 6 and Definition S1.1 that the same must be true for any S, which is equivalent to S^*.

$S^* = \{\mathbf{x}_0^N, ..., \mathbf{x}_{q-1}^N\}$ is added to $\mathbf{U}$ when Steps 1 - 5 of Procedure S1.18 are carried out for some partition sequence $P = \{\mathbf{y}_0^M, ..., \mathbf{y}_{r-1}^M\}$. We consider Steps 1 - 5 for P, when showing the 3 properties of Definition 6 hold for S^*

Property 1 is satisfied because Step 4 implies S^* occurs in P.

Property 2 is satisfied because of the following. Consider any partition sequence P' (for a node set $M' \subset M$) that occurs in P. Then, Lemma S1.11 implies that P' occurs in an attractor A, whenever P occurs in A.

Therefore, P' is identified in Step 1, and $N \subseteq (M \setminus M')$ (by Step 3 and the fact $M' = M_i$ for some $i \in \{1, ..., k\}$).

Therefore, $M' \cap N = \varnothing$ and property 2 holds

Property 3 is satisfied because of the following. Consider any partial state sequence S' (for a larger node set $N' \supset N$) that occurs in P.

Then, since $N = M \setminus (M_1 \cup ... \cup M_k)$ in Step 3, there must be a node set M_i ($i \in \{1, ..., k\}$) satisfying

- $M_i \cap N' \neq \varnothing$

(since S' occurs in $P = \{\mathbf{y}_0^M, ..., \mathbf{y}_{r-1}^M\}$, and so $N' \subseteq M$)

Therefore property 2 of Definition 6 would fail.

Case $\Longrightarrow$

Suppose $S = \{\mathbf{x}_0^N, ..., \mathbf{x}_{q-1}^N\}$ is subsystem. Then, Definition 6 implies that there exists a partition sequence $P = \{\mathbf{y}_0^M, ..., \mathbf{y}_{r-1}^M\}$ for which

1. S occurs in P (and so $N \subseteq M$)
2. If another partition sequence P' (for a node set $M' \subset M$) occurs in P, then $M' \cap N = \varnothing$
3. 1 and 2 are not true for any partial state sequence S', for a larger node set $N' \supset N$

Now, P (from above) will be analysed in Procedure S1.18. In Step 1 partition sequences P_1, ..., P_k (for node sets M_1, ..., M_k resp) are identified where (for $i = 1, ..., k$)

A: $M_i \subset M$

B: P_i and P both occur in some attractor A_i

C: P_i occurs in P (by **A**, **B** and Lemma S1.11)

Therefore, because of **A**, **C** and 2. above, the following are true

D: For $i = 1, ..., k, \quad M_i \cap N = \varnothing$

E: $(M_1 \cup ... \cup M_k) \cap N = \varnothing$ (by **D**)

F: $N \subseteq M \setminus (M_1 \cup ... \cup M_k)$ (by **A**, **E** and 1. from above)

Now, in Steps 3 - 5 of Procedure S1.18 (when P is analysed), we identify a node set N^* and partial state sequence S^* where

- $N^* = M \setminus (M_1 \cup ... \cup M_k) \supseteq N$
- $S^* = \{\mathbf{w}_0^{N^*}, ..., \mathbf{w}_{q^*-1}^{N^*}\}$ occurs in P
- S^* is added to the set $\mathbf{U}$

Therefore, by part $\Longleftarrow$ of this Theorem,

- S^* is a subsystem and satisfies properties 1 - 3

Therefore, because of 1. - 3. and Theorem S1.7, it must be the case that $N = N^*$ and S is equivalent to $S^* \in \mathbf{U}$, as required

Theorem. S1.20

Given an attractor A and node n_i, there exists a subsystem $S = \{\mathbf{x}_0^N, \mathbf{x}_1^N, ..., \mathbf{x}_{q-1}^N\}$ for which

(a) $n_i \in N$

(b) S occurs in A

PROOF :

Given the node set V (set of all nodes) and the attractor A, Procedure S1.3 identifies a partial state sequence P that occurs in A (by Theorem S1.4). Now, let $\mathbb{C}$ be the set of attractors for which

1. P occurs in every attractor $A \in \mathbb{C}$
2. P does not occur in any attractor $A \notin \mathbb{C}$

(Note: In a Boolean network model, $P = A$ and $\mathbb{C} = \{A\}$)

Therefore, since V is the largest possible node set, 1., 2. and Definition 4 imply P is an intersection sequence that intersects at $\mathbb{C}$.

Moreover, no other intersection sequence P' (that is not equivalent to P) can intersect at $\mathbb{C}$ (because of 1 and 2 and the fact that V is the largest node set possible). Therefore, P is exclusive to $\mathbb{C}$ and is a partition sequence (by Definition 5).

Therefore, it is possible to find a partition sequence P (for a node set $M = V$) for which

(a) $n_i \in M$

(b) P occurs in A

Now carry out the following loop

while() {

If a partition sequence P_i (for a node set M_i) exists such that

- $n_i \in M_i$
- $M_i \subset M$
- P_i occurs in P (and P_i occurs in A by Lemma S1.11)

Then, replace M by M_i, replace P by P_i and execute the loop again.

Otherwise, **exit loop**

}

At the end of the loop we have a partition sequence P (for the node set M) satisfying

A: $n_i \in M$

B: P occurs in A

C: If a partition sequence P' (for a node set $M' \subset M$) occurs in P, then $M' \cap \{n_i\} = \varnothing$

Now, we apply Steps 1-5 of Procedure S1.18 to P. In Step 1, partition sequences P_1, ..., P_k (for node sets M_1, ..., M_k resp) are identified where

D: For $i = 1$, ..., k, $M_i \subset M$ (by Step 1a)

E: For $i = 1$, ..., k, P_i and P both occur in some attractor A_i (by Step 1b)

F: For $i = 1$, ..., k, P_i occurs in P (by **D**, **E** and Lemma S1.11)

G: For $i = 1$, ..., k, $M_i \cap \{n_i\} = \varnothing$ (by **C**, **D** and **F**)

Then, in Steps 3-5, we identify a node set N and partial state sequence $S = \{\mathbf{x}_0^N, \mathbf{x}_1^N, ..., \mathbf{x}_{q-1}^N\}$ where

H: $N = M \setminus (M_1 \cup ... \cup M_k)$ (by Step 3)

I: $n_i \in N$ (by **A**, **G** and **H**)

J: S occurs in P (by Step 4)

K: S occurs in A (by **B**, **J** and and Lemma S1.11)

L: S is added to the set $\mathbf{U}$ (by Step 5)

Therefore, by **L** and part $\Longleftarrow$ of Theorem S1.19,

M: S is a subsystem

Therefore, because of **I**, **K** and **M**, the theorem is satisfied.

□

S1.4.4 Supplementary Lemmas

Lemma S1.21. Consider an attractor $A = \{\mathbf{z}_0, ..., \mathbf{z}_{p-1}\}$, and two partial state sequences $P_x = \{\mathbf{x}_0^N, ..., \mathbf{x}_{q-1}^N\}$ and $P_w = \{\mathbf{w}_0^N, ..., \mathbf{w}_{r-1}^N\}$ for the same node set N.

Now suppose,

A: The following are true for $P_x = \{\mathbf{x}_0^N, ..., \mathbf{x}_{q-1}^N\}$ and some sequence of integers $b_0, ..., b_{p-1} \in \{0, ..., q-1\}$

1. For $k = 0, ..., p-1,\quad \mathbf{x}_{b_k}^N = \{s_i \in \mathbf{z}_k : n_i \in N\}$
2. For each $k \in \{0, ..., p-1\}$ and $j = k-1 \pmod p$, either **(a)** $b_k = b_j$ or **(b)** $b_k = b_j + 1 \pmod q$
3. Given $k \in \{0, ..., p-1\}$ and $j = k-1 \pmod p$, **if** $b_k \neq b_j$ **then** $\mathbf{x}_{b_k}^N \neq \mathbf{x}_{b_j}^N$
4. For each $a \in \{0, ..., q-1\}$, $\exists\ k \in \{0, ..., p-1\}$ such that $b_k = a$

B: The following are true for $P_w = \{\mathbf{w}_0^N, ..., \mathbf{w}_{r-1}^N\}$ and some sequence of integers $c_0, ..., c_{p-1} \in \{0, ..., r-1\}$

1. For $k = 0, ..., p-1,\quad \mathbf{w}_{c_k}^N = \{s_i \in \mathbf{z}_k : n_i \in N\}$
2. For each $k \in \{0, ..., p-1\}$ and $j = k-1 \pmod p$, either **(a)** $c_k = c_j$ or **(b)** $c_k = c_j + 1 \pmod r$
3. Given $k \in \{0, ..., p-1\}$ and $j = k-1 \pmod p$, **if** $c_k \neq c_j$ **then** $\mathbf{w}_{c_k}^N \neq \mathbf{w}_{c_j}^N$
4. For each $a \in \{0, ..., r-1\}$, $\exists\ k \in \{0, ..., p-1\}$ such that $c_k = a$

Then, letting $l = b_0$ and $m = c_0$

C: For every $i \geq 0$

1. $\exists\ k_i \in \{0, ..., p-1\}$ for which $b_{k_i} = l + i \pmod q$ and $c_{k_i} = m + i \pmod r$
2. $\mathbf{x}_{l+i \pmod q}^N = \mathbf{w}_{m+i \pmod r}^N$

PROOF

From **A**1 and **B**1,

D: For $k = 0, ..., p-1,\quad \mathbf{x}_{b_k}^N = \mathbf{w}_{c_k}^N = \{s_i \in \mathbf{z}_k : n_i \in N\}$

Additionally, note that

E: If $q = 1$, then $r = 1$ (because of the (i)-(iv) below)

(i) For $k = 0, ..., p-1,\quad b_k = 0$

(ii) For $k = 0, ..., p-1,\quad \mathbf{w}_{c_k}^N = \mathbf{x}_0^N$ (by (i) and **D**)

(iii) Given $k \in \{0, ..., p-1\}$ and $j = k-1 \pmod p$, $\quad c_k = c_j$ (by (ii) and **B**3)

(iv) $r = 1$ and $c_k = 0$, for $k = 0, ..., p-1$ (by (iii) and **B**4)

We want to prove **C** is true. We prove **C**1 here. **C**2 then follows from **C**1 and **D**.

For the case $i = 0$, letting $k_i = 0$ ensures $b_{k_i} = b_0 = l = l + i \pmod q$ and $c_{m_i} = c_0 = m = m + i \pmod r$ (as required)

For the case $q = 1$, we have $r = 1$ (by **E**) and so $k_i = 0$, for every $i > 0$. This then ensures $b_{k_i} = b_0 = 0 = l + i \pmod 1$ and $c_{m_i} = c_0 = 0 = m + i \pmod 1$ (as required)

Therefore, it just remains to prove the case $i > 0, q > 1, r > 1$. We do this by induction on i (case $i = 0$ already done). Suppose **C**1 is true for $i - 1$ (≥ 0), so that

★ $\exists \;\; k_{i-1} \in \{0, ..., p-1\}$ for which $b_{k_{i-1}} = l + i - 1 \pmod q$ and $c_{k_{i-1}} = m + i - 1 \pmod r$

Because of **A**2, **A**3 and **A**4 and the fact that $q > 1$, there must exist a chain of integers

- j_0, ..., j_d, k

for which

(a) For $t = 0$, ..., d, $\;\; j_t = k_{i-1} + t \pmod p$

(b) $k = j_d + 1 \pmod p$

(c) $b_{j_0} = ... = b_{j_d} \neq b_k$ and $b_k = b_{j_d} + 1 \pmod q$

(d) $\mathbf{x}^N_{b_{j_0}} = ... = \mathbf{x}^N_{b_{j_d}} \neq \mathbf{x}^N_{b_k}$

Then, because of (a)- (d), **B**3, **D** and the fact that $q > 1$ and $r > 1$,

(e) $c_{j_0} = ... = c_{j_d} \neq c_k$ and $c_k = c_{j_d} + 1 \pmod r$

(f) $\mathbf{w}^N_{c_{j_0}} = ... = \mathbf{w}^N_{c_{j_d}} \neq \mathbf{w}^N_{c_k}$

Then letting $k_i = k$ (and noting that $b_{k_{i-1}} = b_{j_0}$ and $c_{k_{i-1}} = c_{j_0}$), (a) - (f) and ★ imply that

(g) $b_{k_i} = b_{j_d} + 1 \pmod q$
$= b_{k_{i-1}} + 1 \pmod q$
$= (l + i - 1 \pmod q) + 1) \pmod q$
$= l + i \pmod q$

(h) $c_{k_i} = c_{j_d} + 1 \pmod r$
$= c_{k_{i-1}} + 1 \pmod r$
$= (m + i - 1 \pmod r) + 1) \pmod r$
$= m + i \pmod r$

as required and so **C**1 (and **C**2) holds.

□

Lemma S1.22. Consider an attractor $A = \{\mathbf{z}_0, ..., \mathbf{z}_{p-1}\}$, and two partial state sequences $P_x = \{\mathbf{x}_0^N, ..., \mathbf{x}_{q-1}^N\}$ and $P_w = \{\mathbf{w}_0^N, ..., \mathbf{w}_{r-1}^N\}$ for the same node set N.

Now suppose,

A: The following are true for $P_x = \{\mathbf{x}_0^N, ..., \mathbf{x}_{q-1}^N\}$ and some sequence of integers $b_0, ..., b_{p-1} \in \{0, ..., q-1\}$

1. For $k = 0, ..., p-1$, $\quad \mathbf{x}_{b_k}^N = \{s_i \in \mathbf{z}_k : n_i \in N\}$
2. For each $k \in \{0, ..., p-1\}$ and $j = k-1 \pmod p$, either **(a)** $b_k = b_j$ or **(b)** $b_k = b_j + 1 \pmod q$
3. Given $k \in \{0, ..., p-1\}$ and $j = k-1 \pmod p$, **if** $b_k \neq b_j$ **then** $\mathbf{x}_{b_k}^N \neq \mathbf{x}_{b_j}^N$
4. For each $a \in \{0, ..., q-1\}$, $\exists \quad k \in \{0, ..., p-1\}$ such that $b_k = a$

B: The following are true for $P_w = \{\mathbf{w}_0^N, ..., \mathbf{w}_{r-1}^N\}$ and some sequence of integers $c_0, ..., c_{p-1} \in \{0, ..., r-1\}$

1. For $k = 0, ..., p-1$, $\quad \mathbf{w}_{c_k}^N = \{s_i \in \mathbf{z}_k : n_i \in N\}$
2. For each $k \in \{0, ..., p-1\}$ and $j = k-1 \pmod p$, either **(a)** $c_k = c_j$ or **(b)** $c_k = c_j + 1 \pmod r$
3. Given $k \in \{0, ..., p-1\}$ and $j = k-1 \pmod p$, **if** $c_k \neq c_j$ **then** $\mathbf{w}_{c_k}^N \neq \mathbf{w}_{c_j}^N$
4. For each $a \in \{0, ..., r-1\}$, $\exists \quad k \in \{0, ..., p-1\}$ such that $c_k = a$

Then, letting h be the highest common factor of q and r,

(i) $\mathbf{x}_f^N = \mathbf{x}_g^N$ whenever $f \pmod h = g \pmod h$ $\quad (0 \leq f \leq q-1, 0 \leq g \leq q-1)$

(ii) $P_z = \{\mathbf{x}_0^N, ..., \mathbf{x}_{h-1}^N\}$ satisfies Properties 1 and 2 of Definition 3

PROOF

From Lemma S1.21, the following is true for $l = b_0$ and $m = c_0$

C: For every $i \geq 0$

1. $\exists \quad k_i \in \{0, ..., p-1\}$ for which $b_{k_i} = l + i \pmod q$ and $c_{k_i} = m + i \pmod r$
2. $\mathbf{x}_{l+i \pmod q}^N = \mathbf{w}_{m+i \pmod r}^N$

Let h be the highest common factor of q and r and consider any integers f, g $\quad (\leq q-1)$ satisfying $f \pmod h = g \pmod h$. Then we have,

D: $\quad g = f + i_1 h$ for some integer i_1

E: $\quad h = i_2 q + i_3 r$ for some integers i_2, i_3 (by Euclid's algorithm)

Let $i' = f - l + i_1 i_3 r + i_4 q$

(where i_4 is any non-negative integer, larger enough to ensure $i' \geq 0$, $i' - i_1 i_3 r \geq 0$).

Then **C**, **D** and **E** leads to

F: $$\begin{aligned} g &= f + i_1 h \\ &= f + i_1 i_2 q + i_1 i_3 r \\ &= l + i' - i_4 q + i_1 i_2 q \\ &= l + i' \pmod q \end{aligned}$$

G: $$\begin{aligned} \mathbf{x}_f^N &= \mathbf{x}^N_{l+i'-i_1 i_3 r - i_4 q} \\ &= \mathbf{x}^N_{l+i'-i_1 i_3 r \pmod q} \\ &= \mathbf{w}^N_{m+i'-i_1 i_3 r \pmod r} \\ &= \mathbf{w}^N_{m+i' \pmod r} \\ &= \mathbf{x}^N_{l+i' \pmod q} \\ &= \mathbf{w}^N_g \end{aligned}$$

Therefore part (i) of the Lemma is true because $\mathbf{x}_f^N = \mathbf{x}_g^N$ whenever $f \pmod h = g \pmod h$ $(0 \le f \le q-1,\ 0 \le g \le q-1)$

Now, letting $s_k = b_k \pmod h$, for $k = 0, ..., p-1$

H: The following are true for $P_z = \{\mathbf{x}_0^N, ..., \mathbf{x}_{h-1}^N\}$ and integers $s_0, ..., s_{p-1} \in \{0, ..., h-1\}$

1. For $k = 0, ..., p-1$, $\mathbf{x}^N_{s_k} = \mathbf{x}^N_{b_k} = \{s_i \in \mathbf{z}_k : n_i \in N\}$ (by **G**)
2. For each $k \in \{0, ..., p-1\}$ and $j = k-1 \pmod p$, either
   (a) $s_k = b_k \pmod h = b_j \pmod h = b_j$
   (b) $s_k = b_k \pmod h = (b_j{+}1 \pmod q) \pmod h = b_j{+}1 \pmod h = s_j{+}1 \pmod h$ (because q is a multiple of h)

Therefore, part (ii) of the Lemma is true because $P_z = \{\mathbf{x}_0^N, ..., \mathbf{x}_{h-1}^N\}$ satisfies the first two properties of Definition 3 (as required).

□

Lemma S1.23. Consider a partial state sequence $P = \{\mathbf{x}_0^N, ..., \mathbf{x}_{q-1}^N\}$ and an attractor $A = \{\mathbf{z}_0, ..., \mathbf{z}_{p-1}\}$

P occurs in A (i.e. Definition 3 is satisfied)
$\Longleftrightarrow$
The following are true for $P = \{\mathbf{x}_0^N, ..., \mathbf{x}_{q-1}^N\}$ and some sequence of integers $b_0, ..., b_{p-1} \in \{0, ..., q-1\}$

1. For $k = 0, ..., p-1$, $\mathbf{x}^N_{b_k} = \{s_i \in \mathbf{z}_k : n_i \in N\}$
2. For each $k \in \{0, ..., p-1\}$ and $j = k-1 \pmod p$, either
   (a) $b_k = b_j$ or **(b)** $b_k = b_j + 1 \pmod q$
3. Given $k \in \{0, ..., p-1\}$ and $j = k-1 \pmod p$, **if** $b_k \neq b_j$ **then** $\mathbf{x}^N_{b_k} \neq \mathbf{x}^N_{b_j}$
4. For each $a \in \{0, ..., q-1\}$, $\exists$ $k \in \{0, ..., p-1\}$ such that $b_k = a$
5. There is no integer $q' \mid q$ $(q' < q)$ for which $\mathbf{x}_f^N = \mathbf{x}_g^N$ whenever f, g satisfies $f \pmod{q'} = g \pmod{q'}$

PROOF:

Case: $\Longrightarrow$

Suppose P occurs in A. Then, properties 1 and 2 follow directly from Properties 1 and 2 of Definition 3.

We now show properties 3, 4 and 5 hold.

Property 3

We use proof by contradiction to show property 3. We show that if the property does **not** hold, it is possible to find a smaller partial state sequence $P' = \{\mathbf{y}_0^N, ..., \mathbf{y}_{q'-1}^N\}$ and integers c_0, ..., $c_{p-1} \in \{0, ..., q'-1\}$ $(q' < q)$ that satisfy properties 1 and 2 of Definition 3 (thus contradiction property 3 of Definition 3 and the fact that P occurs in A)

Suppose there exists $k' \in \{0, ..., p-1\}$ and $j' = k' - 1 \pmod p$ for which $b_{k'} \neq b_{j'}$ and $\mathbf{x}_{b_{k'}}^N = \mathbf{x}_{b_{j'}}^N$. Then, Property 2 implies there's an integer h for which

- $h = b_{j'} \in \{0, ..., q-1\}$
- $h + 1 \pmod q = b_{k'}$ (since $b_{k'} \neq b_{j'}$)
- $\mathbf{x}_h^N = \mathbf{x}_{h+1 \pmod q}^N$

Take h (above), $q' = q - 1$ and consider the partial state sequence $P' = \{\mathbf{y}_0^N, ..., \mathbf{y}_{q'-1}^N\}$ where

- $\mathbf{y}_i^N = \mathbf{x}_i^N$ for $i = 0, ..., h-1$ (if $h > 0$)
- $\mathbf{y}_i^N = \mathbf{x}_{i+1}^N$ for $i = h, ..., q'-1$ (if $h < q-1$)

Then, for $m = 0, ..., p-1$, let

(i) $c_m = b_m$, if $b_m < h$

(ii) $c_m = b_m$, if $b_m = h < q' = q - 1$

(iii) $c_m = 0$, if $b_m = h = q' = q - 1$

(iv) $c_m = b_m$ - 1, if $b_m > h$

(Note: Since each $b_m \in \{0, ..., q-1\}$ and $h \in \{0, ..., q-1\}$, each $c_m \in \{0, ..., q-2\} = \{0, ..., q'-1\}$)

Now, from Property 2, for each $k \in \{0, ..., p-1\}$ and $j = k - 1 \pmod p$ either

(a) $b_j = b_k$

(b) $b_j = b_k - 1$ (when (a) is not true and $b_k > b_j \geq 0$)

(c) $b_j = q - 1$ (when (a) is not true and $b_k = 0$)

Considering all relevant cases (i) - (iv) and (a) - (c), we can show that $P' = \{\mathbf{y}_0^N, ..., \mathbf{y}_{q'-1}^N\}$ satisfies properties 1 and 2 of Definition 3.

i.e. The following are true for c_0, ..., $c_{p-1} \in \{0, ..., q'-1\}$ (defined above)

1. For $k = 0, ..., p-1$, $\mathbf{y}_{c_k}^N = \{s_i \in \mathbf{z}_k : n_i \in N\}$. This is because of the following
   - **(i)** If $b_k < h \leq q-1$, then
     $\mathbf{y}_{c_k}^N = \mathbf{y}_{b_k}^N = \mathbf{x}_{b_k}^N = \{s_i \in \mathbf{z}_k : n_i \in N\}$
   - **(ii)** If $b_k = h < q' = q-1$, then
     $\mathbf{y}_{c_k}^N = \mathbf{y}_{b_k}^N = \mathbf{y}_h^N = \mathbf{x}_{h+1}^N = \mathbf{x}_h^N = \mathbf{x}_{b_k}^N = \{s_i \in \mathbf{z}_k : n_i \in N\}$
   - **(iii)** If $b_k = h = q' = q-1$, then since $h+1 \pmod q = 0$
     $\mathbf{y}_{c_k}^N = \mathbf{y}_0^N = \mathbf{x}_0^N = \mathbf{x}_{h+1 \pmod q}^N = \mathbf{x}_h^N = \mathbf{x}_{b_k}^N = \{s_i \in \mathbf{z}_k : n_i \in N\}$
   - **(iv)** If $b_k > h$, then
     $\mathbf{y}_{c_k}^N = \mathbf{y}_{b_k-1}^N = \mathbf{x}_{b_k}^N = \{s_i \in \mathbf{z}_k : n_i \in N\}$
2. For each $k \in \{0, ..., p-1\}$ and $j = k-1 \pmod p$, either (a) $c_k = c_j$ or (b) $c_k = c_j + 1 \pmod{q'}$. This is because of the following
   - **(i)** If $b_k < h \leq q-1$, then either
     - **(a)** $c_k = b_k = b_j = c_j$
     - **(b)** $c_k = b_k = b_j + 1 \pmod{q'} = c_j + 1 \pmod{q'}$
       (since $b_j \geq 0$ and $b_j < h \leq q' = q-1$)
     - **(c)** $c_k = b_k = 0 = q-1 \pmod{q'} = b_j \pmod{q'} =$ either c_j or $c_j + 1 \pmod{q'}$
       (depending on whether or not $b_j = h$ or $b_j > h$)
   - **(ii and iii)** If $b_k = h \leq q-1$, then since $c_k = b_k \pmod{q'}$, either
     - **(a)** $c_k = b_k \pmod{q'} = b_j \pmod{q'} = c_j$
       (since $b_j = b_k = h$)
     - **(b)** $c_k = b_k \pmod{q'} = b_j + 1 \pmod{q'} = c_j + 1 \pmod{q'}$
       (since $b_j < h$ and $b_j \geq 0$)
     - **(c)** $c_k = b_k \pmod{q'} = 0 = q-1 \pmod{q'} = b_j \pmod{q'} =$ either c_j or $c_j + 1 \pmod{q'}$
       (depending on whether or not $b_j = h$ or $b_j > h$)
   - **(iv)** If $b_k > h$, then either
     - **(a)** $c_k = b_k - 1 = b_j - 1 = c_j$
       (since $b_j = b_k > h$)
     - **(b)** $c_k = b_k - 1 = b_j = b_j \pmod{q'} =$ either c_j or $c_j + 1 \pmod{q'}$
       (depending on whether or not $b_j = h$ or $b_j > h$)
     - **(c)** Case not possible

Property 4

Now, from Property 2, for each $k \in \{0, ..., p-1\}$ and $j = k-1 \pmod p$ either

(a) $b_k = b_j$

(b) $b_k = b_j + 1 \pmod q$ and $q > 0$

Let c be the number of times that $b_k \neq b_j$ when $k \in \{1, ..., p-1\}$ (i.e. c is number of times we get case (b))

Then, $b_{p-1} = b_0 + c \pmod q$.

Therefore, since either (a) or (b) must be true for $k = 0$ and $j = p - 1$, either

(c) $c = 0$ and $b_0 = ... = b_{p-1}$

(d) $c = eq - 1$ for some positive integer e $(q > 0)$

(e) $c = eq$ for some positive integer e $(q > 0)$

In cases (d) and (e), Property 4 must hold because b_k increases by either 0 or 1 $\pmod q$ as k increases (from 0 to $p-1$). This would imply that every $a \in \{0, ..., q-1\}$ occurs at least once.

In case (c), properties 1 and 2 of Definition 3 would hold for the partial state sequence $P' = \{\mathbf{y}_0^N\} = \{\mathbf{x}_{b_0}^N\}$. Therefore, since $P = \{\mathbf{x}_0^N, ..., \mathbf{x}_{q-1}^N\}$ occurs in A, it must be the case that $q = 1$ (Otherwise property 3 of Definition 3 would fail, using P' and $b_0 = ... = b_{p-1} = 0$). Property 4 would then follow, since for each $a \in \{0\}$, $b_0 = ... = b_{p-1} = 0$.

Property 5

We use proof by contradiction to show property 5. We show that if the property does **not** hold, it is possible to find a smaller partial state sequence $P' = \{\mathbf{y}_0^N, ..., \mathbf{y}_{q'-1}^N\}$ and integers c_0, ..., $c_{p-1} \in \{0, ..., q'-1\}$ $(q' < q)$ that satisfy properties 1 and 2 of Definition 3 (thus contradiction property 3 of Definition 3 and the fact that P occurs in A)

Suppose there is an integer $q' \mid q$ $(q' < q)$ for which $\mathbf{x}_f^N = \mathbf{x}_g^N$ whenever $f \pmod{q'} = g \pmod{q'}$.

Let,

- $P' = \{\mathbf{x}_0^N, ..., \mathbf{x}_{q'-1}^N\}$ (where each $\mathbf{x}_i^N$ is taken from P)
- $c_i = b_i \pmod{q'}$, for $i = 0, ..., p-1$

Then, properties 1 and 2 of Definition 3 are true for $P' = \{\mathbf{y}_0^N, ..., \mathbf{y}_{q'-1}^N\}$ and integers c_0, ..., $c_{p-1} \in \{0, ..., q'-1\}$ (defined above). This is because

1. For $k = 0, ..., p-1$, $\mathbf{x}_{c_k}^N = \mathbf{x}_{b_k \pmod{q'}}^N = \mathbf{x}_{b_k}^N = \{s_i \in \mathbf{z}_k : n_i \in N\}$
2. For each $k \in \{0, ..., p-1\}$ and $j = k - 1 \pmod p$, either
   (a) $c_k = b_k \pmod{q'} = b_j \pmod{q'} = c_j$
   (a) $c_k = b_k \pmod{q'} = (b_j + 1 \pmod q) \pmod{q'} = b_j + 1 \pmod{q'} = c_j + 1 \pmod{q'}$ (since $q' \mid q$)

Case: $\Longleftarrow$

Suppose properties 1 - 5 are true for $P = \{\mathbf{x}_0^N, \mathbf{x}_1^N, ..., \mathbf{x}_{q-1}^N\}$ and some sequence of integers b_0, ..., $b_{p-1} \in \{0, ..., q-1\}$

Then, we want to show that $P = \{\mathbf{x}_0^N, \mathbf{x}_1^N, ..., \mathbf{x}_{q-1}^N\}$ occurs in A (i.e. the 3 properties of Definition 3 are satisfied). Properties 1 and 2 follow directly from Properties 1 and 2 of this Lemma. Therefore, it just remains to show property 3 of Definition 3.

If property 3 were not true, it would be possible to find a smaller partial state sequence $P' = \{\mathbf{y}_0^N, \mathbf{y}_1^N, ..., \mathbf{y}_{q'-1}^N\}$ and integers c_0, ..., $c_{p-1} \in \{0, ..., q'-1\}$ ($q' < q$) that also satisfied properties 1 and 2 (of this Lemma and Definition 3)

Let $P' = \{\mathbf{y}_0^N, ..., \mathbf{y}_{q'-1}^N\}$ be the smallest such sequence (in terms of number of partial states q').

Then P' occurs in A and part $\Longrightarrow$ of this Lemma would ensure Properties 1 - 5 of this Lemma hold (for P').

Therefore, by Lemma S1.22, there is an integer h (highest common factor of q and q') for which

- $h \leq q' < q$
- $h \mid q$
- $\mathbf{x}_f^N = \mathbf{x}_g^N$ whenever $f \pmod h = g \pmod h$ (in P)

Therefore, if property 3 of Definition 3 were not true for P, property 5 of the Lemma would fail.

Therefore, property 3 of Definition 3 must be true and P must occur in A.

□

Lemma S1.24. Consider two partial state sequence $P_x = \{\mathbf{x}_0^N, ..., \mathbf{x}_{q-1}^N\}$ and $P_y = \{\mathbf{y}_0^M, ..., \mathbf{y}_{r-1}^M\}$,where $M \supseteq N$

P_x occurs in P_y (i.e. Definition S1.5 is satisfied)
$\Longleftrightarrow$
The following are true for $P_x = \{\mathbf{x}_0^N, ..., \mathbf{x}_{q-1}^N\}$ and some sequence of integers b_0, ..., $b_{r-1} \in \{0, ..., q-1\}$

1. For $k = 0, ..., r-1$, $\quad \mathbf{x}_{b_k}^N = \{s_i \in \mathbf{y}_k^M : n_i \in N \ (\subseteq M)\}$
2. For each $k \in \{0, ..., r-1\}$ and $j = k-1 \pmod r$, either **(a)** $b_k = b_j$ or **(b)** $b_k = b_j + 1 \pmod q$
3. Given $k \in \{0, ..., r-1\}$ and $j = k-1 \pmod r$, **if** $b_k \neq b_j$ **then** $\mathbf{x}_{b_k}^N \neq \mathbf{x}_{b_j}^N$
4. For each $a \in \{0, ..., r-1\}$, $\exists \ k \in \{0, ..., r-1\}$ such that $b_k = a$
5. There is no integer $q' \mid q$ $(q' < q)$ for which $\mathbf{x}_f^N = \mathbf{x}_g^N$ whenever f, g satisfies $f \pmod{q'} = g \pmod{q'}$

PROOF:

This Lemma can be proved in an analogous way to Lemma S1.23.

In particular, in the proof of Lemma S1.23,

- replace P by P_x
- replace A by P_y,
- replace $\mathbf{z}_k$ by $\mathbf{y}_k^M$
- replace p by r
- replace Definition 3 by Definition S1.5

and note that similar changes can also be made to adapt Lemmas S1.21 and S1.22.

□

Lemma S1.25. Consider a node set N and attractor $A = \{\mathbf{z}_0, \mathbf{z}_1, ..., \mathbf{z}_{p-1}\}$.

After applying Procedure S1.3 to N and A, we get a partial state sequence $P = \{\mathbf{x}_0^N, ..., \mathbf{x}_{q-1}^N\}$ and sequence of integers $b_0, ..., b_{p-1} \in \{0, ..., q-1\}$ for which the following are true

1. For $k = 0, ..., p-1$, $\mathbf{x}_{b_k}^N = \{s_i \in \mathbf{z}_k : n_i \in N\}$
2. For each $k \in \{0, ..., p-1\}$ and $j = k-1 \pmod p$, either **(a)** $b_k = b_j$ or **(b)** $b_k = b_j + 1 \pmod q$
3. Given $k \in \{0, ..., p-1\}$ and $j = k-1 \pmod p$, **if** $b_k \neq b_j$ **then** $\mathbf{x}_{b_k}^N \neq \mathbf{x}_{b_j}^N$
4. For each $a \in \{0, ..., q-1\}$, $\exists$ $k \in \{0, ..., p-1\}$ such that $b_k = a$
5. There is no integer $q' \mid q$ $(q' < q)$ for which $\mathbf{x}_f^N = \mathbf{x}_g^N$ whenever f, g satisfies $f \pmod{q'} = g \pmod{q'}$

PROOF

First note that $b_0, ..., b_{p-1} \in \{0, ..., q-1\}$ by Step 8. Also note that, once b_k has been specified during the procedure, it can only be altered in Step 8.

We now show the 5 properties of above are satisfied for P, A and $b_0, ..., b_{p-1}$ (defined in the procedure itself). For properties 1 - 3, we consider the cases $k = 0$ and $k > 0$ separately

Properties 1 -3 : Case $k = 0$

Property 1

The initialisation ensures

A: $b_0 = 0$

B: $\mathbf{x}^N_{b_0} = \{s_i \in \mathbf{z}_0 : n_i \in N\}$.

(Note: $b_0 = 0$ is not altered in Step 8, since $b_0 = 0$ and $q \geq 1$).

Therefore, Property 1 is satisfied

Properties 2 and 3

When $k = 0$, $\quad j = k - 1 \pmod p = p - 1 \quad$ and $\quad b_k = b_0 = 0$ (by **A**).

Then, because of Steps 1 and 6, one of the following is true by the end of Step 6

(a) $q^* = 1, \quad b_{p-1} = b_j = 0 \quad$ **and** $\quad \mathbf{x}^N_0 = \mathbf{x}^N_{b_0} = \mathbf{x}^N_{b_k} = \mathbf{x}^N_{b_j}$

(b) $q^* = b_{p-1} = b_j$ **and** $\mathbf{x}^N_0 = \mathbf{x}^N_{b_0} = \mathbf{x}^N_{b_k} = \mathbf{x}^N_{b_j}$

(c) $q^* = b_{p-1} + 1 = b_j + 1$ **and** $\mathbf{x}^N_0 = \mathbf{x}^N_{b_0} = \mathbf{x}^N_{b_k} \neq \mathbf{x}^N_{b_j}$

Then, since $q \mid q^*$ ($q^* = aq$ for some positive integer a) in Step 7, Step 7 implies either

(a) $b_j = 0$ **and** $\mathbf{x}^N_0 = \mathbf{x}^N_{b_k} = \mathbf{x}^N_{b_j}$

(b) $b_j \pmod q = 0$ **and** $\mathbf{x}^N_0 = \mathbf{x}^N_{b_k} = \mathbf{x}^N_{b_j}$

(c) $b_j \pmod q = q - 1$ **and** $\mathbf{x}^N_0 = \mathbf{x}^N_{b_k} \neq \mathbf{x}^N_{b_j} = \mathbf{x}^N_{q-1}$

Therefore, since $b_k = b_0 = 0$ (by **A**), the following are true at the end of Step 8

(a) $b_k = b_j = 0$ **and** $\mathbf{x}^N_{b_k} = \mathbf{x}^N_{b_j} = \mathbf{x}^N_0$

(b) $b_k = b_j = 0$ **and** $\mathbf{x}^N_{b_k} = \mathbf{x}^N_{b_j} = \mathbf{x}^N_0$

(c) $b_k = b_j + 1 \pmod q$ **and** $\mathbf{x}^N_{b_k} = \mathbf{x}^N_0 \neq \mathbf{x}^N_{q-1} = \mathbf{x}^N_{b_j}$

Because of (a), (b) and (c) above, Property 2 is satisfied.

Property 3 is satisfied because **if** $b_k \neq b_j$, **then** (c) above must be true and $\mathbf{x}^N_{b_k} \neq \mathbf{x}^N_{b_j}$ (as required)

Properties 1 -3 : Case k = 1, ..., $p-1$ (and $p > 1$)

When $p > 1$, Step 3 is applied for every $k = 1, ..., p-1$.

Consider any $k \in \{1, ..., p-1\}$.

Property 1

If the condition in Step 3 is satisfied, $\mathbf{x}_{b_k}^N = \{s_i \in \mathbf{z}_k : n_i \in N\}$ is true after Step 3. If the condition is not satisfied, $\mathbf{x}_{b_k}^N = \{s_i \in \mathbf{z}_k : n_i \in N\}$ is true after Step 5.

As mentioned earlier, b_k can then only be altered in Step 8, when b_k is replaced by $b_k \pmod q$. However, because of Step 7b, $\mathbf{x}_{b_k}^N = \{s_i \in \mathbf{z}_k : n_i \in N\}$ would remain unaltered.

Therefore Property 1 is satisfied.

Properties 2 and 3

Consider any $k \in \{1, ..., p-1\}$ and $j = k-1 \pmod p$ (where $p > 1$)

From steps 2, 3, 4 and 5, either (a) or (b) are true depending on whether or not the condition in Step 3 is satisfied

(a) $b_k = b_j$ **and** $\mathbf{x}_{b_k}^N = \mathbf{x}_{b_j}^N$

(b) $b_k = b_j + 1$ **and** $\mathbf{x}_{b_k}^N \neq \mathbf{x}_{b_j}^N$

Now let $f_k = b_k \pmod q$ and $f_j = b_j \pmod q$, where $q \mid q^*$ is the integer identified in Step 7. Then, after Step 7, either

(a) $f_k = f_j$ **and** $\mathbf{x}_{f_k}^N = \mathbf{x}_{b_k}^N = \mathbf{x}_{b_j}^N = \mathbf{x}_{f_j}^N$

(b) $f_k = f_j + 1 \pmod q$ **and** $\mathbf{x}_{f_k}^N = \mathbf{x}_{b_k}^N \neq \mathbf{x}_{b_j}^N = \mathbf{x}_{f_j}^N$

Therefore after Step 8, either of the following are true (as required)

(a) $b_k = b_j$ **and** $\mathbf{x}_{b_k}^N = \mathbf{x}_{f_k}^N = \mathbf{x}_{f_j}^N = \mathbf{x}_{b_j}^N$

(b) $b_k = b_j + 1 \pmod q$ **and** $\mathbf{x}_{b_k}^N = \mathbf{x}_{f_k}^N \neq \mathbf{x}_{f_j}^N = \mathbf{x}_{b_j}^N$

Because of (a) and (b) above, Property 2 is satisfied.

Property 3 is satisfied because **if** $b_k \neq b_j$, **then** (b) above must be true and $\mathbf{x}_{b_k}^N \neq \mathbf{x}_{b_j}^N$ (as required)

Property 4

Initially, $b_0 = 0$.

Then, for each $k \in \{1, ..., p-1\}$ and $j = k-1$, Steps 2 - 5 imply that at the end of Step 7 either

(a) $b_k = b_j$

(b) $b_k = b_j + 1$

Therefore, as k increases from 0 to $p-1$, b_k is increases in intervals of 1.

Moreover, since $q \leq q^* \leq b_{p-1} + 1$ (from Steps 1, 6 and 7), there exists b_0, ..., b_d that satisfy the following at the end of Step 7

(i) For $i = 0, ..., d, \quad b_i \in \{0, ..., q-1\}$

(ii) For each $a \in \{0, ..., q-1\}, \quad \exists \quad k \in \{0, ..., d\}$ such that $b_k = a$

Moreover, after Step 8, b_0, ..., b_d remain unchanged (because of (i)).

Therefore, property 4 is satisfied

Property 5

In Step 7, $q \quad (q \mid q^*)$ is chosen to be the smallest integer for which $\mathbf{x}_f^N = \mathbf{x}_g^N$ whenever $f \pmod q = g \pmod q$

If there were a smaller $q' < q, \quad (q' \mid q)$ for which $\mathbf{x}_f^N = \mathbf{x}_g^N$ whenever $f \pmod{q'} = g \pmod{q'}$, then it would have been identified in Step 7 (since it is also the case that $q' \mid q^*$)

□
