# Supplementary material for: Identifying dynamical modules from genetic regulatory systems: applications to the segment polarity network
Source: BMC Bioinformatics. 2007 Oct 25;8:413. doi: 10.1186/1471-2105-8-413 (PMC2233651; doi:10.1186/1471-2105-8-413)
Supplement: Additional file 2 — Description of the procedures used to identify interactions between subsystems, along with formal proofs. This is given as a pdf file and is referred to in the main text. [file 1471-2105-8-413-S2.pdf]

Supporting Text 2:
Interactions between subsystems

Given an individual subsystem S_y, we are interested in which subsystems regulate it and ensure its occurrence in an attractor. Essentially, we want to find collections of subsystems $\mathbb{S}_x = \{S_{x_1}$, ..., $S_{x_f}\}$ whose co-occurrence in an attractor *triggers* a chain of interactions that results in the occurrence of S_y.

In Sections S2.1 and S2.2, we look at Boolean network models to see how the model's Boolean functions can be used to identify such interactions. Sections S2.1 looks at how partial states are regulated within attractors. Sections S2.2 then transfers these ideas back to subsystems. Some simple examples to explain subsystems and interactions between them are given in *Supporting Text 3* (in Section S3.3). Even without the Boolean functions, it is possible to identify relationships between subsystems. Sections S2.3 describes one such approach.

This supporting text is a more formal description / proof of the procedures given in the main manuscript. The procedures in the main manuscript are revisited here in the following sections.

Procedure 6: Corresponds to Procedures S2.8, S2.11 and Theorem S2.12 in Section S2.1

Procedure 7: Corresponds to Procedure S2.19 and Theorem S2.20 in Section S2.2.2

First we introduce / repeat a few definitions used throughout this section

Definition S2.1. A *network state*, $\mathbf{z} = (s_1, ..., s_v) \in \{0,1\}^v$ is a set of v Boolean states, one for each node $n_i \in V$ (where V is the set of all nodes).

Definition S2.2. An *attractor state*, $\mathbf{z} = (s_1, ..., s_v) \in \{0,1\}^v$ is a network state that occurs in some attractor $A \in \{\mathbf{z}_0, ..., \mathbf{z}_{p-1}\}$

Definition S2.3. A *partial state*, $\mathbf{x}^N \in \{0,1\}^{|N|}$ is a set of $\mid N \mid$ Boolean states, one for each node $n_i \in N (\subseteq V)$. i.e. $\mathbf{x}^N = \{s_i : n_i \in N\}$

Definition S2.4. A partial state $\mathbf{x}^N$ is *contained* in another partial state $\mathbf{z}^P$ if $N \subseteq P$ and each node $n_i \in N$ has the same Boolean state (s_i) in both $\mathbf{x}^N$ and $\mathbf{z}^P$. We can also say $\mathbf{z}^P$ *contains* $\mathbf{x}^N$.

This definition is also applicable to a network / attractor state $\mathbf{z}$, by letting $P = V$ and $\mathbf{z}^P = \mathbf{z}$.

S2.1 Regulation of partial states in attractors

As the Boolean network model progresses over time, the Boolean functions $\mathbf{f} = (f_1$, ..., $f_v)$ determine how each node is updated from one time step to the next. i.e. Given a network state $\mathbf{x} = \mathbf{x}(t)$ at time t, the model progresses from one time step to the next as follows

- $\mathbf{x}(t+1) = \mathbf{f}(\mathbf{x}(t)) = (f_1(\mathbf{x}(t))$, ..., $f_v(\mathbf{x}(t)))$

It may be that a partial state $\mathbf{x}^N$ controls the Boolean functions $\{f_i : n_i \in M\}$ and ensures the occurrence of $\mathbf{y}^M$ in the following time step. i.e $\mathbf{x}^N$ is a *predecessor* of $\mathbf{y}^M$ (or $\mathbf{x}^N$ *triggers the occurrence* of $\mathbf{y}^M$).

Definition S2.5. A partial state $\mathbf{x}^N$ is a *predecessor* of another partial state $\mathbf{y}^M$ if the following holds

- If a network state $\mathbf{z}$ contains $\mathbf{x}^N$, then $\mathbf{f}(\mathbf{z})$ contains $\mathbf{y}^M$

This definition is also applicable to a network / attractor state $\mathbf{z}$, by letting $N = V$ and $\mathbf{x}^N = \mathbf{z}$.

In the main manuscript, we also say that $\mathbf{x}^N$ *triggers the occurrence* of $\mathbf{y}^M$. However, in this Supporting Text, we keep to the terminology *predecessor*.

The following results relate to predecessors and are utilised during later analyses (to improve efficiency).

Lemma S2.6. Suppose, a partial state $\mathbf{x}^N$ is a predecessor of another partial state $\mathbf{y}^M$

If a partial state $\mathbf{z}^P$ contains $\mathbf{x}^N$, then $\mathbf{z}^P$ is a predecessor of $\mathbf{y}^M$

PROOF: See Section S2.4

Lemma S2.7. Consider two partial states $\mathbf{y}_1^{M_1}$, $\mathbf{y}_2^{M_2}$ where $\mathbf{y}_2^{M_2}$ contains $\mathbf{y}_1^{M_1}$.

Then, if $\mathbf{x}^N$ is a predecessor of $\mathbf{y}_2^{M_2}$, $\mathbf{x}^N$ is also a predecessor of $\mathbf{y}_1^{M_1}$

PROOF: See Section S2.4

Given a partial state $\mathbf{y}^M$, its predecessors can be found by using the approach in Irons (2006). The results of this procedure are given below, whilst the precise details can be found in the original paper

Procedure S2.8. Given a partial state $\mathbf{y}^M$, the procedure *FindPredecessors*($\mathbf{y}^M$) in Irons (2006) (Appendix B.2.2) identifies partial states $\mathbf{x}_1^{N_1}$, ..., $\mathbf{x}_r^{N_r}$ for which the following are true

1. For $i = 1, ..., r$, $\quad \mathbf{x}_i^{N_i}$ is a predecessor of $\mathbf{y}^M$.
2. For $i = 1, ..., r$, $\quad \mathbf{x}_i^{N_i}$ does not contain any partial state $\mathbf{x}_j^{N_j}$ ($j \in \{1, ..., r\}$, $j \neq i$)
3. If an attractor state $\mathbf{z}$ is a predecessor of $\mathbf{y}^M$, then $\mathbf{z}$ contains $\mathbf{x}_i^{N_i}$ for some $i \in \{1, ..., r\}$

Property 2 ensures that only the most informative predecessors are kept (see Lemma S2.6). The procedure can also be modified so that property 3 is more general and applies to network states (rather than attractor states). This version is not necessary here.

Given a partial state $\mathbf{y}^M$ that occurs in an attractor state $\mathbf{z}_a \in A$, this idea of predecessors can be extended to look at the regulation of $\mathbf{y}^M$ in the attractor A

Definition S2.9. Consider an attractor $A = \{\mathbf{z}_0, ..., \mathbf{z}_{p-1}\}$ and two partial states $\mathbf{x}^N$ and $\mathbf{y}^M$

$\mathbf{x}^N$ is a *k-predecessor* of $\mathbf{y}^M$, in an attractor state $\mathbf{z}_a \in A$ if

1. $\mathbf{x}^N$ and $\mathbf{y}^M$ are both contained in the attractor state $\mathbf{z}_a$
2. There exists a sequence of partial states $\mathbf{z}_0^{P_0}$, $\mathbf{z}_1^{P_1}$, ..., $\mathbf{z}_k^{P_k}$ for which the following are true
   (a) $k = cp$, where c is a positive integer.
   (b) $P_0 = N$ and $\mathbf{z}_0^{P_0} = \mathbf{x}^N$
   (c) $P_k = M$ and $\mathbf{z}_k^{P_k} = \mathbf{y}^M$
   (d) For $i = 0, ..., k-1$, $\mathbf{z}_i^{P_i}$ is a predecessor of $\mathbf{z}_{i+1}^{P_{i+1}}$
   (e) For $i = 0, ..., k$, $\mathbf{z}_i^{P_i}$ is contained in the attractor state $\mathbf{z}_b \in A$ (where $b = a + i \pmod{p}$)

Essentially, in this definition, if $\mathbf{x}^N$ is contained $\mathbf{z}_a \in A$, then it ensures that $\mathbf{y}^M$ is also contained in $\mathbf{z}_a \in A$, $k = cp$ time steps later. Moreover, this process can be traced through the attractor A at each time step (because of 2(e)).

Once we have found a k-predecessor $\mathbf{x}^N$, the following result implies that all larger partial states $\mathbf{z}^P$ (containing $\mathbf{x}^N$ and contained in $\mathbf{z}_a$) are also k-predecessors of $\mathbf{y}^M$.

Lemma S2.10. Suppose, $\mathbf{x}^N$ is a k-predecessor of $\mathbf{y}^M$ in $\mathbf{z}_a \in A$.

If a partial state $\mathbf{z}^P$ contains $\mathbf{x}^N$ and is contained in $\mathbf{z}_a$, then $\mathbf{z}^P$ is a k-predecessor of $\mathbf{y}^M$ in $\mathbf{z}_a \in A$.

PROOF: see Section S2.4

Given a partial state $\mathbf{y}^M$ and attractor state $\mathbf{z}_a \in A = \{\mathbf{z}_0, ..., \mathbf{z}_{p-1}\}$, the following procedure identifies partial states $\mathbf{x}^N$ that are *k-predecessors* of $\mathbf{y}^M$ in $\mathbf{z}_a \in A$

Procedure S2.11. (Procedure 6 from main manuscript)

In this procedure, we take as an input a partial state $\mathbf{y}^M$ that occurs in an attractor state $\mathbf{z}_a \in A = \{\mathbf{z}_0, ..., \mathbf{z}_{p-1}\}$.

Initially, let sets $\mathbf{U} = \varnothing$, $\mathbf{W}_0 = \{\mathbf{y}^M\}$ and $\mathbf{W}_i = \varnothing$ for all $i \geq 1$. Moreover, set $t = 0$. The procedure then enters the following loop

Step 1 :
Execute Procedure S2.8 for every partial state $\mathbf{z}^P \in \mathbf{W}_t$. For each newly identified predecessor $\mathbf{x}_i^{N_i}$, of $\mathbf{z}^P$, $\mathbf{x}_i^{N_i}$ is added to $\mathbf{W}_{t+1}$

Step 2 :
(a) Let $t = t + 1$ (increment t by 1)
(b) Let $t' = a - t \pmod{p}$

Step 3 :
Remove all partial states from $\mathbf{W}_t$ that contain a *different* partial state in $\mathbf{W}_t$

Step 4 :
Remove all partial states from $\mathbf{W}_t$ that are *not* contained in the attractor state $\mathbf{z}_{t'}$

Step 5 :
If $t \pmod p = 0$, go to Step 6. Otherwise go back to Step 1.

Step 6 :
For every partial state $\mathbf{x}^N \in \mathbf{W}_t$,
(a) Check whether $\mathbf{x}^N$ contains or equals any partial state $\mathbf{u}^L \in \mathbf{U}$.
(b) Check whether $\mathbf{x}^N$ is contained within any partial state $\mathbf{u}^L \in \mathbf{U}$.

If (a) is true then remove $\mathbf{x}^N$ from $\mathbf{W}_t$.

If (a) is false then add $\mathbf{x}^N$ to $\mathbf{U}$ and discard any $\mathbf{u}^L$ identified in (b).

Step 7 :
If $\mathbf{W}_t$ is empty, **end procedure**. Otherwise, return to Step 1.

At the end of this procedure, $\mathbf{U}$ contains suitable k-predecessors of $\mathbf{y}^M$ in $\mathbf{z}_a \in A$. Starting from $\mathbf{z}_a$, Steps 1, 2 and 4 imply the procedure goes backwards around the attractor, identifying predecessors within the relevant attractor state. Once we return to $\mathbf{z}_a$ ($t \pmod p = 0$), we store any newly identified k-predecessors of $\mathbf{y}^M$ in $\mathbf{z}_a \in A$ (in the set $\mathbf{U}$). Steps 3, 4 and 6 ensure we only store / analyse the most informative and suitable k-predecessors, thus speeding up the analysis. Because of Lemma S2.7, there is no advantage in considering partial states in W_t, that contain other partial states in W_t. Because of Lemma S2.10, we need only identify k-predecessors that do **not** contain other k-predecessors.

The Theorem below shows that the set $\mathbf{U}$ contains suitable k-predecessors of $\mathbf{y}^M$ in A, at the end of the procedure.

Theorem S2.12. At the end of Procedure S2.11

1. The set $\mathbf{U}$ is non-empty
2. If a partial state $\mathbf{x}^N$ is contained in $\mathbf{U}$ at the end of the procedure, then
   (a) $\mathbf{x}^N$ is a k-predecessor of $\mathbf{y}^M$ in $\mathbf{z}_a \in A$
   (b) $\mathbf{x}^N$ contains no other partial states in $\mathbf{U}$

PROOF: see Section S2.4

S2.2 Regulation of subsystems

The definitions and procedures from Section S2.1 can be used to describe how subsystems are regulated in attractors. However, since partial states within subsystems could be subject to different time lags in different attractors (see Definition 3 (main text) and Section S3.1 in *Supporting Text 3*), we need to consider the precise dynamics (the *instances*) of subsystems in attractors. i.e.

Definition S2.13. (Definition 9 from main manuscript)

Consider a collection of subsystems $\mathbb{S} = \{S_1, ..., S_f\}$ where every $S_i = \{\mathbf{x}_{i_0}^{N_i}, ..., \mathbf{x}_{i_{q_i}}^{N_i}\} \in \mathbb{S}$ involves a node set N_i and occurs in the attractor $A = \{\mathbf{z}_0, ..., \mathbf{z}_{p-1}\}$

The *instance* of $\mathbb{S}$ in A is the partial state sequence $\mathbf{z}_0^M, ..., \mathbf{z}_{p-1}^M$, where

1. $M = N_1 \cup ... \cup N_f$
2. For $k = 0, ..., p-1, \quad \mathbf{z}_k^M = \{s_x \in \mathbf{z}_k : n_x \in M\}$.

In order to describe how a subsystem S_y is regulated, we want to see which collections of subsystems $\mathbb{S}_x = \{S_{x_1}, ..., S_{x_f}\}$ can set of a chain of interactions and *trigger* the occurrence of S_y in an attractor. Using these instances, we can come up with the following definition

Definition S2.14. (Definition 10 from main manuscript)

Suppose we have

1. An attractor $A = \{\mathbf{z}_0, ..., \mathbf{z}_{p-1}\}$
2. A collection of subsystems $\mathbb{S}_x = \{S_{x_1}, ..., S_{x_f}\}$ where
   (a) $S_{x_1}, ..., S_{x_f}$ all occur in A
   (b) $\mathbf{x}_0^N, ..., \mathbf{x}_{p-1}^N$ is the instance of $\mathbb{S}_x$ in A
3. An individual subsystem S_y where
   (a) S_y occurs in A
   (b) $\mathbf{y}_0^M, ..., \mathbf{y}_{p-1}^M$ is the instance of S_y in A

Then $\mathbb{S}_x$ *triggers* S_y in A if the following holds for every $i \in \{0, ..., p-1\}$

- $\mathbf{x}_i^N$ is a k_i-predecessor of $\mathbf{y}_i^M$ in $\mathbf{z}_i \in A$ (for some k_i)

This definition can easily be adapted to consider whether a collection of subsystems $\mathbb{S}_x = \{S_{x_1}, ..., S_{x_f}\}$ *triggers* another collection of subsystem $\mathbb{S}_y = \{S_{y_1}, ..., S_{y_g}\}$ in an attractor A.

Here, the occurrence of S_y in A can be explained by the occurrence of $\mathbb{S}_x$. If the subsystems $S_{x_1}, ..., S_{x_f}$ are established in the attractor A, $\mathbf{x}_0^N, ..., \mathbf{x}_{p-1}^N$ would occur over and over again. This can then (eventually) ensure the occurrence of $\mathbf{y}_0^M, ..., \mathbf{y}_{p-1}^M$ over and over again. In the model, it is unlikely that subsystems establish themselves one at a time. However, knowing which collections of subsystems *trigger* S_y indicate which parts of the system are primarily responsible for regulating S_y

Obviously, S_y may occur in multiple attractors and different collections of subsystems may be responsible for triggering S_y in different attractors. Therefore, in order to describe how S_y is regulated we want a more complete description

Definition S2.15. A set of subsystem collections $\mathbb{S}_1$, ..., $\mathbb{S}_g$ *regulates* an (individual) subsystem S_y if the following are true

1. For i = 1, ..., g, $\exists$ an attractor A for which $\mathbb{S}_i$ triggers S_y in A
2. If S_y occurs in an attractor A, $\exists$ $i \in \{1, .., g\}$ for which $\mathbb{S}_i$ triggers S_y in A

We call the set $\{\mathbb{S}_1, ..., \mathbb{S}_g\}$ a *regulation set* of S_y

The procedure in Section S2.2.2 shows one way of identifying such a regulation set, for a subsystem S_y. However, there may be some regulation sets that are more descriptive than others. Therefore, we first run through some extra constraints on $\mathbb{S}_1$, ..., $\mathbb{S}_g$

S2.2.1 Extra constraints

Lemma S2.16. Consider two collections of subsystems $\mathbb{S}_a$ and $\mathbb{S}_b$, and suppose

(a) $\mathbb{S}_a$ triggers an individual subsystem S_y in an attractor $A = \{\mathbf{z}_0, ..., \mathbf{z}_{p-1}\}$

(b) $\mathbb{S}_b \supset \mathbb{S}_a$

(c) Every subsystem $S \in \mathbb{S}_b$ occurs in A

Then, $\mathbb{S}_b$ triggers S_y in A

PROOF: see Section S2.4

For a regulation set $\{\mathbb{S}_1, ..., \mathbb{S}_g\}$ of S_y, there may be some redundancy.

Firstly, because of Lemma S2.16, if there exists $\mathbb{S}_a$, $\mathbb{S}_b$ (a, $b \in \{1, ..., r\}$, $a \neq b$) for which

(a) $\mathbb{S}_b \supset \mathbb{S}_a$

(b) If $\mathbb{S}_b$ triggers S_y in A, then $\mathbb{S}_a$ also triggers S_y in A

then the regulatory ability of $\mathbb{S}_b$ is just a result of the smaller set $\mathbb{S}_a$. Therefore, subsystem collections such as $\mathbb{S}_b$ (where (a) and (b) hold) are not counted as triggering S_y in A (in Procedure S2.19)

Secondly, properties 1 and 2 of Definition S2.15 could still hold after a subsystem collection $\mathbb{S}_i$ ($i \in \{1, ..., g\}$) is removed from the set $\{\mathbb{S}_1, ..., \mathbb{S}_g\}$. However, even in this scenario, it may be that each $\mathbb{S}_i$ is still informative. The following constraint is one way of deciding which subsystem collections to keep and which to remove (if any). This extra (optional) constraint was used in the examples in this paper. A description of how to apply this constraint is given in Section S2.2.2.

Definition S2.17. Consider a regulation set $\{\mathbb{S}_1, ..., \mathbb{S}_g\}$ of a subsystem S_y.

$\mathbb{S}_a$ ($a \in \{1, ..., g\}$) is *key* to $\{\mathbb{S}_1, ..., \mathbb{S}_g\}$ in an attractor A if

1. $\mathbb{S}_a$ triggers S_y in A
2. It is possible to find a collection of subsystems $\mathbb{T}$ for which
   (a) $\mathbb{T}$ triggers $\mathbb{S}_a$ in A
   (b) $\mathbb{T} \not\supseteq \mathbb{S}_b$ for any $b \neq a$ ($b \in \{1, ..., g\}$)

If $\mathbb{S}_a$ is not *key* to $\{\mathbb{S}_1, ..., \mathbb{S}_g\}$ in A, other collections of subsystems in the regulation set are necessary to ensure $\mathbb{S}_a$'s occurrence in A. Therefore, in this case, $\mathbb{S}_a$ is not counted as triggering A. In the rare occurrence that no subsystem collection is *key* to $\{\mathbb{S}_1, ..., \mathbb{S}_g\}$ for a particular attractor A (possibly because cyclic dependencies exist between collections of subsystems), this extra constraint would be ignored.

If $\mathbb{S}_a$ is not *key* to $\{\mathbb{S}_1, ..., \mathbb{S}_g\}$ in any attractor, it would be removed from the regulation set.

Definition S2.15 can be modified to take account of this extra constraint, as follows

Definition S2.18. A set of subsystem collections $\mathbb{S}_1, ..., \mathbb{S}_g$ *regulates* an (individual) subsystem S_y if the following are true

1. For $i = 1, ..., g$, $\exists$ an attractor A for which both of the following are true
   (a) $\mathbb{S}_i$ triggers S_y in A
   (b) $\mathbb{S}_i$ is *key* to $\{\mathbb{S}_1, ..., \mathbb{S}_g\}$ in A
2. If S_y occurs in an attractor A, $\exists$ $i \in \{1, .., g\}$ for which $\mathbb{S}_i$ triggers S_y in A

We call the set $\{\mathbb{S}_1, ..., \mathbb{S}_g\}$ the *regulation set* of S_y

S2.2.2 Procedure to identify regulation sets

Given a subsystem S_y (involving a node set M_y) and a set of attractors $\mathbb{C}_y$, where

(a) S_y occurs in every attractor $A \in \mathbb{C}_y$

(b) S_y does not occur in any attractor $A \notin \mathbb{C}_y$

The following procedure (Procedure S2.19) demonstrates a method of identifying a *regulation set* of S_y (see Definition S2.15 above)

At the start of the procedure, we assume we know every subsystem $S = \{\mathbf{x}_0^N, ..., \mathbf{x}_{q-1}^N\}$, along with the node set involved (N) and a list of attractors it occurs in. Each subsystem and node set N is a by-product of the method of identifying subsystems (described in *Supporting Text 1*). A list of attractors can be found by applying Procedure S1.9 (from *Supporting Text 1*) to each node set N. This information is used in Step 2 of the procedure (below).

Procedure S2.19. (Procedure 7 from main manuscript)

Initially, let the set $\mathbf{R} = \varnothing$ (empty set)

For every $A_i = \{\mathbf{z}_0, ..., \mathbf{z}_{p-1}\} \in \mathbb{C}_y$ carry out the following steps.

Step 1 :
Let the set $\mathbf{R}_i = \varnothing$. Let the sets $\mathbf{U}_0$, ..., $\mathbf{U}_{p-1} = \varnothing$

Step 2 :
Identify every subsystem T_1, ..., T_h that occurs in A_i. Moreover, let M_1, ..., M_h be the node sets involved in T_1, ..., T_h (resp)

Step 3 :
Identify the instance of S_y in A_i. i.e. $\mathbf{y}_0^{M_y}$, ...,$\mathbf{y}_{p-1}^{M_y}$.

(The procedure for this is obvious from Definition S2.13, given the node set M_y and attractor A_i)

Step 4 :
For $j = 0$, ..., $p-1$, carry out Procedure S2.11 to identify k_j-predecessors of $\mathbf{y}_j^{M_y}$ in $\mathbf{z}_j \in A_i$. The resulting k_j-predecessors are added to the set $\mathbf{U}_j$

Step 5 :
For every possible combination of partial states $\mathbf{x}_0^{N_0}$, ..., $\mathbf{x}_{p-1}^{N_{p-1}}$ satisfying

- $\mathbf{x}_j^{N_j} \in \mathbf{U}_j$ (for $j = 0$, ..., $p-1$)

carry out the following

(a) Let $N = N_0 \cup ... \cup N_{p-1}$
(b) Let $\mathbb{S} = \{T_a : M_a \cap N \neq \varnothing\}$
(c) Add $\mathbb{S}$ to the set $\mathbf{R}_i$

Step 6 :
Remove all subsystem collections $\mathbb{S}$ from $\mathbf{R}_i$ that contain other subsystem collections $\mathbb{S}' \in \mathbf{R}_i$. (i.e. $\mathbb{S} \supset \mathbb{S}'$)

Step 7 :
Add the subsystem collections in $\mathbf{R}_i$ to the set $\mathbf{R}$

At the end of this procedure, every subsystem collection $\mathbb{S} \in \mathbf{R}_i$ triggers S_y in the attractor A_i. $\mathbf{R}$ is then the regulation set of S_y. This is proved with the following Theorem

Theorem S2.20. At the end of Procedure S2.19

1. Whenever $A_i \in \mathbb{C}_y$,
   - **(a)** $\mathbf{R}_i$ is non-empty
   - **(b)** Every subsystem collection $\mathbb{S} \in \mathbf{R}_i$ triggers S_y in A_i
2. $\mathbf{R}$ is a regulation set of S_y

PROOF: see Section S2.4

Given a regulation set $\mathbf{R} = \{\mathbb{S}_1, ..., \mathbb{S}_g\}$ of S_y, we may want to apply the extra constraint in Definitions S2.17 and S2.18. Thus only keeping those subsystem collections $\mathbb{S}_a$ that are key to $\mathbf{R} = \{\mathbb{S}_1, ..., \mathbb{S}_g\}$ in some attractor A_i.

To do this we let $\mathbf{R}' = \mathbf{R}$ and $\mathbf{R}'_i = \mathbf{R}_i$ for each set in the above procedure. Then, we take $\mathbf{R}'$ and re-apply Procedure S2.19 to every collection of subsystems $\mathbb{S}_a \in \mathbf{R}$. As inputs to the procedure take $\mathbb{S}_a$ and the set of attractors $\mathbb{C}_a \cap \mathbb{C}_y$ as inputs, instead of S_y and $\mathbb{C}_y$. Here, $\mathbb{C}_a$ is the set of attractors for which every $S \in \mathbb{S}_a$ occurs.

For each $\mathbb{S}_a \in \mathbf{R}'$ and $A_i \in \mathbb{C}_a \cap \mathbb{C}_y$, this will give collections of subsystems $\mathbb{T}_1, ..., \mathbb{T}_s$ responsible for triggering $\mathbb{S}_a$ in A_i. This information can then be used to see if $\mathbb{S}_a$ is key to $\{\mathbb{S}_1, ..., \mathbb{S}_g\}$ in A_i (see Definition S2.17)

If $\mathbb{S}_a$ is not key to $\{\mathbb{S}_1, ..., \mathbb{S}_g\}$ in A_i, $\mathbb{S}_a$ is removed from $\mathbf{R}'_i$. In the rare occurrence that every subsystem collection in $\mathbf{R}'_i$ is not key to $\{\mathbb{S}_1, ..., \mathbb{S}_g\}$ in A_i, this extra constraint would be ignored.

After doing this for every $\mathbb{S}_a \in \mathbf{R}'$, $\mathbf{R}'$ can then be re-formed from all of the $\mathbf{R}'_i$'s (repeat Step 7).

Theorem S2.12 would still hold for the $\mathbf{R}'_i$'s and $\mathbf{R}'$ at the end of these extra steps. This is because none of the original $\mathbf{R}'_i$'s are emptied and no subsystem collections are added. Part 2 of the Theorem can be proved in the same way.

S2.3 Hierarchical links between subsystems

On a simple observational level, a subsystem S_x may be *hierarchically linked* to another subsystem S_y, because S_x only occurs in an attractor in conjunction with the $'$higher order$'$ S_y. Such hierarchical links can be identified without any prior knowledge of the underlying model. These links could potentially correspond to relationships between subsystem, that are worth studying more detail

Definition S2.21. Consider two subsystems S_x and S_y. S_x is *hierarchically linked* to S_y if the following are true

- S_x occurs in an attractor $A \Longrightarrow S_y$ occurs in an attractor A

Furthermore, such a link can be viewed as *direct* if there it is impossible to find a subsystem S_z for which the following is true

1. S_x is hierarchically linked to S_z
2. S_z is hierarchically linked to S_y
3. There exists attractors A_1 and A_2 for which
   - **(a)** S_y occurs in A_1 and A_2
   - **(b)** S_z occurs in A_1 but not A_2
   - **(c)** S_x occurs in neither A_1 nor A_2

This terminology can easily be extended to collections of subsystems.

S2.4 Proofs for earlier results

Here, we provide proofs for results introduced in this Supporting Text.

Lemma. S2.6

Suppose, a partial state $\mathbf{x}^N$ is a predecessor of another partial state $\mathbf{y}^M$

If a partial state $\mathbf{z}^P$ contains $\mathbf{x}^N$, then $\mathbf{z}^P$ is a predecessor of $\mathbf{y}^M$

PROOF:

We need to show $\mathbf{z}^P$ is a predecessor of $\mathbf{y}^M$ (i.e. Definition S2.5 is satisfied)

Suppose a network state $\mathbf{z}$ contains $\mathbf{z}^P$. Then $\mathbf{z}$ also contains $\mathbf{x}^N$ (since $\mathbf{z}^P$ contains $\mathbf{x}^N$). Therefore, since $\mathbf{x}^N$ is a predecessor of $\mathbf{y}^M$, Definition S2.5 implies $\mathbf{f}(\mathbf{z})$ contains $\mathbf{y}^M$.

Therefore, $\mathbf{z}^P$ is a predecessor of $\mathbf{y}^M$ (as required)

□

Lemma. S2.7

Consider two partial states $\mathbf{y}_1^{M_1}$, $\mathbf{y}_2^{M_2}$ where $\mathbf{y}_2^{M_2}$ contains $\mathbf{y}_1^{M_1}$.

Then, if $\mathbf{x}^N$ is a predecessor of $\mathbf{y}_2^{M_2}$, $\mathbf{x}^N$ is also a predecessor of $\mathbf{y}_1^{M_1}$

PROOF:

We need to show $\mathbf{x}^N$ is a predecessor of $\mathbf{y}_1^{M_1}$ (i.e. Definition S2.5 is satisfied)

Suppose a network state $\mathbf{z}$ contains $\mathbf{x}^N$.

Then, since $\mathbf{x}^N$ is a predecessor of $\mathbf{y}_2^{M_2}$, Definition S2.5 implies $\mathbf{f}(\mathbf{z})$ contains $\mathbf{y}_2^{M_2}$. Therefore, $\mathbf{f}(\mathbf{z})$ also contains $\mathbf{y}_1^{M_1}$ (since $\mathbf{y}_2^{M_2}$ contains $\mathbf{y}_1^{M_1}$).

Therefore, $\mathbf{x}^N$ is a predecessor of $\mathbf{y}_1^{M_1}$ (as required)

□

Lemma. S2.10

Suppose, $\mathbf{x}^N$ is a k-predecessor of $\mathbf{y}^M$ in $\mathbf{z}_a \in A$.

If a partial state $\mathbf{z}^P$ contains $\mathbf{x}^N$ and is contained in $\mathbf{z}_a$, then $\mathbf{z}^P$ is a k-predecessor of $\mathbf{y}^M$ in $\mathbf{z}_a$ $\in A$.

PROOF:

Since $\mathbf{x}^N$ is a k-predecessor of $\mathbf{y}^M$ in $\mathbf{z}_a \in A = \{\mathbf{z}_0, ..., \mathbf{z}_{p-1}\}$, Definition S2.9 implies that

1. $\mathbf{x}^N$ and $\mathbf{y}^M$ are both contained in the attractor state $\mathbf{z}_a$
2. There exists a sequence of partial states $\mathbf{z}_0^{P_0}$, $\mathbf{z}_1^{P_1}$, ..., $\mathbf{z}_k^{P_k}$ for which the following are true
   - **(a)** $k = cp$, where c is a positive integer.
   - **(b)** $P_0 = N$ and $\mathbf{z}_0^{P_0} = \mathbf{x}^N$
   - **(c)** $P_k = M$ and $\mathbf{z}_k^{P_k} = \mathbf{y}^M$
   - **(d)** For $i = 0, ..., k-1$, $\mathbf{z}_i^{P_i}$ is a predecessor of $\mathbf{z}_{i+1}^{P_{i+1}}$
   - **(e)** For $i = 0, ..., k$, $\mathbf{z}_i^{P_i}$ is contained in the attractor state $\mathbf{z}_b \in A$ (where $b = a + i \pmod p$)

We need to show that the same properties hold when $\mathbf{x}^N$ is replaced by $\mathbf{z}^P$.

Property 1 is still satisfied since $\mathbf{z}^P$ is contained in $\mathbf{z}_a$.

We now show Property 2 holds for the sequence of partial states $\mathbf{w}_0^{L_0}$, $\mathbf{w}_1^{L_1}$, ..., $\mathbf{w}_k^{L_k}$ where

- $L_0 = P$ and $\mathbf{w}_0^{L_0} = \mathbf{z}^P$
- $L_i = P_i$ and $\mathbf{w}_i^{L_i} = \mathbf{z}^{P_i}$ (for $i = 1, ..., k$)

(i.e. the same partial states as above except that $\mathbf{z}_0^{P_0} = \mathbf{x}^N$ is replaced by $\mathbf{z}^P$)

Obviously, properties 2(a), (b) and (c) still hold. Also 2(d) and 2(e) still hold for $i \geq 1$ (since $\mathbf{w}_1^{L_1}$, ..., $\mathbf{w}_k^{L_k}$ are equal to $\mathbf{z}_1^{P_1}$, ..., $\mathbf{z}_k^{P_k}$ (resp)). Therefore, it just remains to show 2(d) and 2(e) still hold for $i = 0$.

Case: 2(d) and i = 0.

We need to show $\mathbf{z}^P$ is a predecessor of $\mathbf{w}_1^{L_1} = \mathbf{z}_1^{P_1}$ (i.e. Definition S2.5 is satisfied)

If a network state $\mathbf{z}$ contains $\mathbf{z}^P$, then $\mathbf{z}$ also contains $\mathbf{x}^N$ (since $\mathbf{z}^P$ contains $\mathbf{x}^N$). Therefore, since $\mathbf{x}^N$ is a predecessor of $\mathbf{z}_1^{P_1}$ (by the old property 2(d)), Definition S2.5 implies $\mathbf{f}(\mathbf{z})$ contains $\mathbf{z}_1^{P_1} = \mathbf{w}_1^{L_1}$. Therefore, $\mathbf{z}^P$ is a predecessor of $\mathbf{w}_1^{L_1}$ (as required)

Case: 2(e) and i = 0.

Since $\mathbf{z}^P$ is contained in $\mathbf{z}_a$, $\mathbf{w}_0^{L_0} = \mathbf{z}^P$ is contained in the attractor state $\mathbf{z}_b \in A$ (where $b = a + 0 \pmod p = a$).

□

Theorem. S2.12

At the end of Procedure S2.11

1. The set $\mathbf{U}$ is non-empty
2. If a partial state $\mathbf{x}^N$ is contained in $\mathbf{U}$ at the end of the procedure, then
   (a) $\mathbf{x}^N$ is a k-predecessor of $\mathbf{y}^M$ in $\mathbf{z}_a \in A$
   (b) $\mathbf{x}^N$ contains no other partial states in $\mathbf{U}$

PROOF:

Part 1 : U is non-empty

We show that $\mathbf{W}_m$ is non-empty at the end of the procedure, for all $m \in \{0, ..., p\}$.

Then, when $t = p \quad (t \pmod p = 0)$, partial states in $\mathbf{W}_t$ will be added to $\mathbf{U}$ in Step 6 (since $\mathbf{U}$ is empty at the start of the procedure and the step $t = p$ is the first time Step 6 is visited). Since $\mathbf{U}$ is never emptied when Step 6 is visited in the future, $\mathbf{U}$ will then be non-empty at the end of the procedure.

Below, we prove $\mathbf{W}_m$ is non-empty at the end of the procedure, for all $m \in \{0, ..., p\}$. We do this by induction on m.

case $m = 0$:

$\mathbf{W}_0$ is non-empty at the start of the procedure. Then, for the rest of the procedure, partial states are only removed from $\mathbf{W}_i$ for $i \geq 1$. Therefore, $\mathbf{W}_0$ is non-empty at the end of the procedure

case $m > 0 : m \leq p$

In each loop of the procedure t increases by 1 (in step 2).

Since $\mathbf{U}$ is empty at the start of the procedure and Step 6 is not visited until time $t = p$, partial states are not removed from $\mathbf{W}_m$ in Step 6 when $(m \leq p)$. Therefore, partial states are only removed from $\mathbf{W}_m$ $(m \leq p)$ in steps 3 or 4 (when $t = m$).

Therefore, if $\mathbf{W}_m$ is non-empty at the end of step 4 (when $t \geq m$), $\mathbf{W}_m$ non-empty at the end of the procedure.

Suppose, $\mathbf{W}_0$, ..., $\mathbf{W}_{m-1}$ are non-empty at the end of Step 4, when $t = m - 1$. Then, because of Steps 2 and 4, every $\mathbf{z}^P \in \mathbf{W}_{m-1}$ satisfies

A: $\mathbf{z}^P$ is contained in the attractor state $\mathbf{z}_{t'}$ (where $t' = a - m + 1 \pmod p$)

Now, for the attractor $A = \{\mathbf{z}_0, ..., \mathbf{z}_{p-1}\}$, $\mathbf{f}(\mathbf{z}_i) = \mathbf{z}_j$ for all $i = 0, ..., p - 1$ and $j = i + 1 \pmod p$ (by the definition of an attractor in these models). Therefore,

B: $\mathbf{f}(\mathbf{z}_{m'}) = \mathbf{z}_{t'}$ (where $m' = a - m \pmod p$)

C: $\mathbf{f}(\mathbf{z}_{m'})$ contains $\mathbf{z}^P$ (by **A** and **B**)

D: $\mathbf{z}_{m'}$ is a predecessor of $\mathbf{z}^P$ (by **C** and Definition S2.5)

After returning to Step 1 in the next loop of the procedure, Procedure S2.8 is applied to every partial state $\mathbf{z}^P \in \mathbf{W}_{m-1}$. This identifies partial states $\mathbf{x}_1^{N_1}$, ..., $\mathbf{x}_s^{N_s}$ where

1. Each $\mathbf{x}_i^{N_i}$ is a predecessor of some $\mathbf{z}^P \in \mathbf{W}_{m-1}$
2. If an attractor state $\mathbf{z}$ is a predecessor of $\mathbf{z}^P$, then $\mathbf{z}$ contains $\mathbf{x}_i^{N_i}$ for some $i \in \{1, ..., s\}$

$\mathbf{x}_1^{N_1}$, ..., $\mathbf{x}_s^{N_s}$ are then added to $\mathbf{W}_m$

Therefore (because of **D** and 2 above), the attractor state $\mathbf{z}_{m'}$ ($m' = a - m \pmod{p}$) contains $\mathbf{x}_i^{N_i}$ for some $i \in \{1, ..., s\}$. Letting $\mathbf{x}_i^{N_i}$ be the smallest such partial state (i.e. $\mathbf{x}_i^{N_i}$ does not contain any other $\mathbf{x}_j^{N_j}$; $j \in \{1, ..., s\}, j \neq i$)

E: $\mathbf{x}_i^{N_i}$ is contained in $\mathbf{z}_{m'}$

F: $\mathbf{x}_i^{N_i}$ does not contain a different $\mathbf{x}_j^{N_j} \in \mathbf{W}_m$

Moving onto Step 2, $t = m$ and $t' = m' = a - m \pmod{p}$

Moving onto Step 3, $\mathbf{x}_i^{N_i}$ is **not** removed from $\mathbf{W}_m$ (because of **F**)

Moving onto Step 4, $\mathbf{x}_i^{N_i}$ is **not** removed from $\mathbf{W}_m$ (because of **E**)

Therefore, $\mathbf{W}_m$ is not empty at the end of Step 4 and the end of the Procedure.

Therefore, $\mathbf{W}_m$ is non-empty for all $m \in \{0, ..., p\}$ (as required)

Part 2a:

Given any $\mathbf{x}^N \in \mathbf{U}$, we need to show that $\mathbf{x}^N$ is a k-predecessor of $\mathbf{y}^M$ in $\mathbf{z}_a \in A$. To do this, we show that the properties of Definition S2.9 is satisfied.

Each loop in the procedure corresponds to a single time step. Following the loop backwards from the time $\mathbf{x}^N$ is put in $\mathbf{U}$, we get a sequence of partial states $\mathbf{z}_0^{P_0}$, ..., $\mathbf{z}_i^{P_i}$, ..., $\mathbf{z}_k^{P_k}$ (from $\mathbf{W}_k$, ..., $\mathbf{W}_{k-i}$, ..., $\mathbf{W}_0$ respectively), where

(a) $k = cp$ for some positive integer c (where c is the number of times Step 6 has been visited when $\mathbf{x}^N$ was added to $\mathbf{U}$)

(b) $P_0 = N$ and $\mathbf{z}_0^{P_0} = \mathbf{x}^N$

(c) $P_k = M$ and $\mathbf{z}_k^{P_k} = \mathbf{y}^M$

(d) For $i = 0, ..., k-1$, $\mathbf{z}_i^{P_i}$ is a predecessor of $\mathbf{z}_{i+1}^{P_{i+1}}$ (because of Step 1 of the procedure)

Therefore, to show Property 2 of Definition S2.9 is satisfied, it just remains to show

(e) For $i = 0, ..., k,$ $\mathbf{z}_i^{P_i}$ is contained in the attractor state $\mathbf{z}_b \in A$ (where $b = a + i \pmod{p}$)

A partial state $\mathbf{z}_i^{P_i}$ only remains in $\mathbf{W}_{k-i}$ after Step 4, if it is contained in the attractor state $\mathbf{z}_{t'}$ (where $t' = a - (k - i) \pmod{p}$). Therefore, since $k \pmod{p} = 0$, (e) is satisfied by letting $b = t' = a + i \pmod{p}$.

Property 1 of Definition S2.9 is satisfied, because

- (b) and (e) imply that $\mathbf{x}^N$ is contained in $\mathbf{z}_a$
$(i = 0 \implies b = a \pmod{p} = a)$

- (a), (c) and (e) imply that $\mathbf{y}^M$ is contained in $\mathbf{z}_a$
$(i = k$ and $k \pmod{p} = 0 \implies b = a \pmod{p} = a)$

Part 2b:

We use proof by contradiction to show 2b is satisfied. Suppose $\mathbf{x}^N$ contains a partial state $\mathbf{u}^L$ in $\mathbf{U}$. Then, $\mathbf{x}^N$ could never of ended up in $\mathbf{U}$ because

By Step 3: $\mathbf{x}^N$ and $\mathbf{u}^L$ can't both occur in some W_t
(and so couldn't be put in $\mathbf{U}$ at the same time)

By Step 6: $\mathbf{x}^N$ could not be put in $\mathbf{U}$, if $\mathbf{u}^L$ was already in $\mathbf{U}$

By Step 6: $\mathbf{x}^N$ would be removed from $\mathbf{U}$, when $\mathbf{u}^L$ was added.

□

Lemma. S2.16

Consider two collections of subsystems $\mathbb{S}_a$ and $\mathbb{S}_b$, and suppose

(a) $\mathbb{S}_a$ triggers an individual subsystem S_y in an attractor $A = \{\mathbf{z}_0, ..., \mathbf{z}_{p-1}\}$

(b) $\mathbb{S}_b \supset \mathbb{S}_a$

(c) Every subsystem $S \in \mathbb{S}_b$ occurs in A

Then, $\mathbb{S}_b$ triggers S_y in A

PROOF:

Let,

A: $\mathbf{x}_0^{M_a}, ..., \mathbf{x}_{p-1}^{M_a}$ be the instance of $\mathbb{S}_a$ in $A = \{\mathbf{z}_0, ..., \mathbf{z}_{p-1}\}$

B: $\mathbf{y}_0^{M_b}, ..., \mathbf{y}_{p-1}^{M_b}$ be the instance of $\mathbb{S}_b$ in $A = \{\mathbf{z}_0, ..., \mathbf{z}_{p-1}\}$

C: $\mathbf{z}_0^{M_y}, ..., \mathbf{z}_{p-1}^{M_y}$ be the instance of S_y in $A = \{\mathbf{z}_0, ..., \mathbf{z}_{p-1}\}$

Then, it must be the case that

D: $M_b \supseteq M_a$ (from property 1 of Definition S2.13 and the fact that $\mathbb{S}_b \supset \mathbb{S}_a$)

E: For $i = 0, ..., p-1$, $\mathbf{y}_i^{M_b}$ contains $\mathbf{x}_i^{M_a}$ (from **D** and property 2 of Definition S2.13)

F: For $i = 0, ..., p-1$, $\mathbf{y}_i^{M_b}$ is contained in the attractor state $\mathbf{z}_i$ (from property 2 of Definition S2.13)

Additionally, since $\mathbb{S}_a$ triggers S_y in an attractor A, Definition S2.14 implies

G: For every $i \in \{0, ..., p-1\}$, $\mathbf{x}_i^{M_a}$ is a k_i-predecessor of $\mathbf{z}_i^{M_y}$ in $\mathbf{z}_i \in A$ (for some k_i)

Therefore, because of **E**, **F**, **G** and Lemma S2.10

H: For every $i \in \{0, ..., p-1\}$, $\mathbf{y}_i^{M_b}$ is a k_i-predecessor of $\mathbf{z}_i^{M_y}$ in $\mathbf{z}_i \in A$

From (a) and (c), we know that every S_y occurs in A and every $S \in \mathbb{S}_b$ occurs in A.

Therefore, in order to show $\mathbb{S}_b$ triggers S_y in A we just need to show the last property of Definition S2.14 holds. This is true because of **B**, **C** and **H** (above)

□

Theorem. S2.20

At the end of Procedure S2.19

1. Whenever $A_i \in \mathbb{C}_y$,
   - **(a)** $\mathbf{R}_i$ is non-empty
   - **(b)** Every subsystem collection $\mathbb{S} \in \mathbf{R}_i$ triggers S_y in A_i
2. $\mathbf{R} = \{\mathbb{S}_1, ..., \mathbb{S}_g\}$ is a regulation set of S_y

PROOF:

Part 1a

When $A_i = \{\mathbf{z}_0, ..., \mathbf{z}_{p-1}\}$ is being analysed, $\mathbf{R}_i$ could only be empty at the end of the loop (and hence the end of the procedure) if either

A: Step 2
There is no subsystem T that occurs in A_i

B: Step 4
$\mathbf{U}_j$ is empty for some $j \in \{0, ..., p-1\}$

C: Step 5
There is no subsystem T_a (involving a node set M_a) that occurs in A_i and satisfies $M_a \cap N \neq \varnothing$ (for a set N given in Step 5)

D: Step 6
All subsystem collections are removed from $\mathbf{R}_i$ in Step 6

Now, by Theorem S1.20 in *Supporting Text 1* (Section S1.3), given an attractor A and node n_i, there exists a subsystem $S = \{\mathbf{x}_0^N, \mathbf{x}_1^N, ..., \mathbf{x}_{q-1}^N\}$ for which

(a) $n_i \in N$

(b) S occurs in A

Therefore, **A** and **C** cannot be true.

B cannot be true, because Procedure S2.11 always finds k-predecessors for $\mathbf{U}_j$ (by Theorem S2.12)

D cannot be true, otherwise they would be collections of subsystems $\mathbb{S}_a$, $\mathbb{S}_b \in \mathbf{R}_i$ such that $\mathbb{S}_a \subset \mathbb{S}_b \subset \mathbb{S}_a$ (which is impossible).

Therefore, each $\mathbf{R}_i$ is non-empty

Part 1b

We need to show that any collection of subsystems $\mathbb{S}_x = \{T_{x_1}, ..., T_{x_f}\} \in \mathbf{R}_i$ triggers S_y in $A_i = \{\mathbf{z}_0, ...,\mathbf{z}_{p-1}\}$ (i.e. Definition S2.14 is satisfied)

First note that subsystem collections $T_{x_1}, ..., T_{x_f}, S_y$ all occur in A_i; since only subsystems that occur in A_i are considered in the corresponding loop of the procedure.

Then, letting $M_1, ..., M_f, M_y$ be the node sets involved in $T_{x_1}, ..., T_{x_f}, S_y$ (resp) we get

(a) $\mathbf{z}_0^{M_x}, ...,\mathbf{z}_{p-1}^{M_x}$ is the instance of $\mathbb{S}_x$ in A_i (where $M_x = M_1 \cup ... \cup M_f$, see Definition S2.13)

(b) $\mathbf{y}_0^{M_y}, ...,\mathbf{y}_{p-1}^{M_y}$ is the instance of S_y in A_i (from Step 3 of procedure)

Therefore, to show that Definition S2.14 is satisfied (i.e. $\mathbb{S}_x$ triggers S_y in A_i), we need to show that

(c) For $j = 0, ..., p-1$, $\quad \mathbf{z}_j^{M_x}$ is a k_j-predecessor of $\mathbf{y}_j^{M_y}$ in $\mathbf{z}_j \in A_i$ (for some k_j)

Now following from Step 4 and 5 in the procedure, there exists a set of partial states $\mathbf{x}_0^{N_0}$, ..., $\mathbf{x}_{p-1}^{N_{p-1}}$ such that for $j = 0, ..., p-1$

E: $\mathbf{x}_j^{N_j} \in \mathbf{U}_j$

F: $\mathbf{x}_j^{N_j}$ is a k_j predecessor of $\mathbf{y}_j^{M_y}$ in $\mathbf{z}_j \in A_i$ (for some k_j). This follows from **E** and Step 4.

Moreover, because of **F**, (a), (b), Definition S2.13 and Definition S2.14

G: $\mathbf{x}_j^{N_j}$, $\mathbf{y}_j^{M_y}$ and $\mathbf{z}_j^{M_x}$ are all contained in the same attractor state $\mathbf{z}_j \in A_i$

Now, from Theorem S1.20 in *Supporting Text 1* (Section S1.3), given a node $n_i \in N$, it is possible to find a subsystem T_{x_a} that occurs in A_i and involves a node set M_a satisfying $n_i \in M_a$.

Therefore, for every node $n_i \in N$, a subsystem T_{x_a} would be added to $\mathbb{S}_x$ in Step 5, thus implying that

H: $M_x = M_1 \cup ... \cup M_f \supseteq N$

Moreover, from Step 5a,

I: $N \supseteq N_j$ (for $j = 0, ..., p-1$)

Therefore, **G**, **H** and **I** imply

J: For $j = 0, ..., p-1$, $\mathbf{z}_j^{M_x}$ contains $\mathbf{x}_j^{N_j}$

Therefore, because of Lemma S2.10, **F**, **G** and **J** imply

- For $j = 0, ..., p-1$, $\mathbf{z}_j^{M_x}$ is a k_j-predecessor of $\mathbf{y}_j^{M_y}$ in $\mathbf{z}_j \in A_i$ (for some k_j)

This is the condition required to show $\mathbb{S}_x$ triggers S_y in A_i

Part 2

To show $\mathbf{R} = \{\mathbb{S}_1, ..., \mathbb{S}_g\}$ is a regulation set of S_y, we need to show that the 2 properties of Definition S2.15 hold.

First, property 1.

For any $j \in 1, ..., g$, $\mathbb{S}_j \in \mathbf{R}_i$ for some i (otherwise $\mathbb{S}_j$ would never have been added to $\mathbf{R}$ in Step 7 of the procedure). Therefore, $\mathbb{S}_j$ triggers S_y in A_i (by part 1b of this Theorem)

Now, property 2.

Suppose S_y occurs in an attractor A_i. Then, since $\mathbf{R}_i$ in non-empty (by part 1a of this Theorem), there exists a collection of subsystems $\mathbb{S}_j \in \mathbf{R}_i$ that triggers S_y in A_i (by part 1b of this Theorem).

Therefore, since $\mathbf{R}$ is just a combination of the $\mathbf{R}_i$'s (see Step 7 of procedure), there exists a collection of subsystems $\mathbb{S}_j \in \mathbf{R}$ that triggers S_y in A_i

□

References

Irons DJ (2006) *Physica D* **217** 7–21
