# Supplementary material for: Identifying dynamical modules from genetic regulatory systems: applications to the segment polarity network
Source: BMC Bioinformatics. 2007 Oct 25;8:413. doi: 10.1186/1471-2105-8-413 (PMC2233651; doi:10.1186/1471-2105-8-413)
Supplement: Additional file 3 — Extra examples associated with the method of identifying subsystems. This is given as a pdf file. [file 1471-2105-8-413-S3.pdf]

Supporting Text 3:
Supplementary Examples

In order to demonstrate the methods in the main text, we run through simple Boolean network models to look at

1. Partial state sequences that *occur* within attractors (Definition 3)
2. Identifying subsystems (Definition 4,5 and 6)
3. Discovering how subsystems are regulated (Definition 7)

The theory behind Definitions 3 - 7 is discussed using the examples in

1. Section S3.1 (Definition 3)
2. Section S3.2 (Definitions 4-6)
3. Section S3.3 (Definitions 6-7)

Associated algorithms are described in *Supporting Text 1*.

S3.1 Example 1: Definition 3

Here, we explain the main features of this definition, by use of the simple example shown in Fig.S3.1. For $N = \{n_1, n_2\}$, the partial state sequence

$$P = \begin{cases} \mathbf{x}_0^N = \{s_1 = 0, s_2 = 0\}, \\ \mathbf{x}_1^N = \{s_1 = 1, s_2 = 0\}, \\ \mathbf{x}_2^N = \{s_1 = 1, s_2 = 1\}, \\ \mathbf{x}_3^N = \{s_1 = 0, s_2 = 1\} \end{cases} =$$

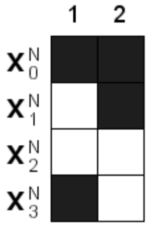

occurs within the two attractors in Fig.S3.1 (B and C) since the partial states cycle with the attractors. The 3 properties of Definition 3 are satisfied using integers b_0, ..., b_{p-1} shown in the tables below

B

k	0	1	2	3	4	5	6	7
b_k	0	1	1	2	3	3	3	3

C

k	0	1	2	3	4	5	6	7	8	9
b_k	0	1	1	1	1	2	3	3	3	3

Property 1 is satisfied since $\mathbf{z}_k$ contains $\mathbf{x}_{b_k}^N$, for $k = 0, ..., p-1$. Property 2 and 3 are satisfied because b_k (Position in P) cycles through 0, ..., $q-1$ as k (Position in A) cycles through 0, ...,$p-1$; whilst the time between each change in P is ignored. Another way of looking at this definition, is that there is a function $f : A \rightarrow P$ that maps the states in A (modulo p) onto the states in P (modulo q).

Properties 2 and 3 imply that only the logical transition of partial states within the attractor is important, not the exact length of time each partial state $\mathbf{x}_i^N \in P$ remains. In the example, $\mathbf{x}_1^N = \{s_1 = 1, s_2 = 0\}$ takes an extra two time steps in Fig.S3.1C once the inhibitor n_3 is active. Since the logical relationship between n_1 and n_2 is the same in both attractor cycles (i.e. n_1 and n_2 form a negative feedback loop, which creates oscillatory behaviour), it may be advantageous to ignore these time lags. Taking account of such time lags could be especially important in genetic regulatory systems, where the same process may take different lengths of time under different conditions. In the example of cell cycle regulation, some mutations can alter the time taken for a round of cell division, without necessarily altering the relationships that exist between other proteins (Chen *et al*, 2004).

Property 3 ensures that P is the smallest possible set of partial states that cycles with A. This leaves a partial state sequence that just describes the ′order′ in which the node states change in A (for nodes in N).

Firstly, as is the case in the above example, if a partial state $\mathbf{x}_i^N \in P$ occurs for ′c′ consecutive time steps in A, $\mathbf{x}_i^N$ is only written down once. However, this does not prevent $\mathbf{x}_i^N$ occurring later on in P.

Secondly, if a partial state sequence cycles multiple times within an attractor cycle, only a single copy is kept. For example, in the Boolean network model in Fig.S3.2, the partial state sequence in Fig.S3.2C occurs in the attractor in Fig.S3.2D and cycles 3 times within it. The 3 properties of Definition 3 are satisfied using integers b_0, ..., b_{p-1} shown in the table below

k	0	1	2	3	4	5
b_k	1	0	1	0	1	0

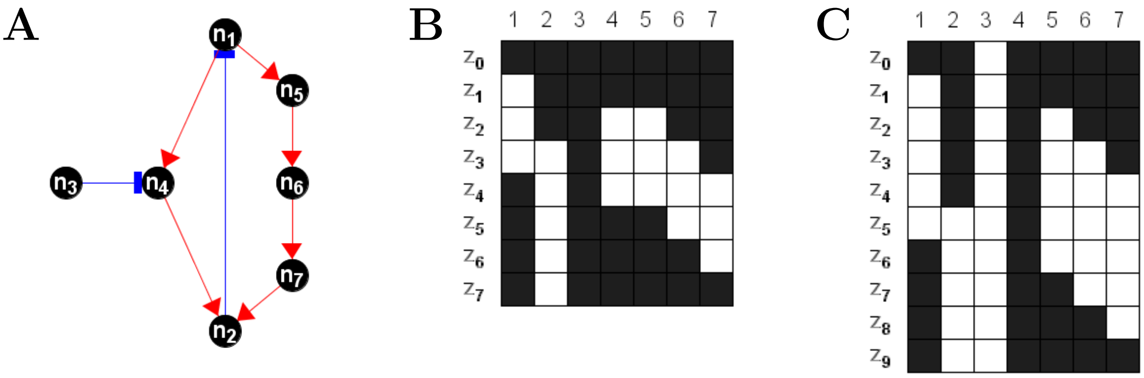

D

Node	Boolean function
n_1	$s_1(t+1) = 1$ IF $s_2(t) = 0$<br>$s_1(t+1) = 0$ otherwise
n_2	$s_2(t+1) = 1$ IF $s_4(t) = 1$ OR $s_7(t) = 1$<br>$s_2(t+1) = 0$ otherwise
n_3	$s_3(t+1) = 1$ IF $s_3(t) = 1$<br>$s_3(t+1) = 0$ otherwise
n_4	$s_4(t+1) = 1$ IF $s_1(t) = 1$ AND $s_3(t) = 0$<br>$s_4(t+1) = 0$ otherwise

Node	Boolean function
n_5	$s_5(t+1) = 1$ IF $s_1(t) = 1$<br>$s_5(t+1) = 0$ otherwise
n_6	$s_6(t+1) = 1$ IF $s_5(t) = 1$<br>$s_6(t+1) = 0$ otherwise
n_7	$s_7(t+1) = 1$ IF $s_6(t) = 1$<br>$s_7(t+1) = 0$ otherwise

Figure S3.1: (A) Example of a Boolean network model with Boolean functions in D. B and C are attractors for the Boolean network model. Here white / black corresponds to the node having state 1 / 0 (resp). (B) Attractor involving 8 network states. (C) Attractor involving 10 network states, where one of the paths from n_1 to n_2 is blocked by n_3.

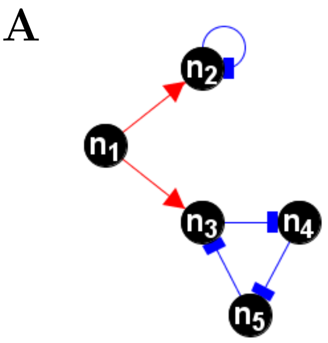

B

Node	Boolean function
n_1	$s_1(t+1) = 1$ IF $s_1(t) = 1$<br>$s_1(t+1) = 0$ otherwise
n_2	$s_2(t+1) = 1$ IF $s_1(t) = 1$ AND $s_2(t) = 0$<br>$s_2(t+1) = 0$ otherwise
n_3	$s_3(t+1) = 1$ IF $s_1(t) = 1$ AND $s_5(t) = 0$<br>$s_3(t+1) = 0$ otherwise
n_4	$s_4(t+1) = 1$ IF $s_3(t) = 0$<br>$s_4(t+1) = 0$ otherwise
n_5	$s_5(t+1) = 1$ IF $s_4(t) = 0$<br>$s_5(t+1) = 0$ otherwise

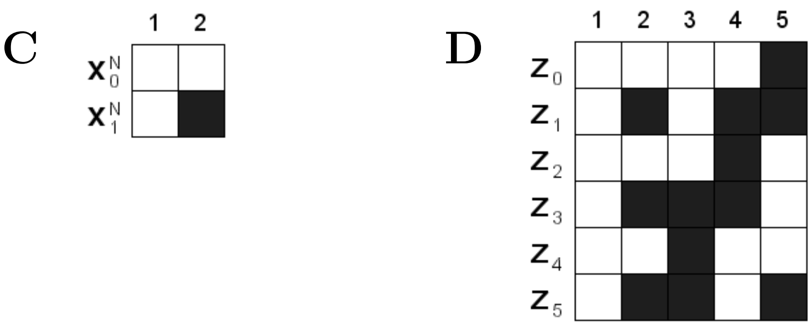

Figure S3.2: (A) Example of a Boolean network model with Boolean functions in B. C is a partial state sequence that occurs in the attractor in D. Here white / black corresponds to the node having state 1 / 0 (resp)

S3.2 Example 2: Subsystems and their regulation

This example corresponds to the simple Boolean network model in Fig.S3.3.

S3.2.1 Subsystems

In order to demonstrate the method of finding subsystems, we use this example to describe and justify the main features of Definitions 4, 5 and 6 (one at a time).

The model in Fig.S3.3 has,

(a) 5 attractors A_1, ..., A_5.

(b) : Definition 4
11 *intersection sequences* P_1, ..., P_{11} that *intersect at* sets of attractors $\mathbb{C}_1$, ..., $\mathbb{C}_{11}$ respectively (see Fig.S3.3 and Table S3.1A)

(c) : Definition 5
9 *partition sequences* P_1, ..., P_8, P_{12} (see Fig.S3.3 and Table S3.1B)

(d) : Definition 6
5 *subsystems* S_1, ..., S_5 (see Fig.S3.4)

As explained in the main text, the method of identifying subsystems is a 2 stage process that breaks up the system's attractors, to leave partial state sequences that are optimally distinguishable from one another. The first stage involves identifying *partition sequences* (satisfying Definition 5), whilst the second stage uses these partition sequences to identify *subsystems* (satisfying Definition 6)

Stage 1 : Identifying Partition Sequences

This stage works by first identifying all intersection sequences that satisfy Definition 4. These are then used as the basis for identifying partition sequences (satisfying Definition 5). Below, we explain Definitions 4 and 5. An algorithm for identifying all partition sequences is given in *Supporting Text 1* (Section S1.2).

Intersection sequences provide a hierarchical breakdown of the set of attractors. Any partial state sequences P that satisfies Definition 4 will be conserved across a set of attractors $\mathbb{C}$ (by properties 1 and 2). Moreover, property 3 ensures that the full set of intersection sequences P_1, ..., P_r ($r = 11$ in this case) give a strict hierarchical breakdown of the global dynamics (the attractors A_1, ..., A_5 in this case). To see this hierarchy, consider the intersection sequences in Fig.S3.5A and Fig.S3.3. If a link joins an intersection sequence P_x (top) to another P_y (bottom) then

1. $N_x \subset N_y$ (where N_i is the node set for P_i)
2. $C_x \supset C_y$ (where P_i intersects at C_i)
3. P_x occurs in P_y

This hierarchy exists because of property 3 of Definition 4. Given an intersection sequence P (for a node set N), the addition of any extra node $n_k \notin N$ implies that is no longer possible to find a single partial state sequence P' (for the larger node set $N \cup \{n_k\}$) that is conserved across the same set of attractors ($\mathbb{C}$ say). Instead, either

1. Adding n_k to P leads to new partial state sequences Q_1, ..., Q_j that occur within lower order intersection sequences P'_1, ..., P'_j. Each one of these lower order intersection sequences will be distinct and conserved across a smaller set of attractors $\mathbb{C}'_1$, ..., $\mathbb{C}'_j$ (each one contained in $\mathbb{C}$)
2. P is already an attractor and occurs at the bottom of the hierarchy.

For example, P_9 involves nodes n_1 and n_3 and occurs in 4 attractors A_2, A_3, A_4 and A_5. However, after taking any of the nodes n_2, n_4 or n_5 into consideration (alongside P_9), it is no longer possible to find a partial state sequence that occurs in A_2, A_3, A_4 and A_5. Instead, we get the following

Adding n_2
Adding n_2 to P_9 leads to new partial state sequences Q_1, Q_2, Q_3, Q_4 that occur within lower order intersection sequences P_2, P_3, P_4 and P_5.

Adding n_4
Adding n_4 to P_9 leads to new partial state sequences Q_5 and Q_6 that occur within lower order intersection sequences P_6 and P_7.

Adding n_5
Adding n_5 to P_9 leads to new partial state sequences Q_7 and Q_8 that occur within lower order intersection sequences P_6 and P_7.

Although Definition 4 provides us with a hierarchical breakdown of the attractors, some of the resulting sequences may not be fundamental to the make up of an attractor. Definition 5 provides a stronger way of partitioning up the dynamics in a hierarchical manner.

In this example, P_9, P_{10} and P_{11} all occur in exactly the same set of attractors and branch off from a common *core component* ($P_{12} = \{s_1 = 1\}$). Moreover, P_9, P_{10} and P_{11} combine together in two non-independent ways to form the intersection sequences P_6 and P_7. Therefore, P_9, P_{10} and P_{11} aren't fundamental to the make up of an attractor, rather intermediaries between the common core component P_{12} and the intersection sequences P_6 and P_7.

To filter out such intermediaries, Definition 5 uses the information about intersection sequences to look for partial state sequences P that satisfy one the following 3 stronger properties (for some set of attractors $\mathbb{C}$)

A : P is Core to $\mathbb{C}$
The following 3 properties hold for P

1. P occurs in an intersection sequence P', which intersects at $\mathbb{C}$ (P can equal P').
2. If an intersection sequence Q (for a node set M) intersects at $\mathbb{D}$ (where $\mathbb{D} \cap \mathbb{C} \neq \varnothing$), then there exists an intersection sequence Q' (for a node set $M' \supseteq M \cup N$) that occurs in every attractor $A \in \mathbb{D} \cap \mathbb{C}$
3. 1 and 2 are not true for any larger partial state sequence P'' (for a node set $N'' \supset N$)

B : P is Exclusive to $\mathbb{C}$

P is the only intersection sequence that intersects at $\mathbb{C}$.

C : P is Independently Oscillating

P intersects at $\mathbb{C}$ and cycles out of phase with another intersection sequence Q. i.e. $\exists\, Q$ that involves the node set M and intersects at $\mathbb{D}$, for which

1. $|\ \mathbb{C} \cap \mathbb{D}\ | \geq 2$
2. $N \cup M = V$ (the set of all nodes)

Returning to the simple example in Fig.S3.3, there are 9 partition sequences (see Table S3.1B). 8 of the 11 intersections sequences are also partition sequences because at least one of the following are true

A They are core to $\mathbb{C}_i$ (P_1, P_2, P_3, P_4 and P_5).

B They are exclusive to $\mathbb{C}_i$ (P_1, P_2, P_3, P_4, P_5, P_6 and P_7).

C They represent cyclic sub-dynamics that cycle *out of phase* to create more than 1 attractor (P_6, P_7 and P_8)

In the third case, P_6, P_7 and P_8 pair up as in two different ways. Pairs combine together in a quasi-independent way to create the attractors A_2, A_3, A_4 and A_5; as follows

(a) P_6 and P_8 combine in two different ways to create A_4 and A_5

(b) P_7 and P_8 combine in two different ways to create A_2 and A_3

The final partition sequence is P_{12}, which is core to the set of attractors $\mathbb{C}_{12} = \{A_2, A_3, A_4, A_5\}$. Although P_8, P_9, P_{10} and P_{11} occur in the same attractors they are not core to $\mathbb{C}_{12}$ because whenever one (P_x, say) occurs in an attractor, so does another (P_y, say) with a divergent node set. (i.e. $N_x \nsubseteq N_y$, $N_y \nsubseteq N_x$). Therefore, P_{12} acts as a central point from which these intersections sequences branch off.

As can be seen in Fig.S3.5C, a hierarchy is maintained between the partition sequences.

Stage 2 : Identifying Subsystems

Partition sequences can be viewed as being both stable and separable from the dynamics of the rest of the network. However, it is often the case that a partition sequence (P_x) is contained within another (P_y) and so they are not strictly distinct. Moreover, the attractors themselves are all classed as partition sequences. Therefore, subsystems are those components that are unique to a partition sequence. i.e. they are the units that separate and uniquely describe the key components of the attractors.

Returning to the simple Boolean network model in Fig.S3.3. This model has the 5 subsystems S_1, S_2, S_3, S_4 and S_5 (shown in Fig.S3.4) which are the unique components of the partition sequences P_1, P_6, P_7, P_8 and P_{12} (resp) from Fig.S3.3. As can be seen in Table S3.1C, not all partition sequences have a unique element. This is because they are a consequence of two or more smaller partition sequences.

Fig.S3.5D shows how the subsystems relate to the hierarchy between partition sequences. From this figure it is evident that subsystems can also link up in a hierarchical manner.

As expected, in such a basic example, the subsystems (identified purely from the dynamics) correspond well with the underlying network (Fig.S3.3A), with S_2, S_3 and S_4 representing the sub-dynamics of the two main network motifs. S_5 acts as a background condition that is both necessary for S_2, S_3 and S_4 and distinguishes $\mathbb{C} = \{A_2, A_3, A_4, A_5\}$ from $A_1 = S_1$, which is a distinct dynamic in its own right.

S3.2.2 Regulation of subsystems

Table S3.2 shows how each subsystem in this example is regulated. Each subsystem is dependent on itself, whilst the inter-subsystem dependencies are consistent with the underlying network.

Because of the simplicity of this example, we do not explain the details of Definition 7 and the method of identifying regulation sets here. Instead, we give a more detailed explanation with the next example (Example 3 in Section S3.3)

A

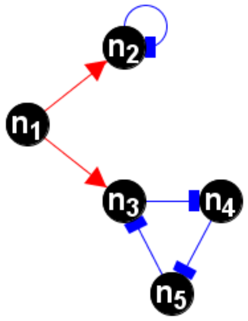

B

Node	Boolean function
n_1	$s_1(t+1) = 1$ IF $s_1(t) = 1$<br>$s_1(t+1) = 0$ otherwise
n_2	$s_2(t+1) = 1$ IF $s_1(t) = 1$ AND $s_2(t) = 0$<br>$s_2(t+1) = 0$ otherwise
n_3	$s_3(t+1) = 1$ IF $s_1(t) = 1$ AND $s_5(t) = 0$<br>$s_3(t+1) = 0$ otherwise
n_4	$s_4(t+1) = 1$ IF $s_3(t) = 0$<br>$s_4(t+1) = 0$ otherwise
n_5	$s_5(t+1) = 1$ IF $s_4(t) = 0$<br>$s_5(t+1) = 0$ otherwise

C

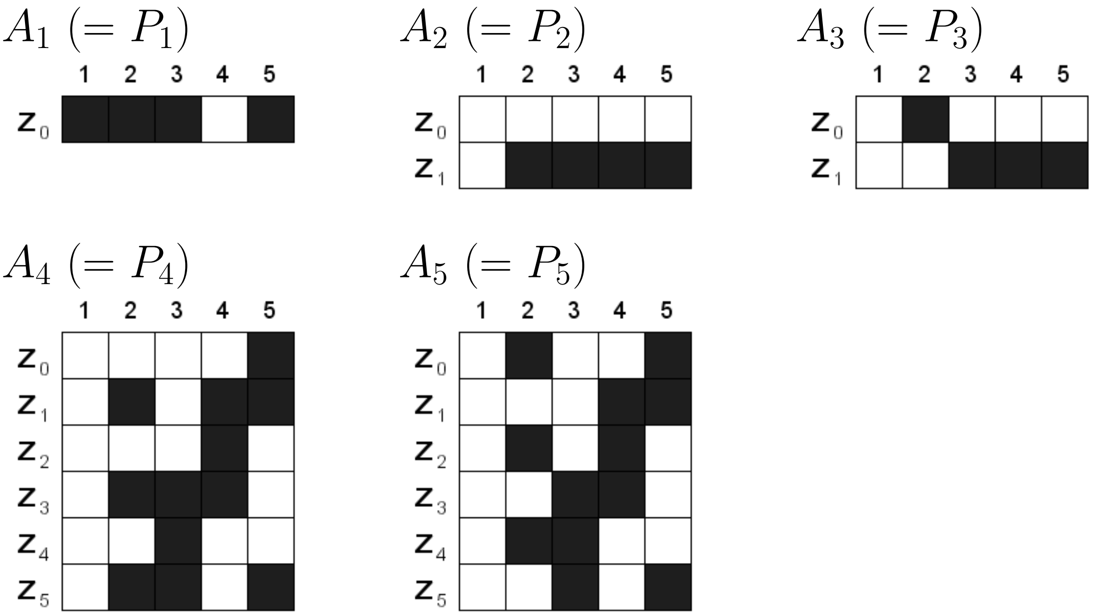

D

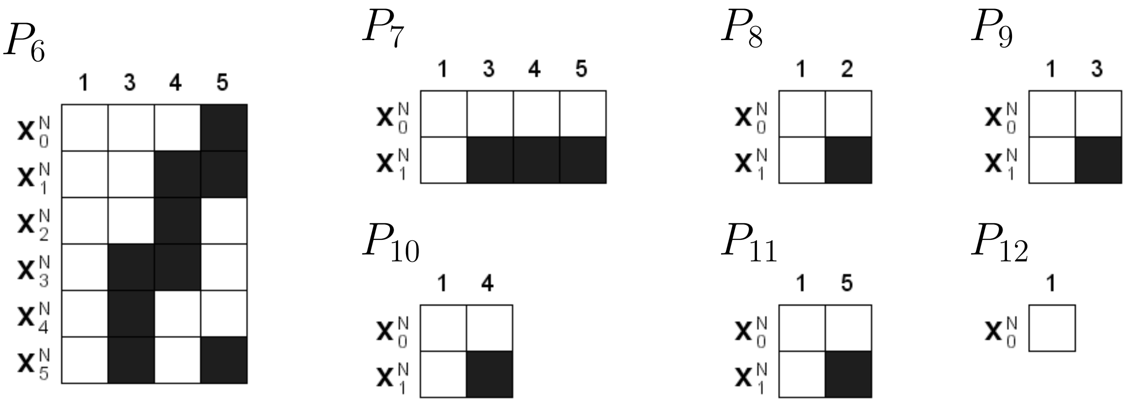

Figure S3.3: Example of a Boolean network model with the network shown in A, Boolean functions shown in B and attractors A_1 to A_5 shown in C (which are also partial state sequences). (D) Other partial state sequences of interest (discussed in text and Table S3.1). Here white / black corresponds to the node having state 1 / 0 (resp)

Table S3.1: Table summarising the properties of the partial state sequences in Fig.S3.3C and D. (A) Intersection sequences, along with the attractors they occur in. (B) Partition sequences along with which of properties A, B and C they satisfy in Definition 5. (C) The unique components of the partition sequence. These are the subsystems shown in Fig.S3.4

A

**Intersection sequences**	**Intersects at ...**
P_1	$\mathbb{C}_1 = \{A_1\}$
P_2	$\mathbb{C}_2 = \{A_2\}$
P_3	$\mathbb{C}_3 = \{A_3\}$
P_4	$\mathbb{C}_4 = \{A_4\}$
P_5	$\mathbb{C}_5 = \{A_5\}$
P_6	$\mathbb{C}_6 = \{A_4, A_5\}$
P_7	$\mathbb{C}_7 = \{A_2, A_3\}$
P_8	$\mathbb{C}_8 = \{A_2, A_3, A_4, A_5\}$
P_9	$\mathbb{C}_9 = \{A_2, A_3, A_4, A_5\}$
P_{10}	$\mathbb{C}_{10} = \{A_2, A_3, A_4, A_5\}$
P_{11}	$\mathbb{C}_{11} = \{A_2, A_3, A_4, A_5\}$

B

**Partition sequences**	**Core**	**Exclusive**	**Independently Oscillating with ...**
P_1	$\mathbb{C}_1 = \{A_1\}$	$\surd$	$\times$
P_2	$\mathbb{C}_2 = \{A_2\}$	$\surd$	$\times$
P_3	$\mathbb{C}_3 = \{A_3\}$	$\surd$	$\times$
P_4	$\mathbb{C}_4 = \{A_4\}$	$\surd$	$\times$
P_5	$\mathbb{C}_5 = \{A_5\}$	$\surd$	$\times$
P_6	$\times$	$\surd$	P_8
P_7	$\times$	$\surd$	P_8
P_8	$\times$	$\times$	P_6, P_7
P_{12}	$\mathbb{C}_{12} = \{A_2, A_3, A_4, A_5\}$	$\times$	$\times$

C

**Partition sequence**	**Unique Component**
P_1	S_1 (= P_1)
P_2	N/a
P_3	N/a
P_4	N/a
P_5	N/a
P_6	S_2
P_7	S_3
P_8	S_4
P_{12}	S_5

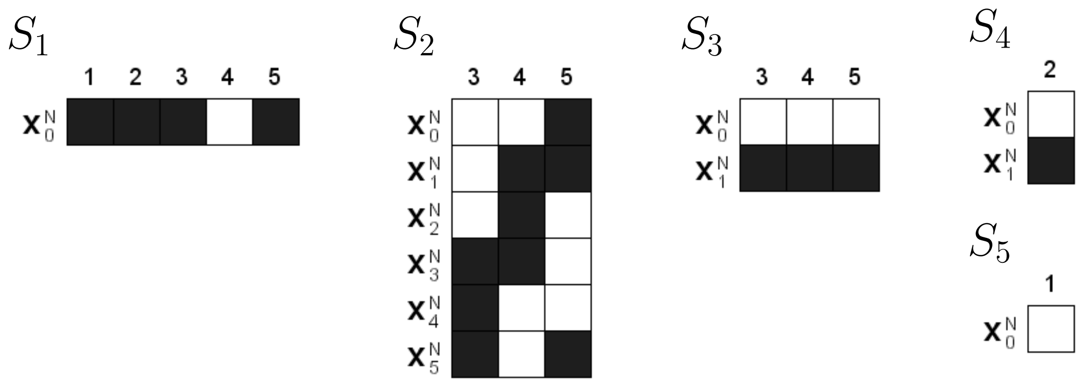

Figure S3.4: Subsystems for the simple Boolean network model in Fig.S3.3. These are the unique components of the partition sequences (Table S3.1B,C)

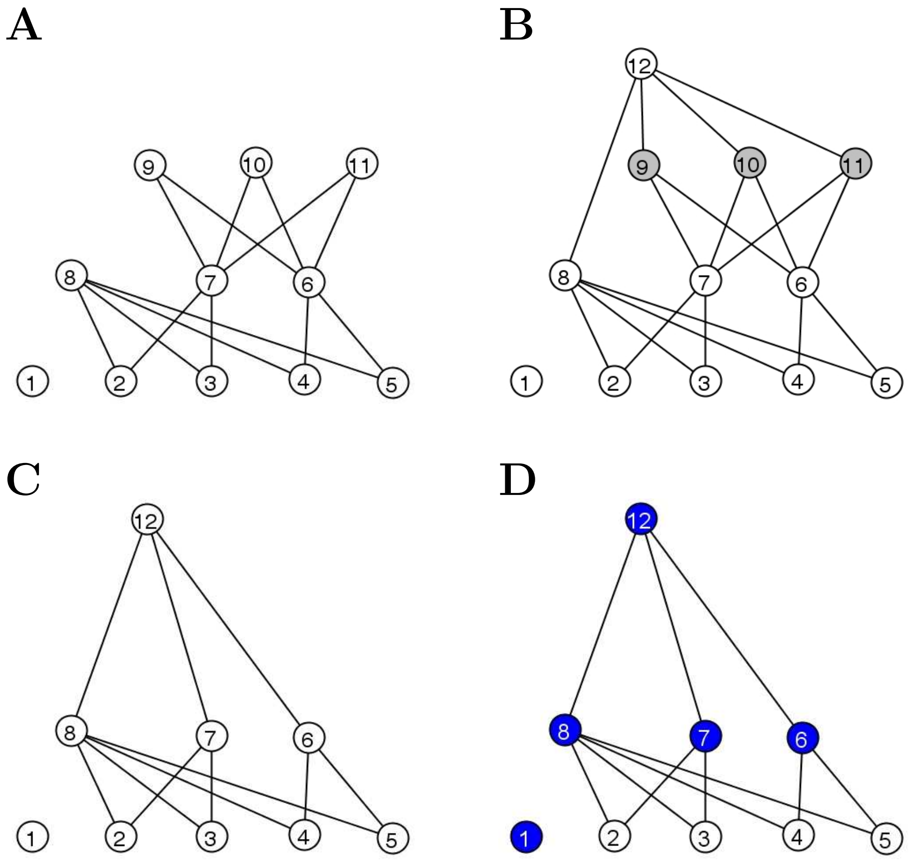

Figure S3.5: Examples of hierarchy involving partial state sequences from Fig.S3.3. In each case, node i corresponds to the partial state sequence P_i. In each case, if a link joins a partial state sequence P_x (top) to another P_y (bottom), P_x occurs in P_y and is conserved across a greater number of attractors. (A) Hierarchy between intersection sequences. (B) Hierarchy after the core component P_{12} is introduced. Grey nodes correspond to those sequences that are NOT partition sequences. Removing these grey nodes gives rise to the hierarchy in C. (D) Hierarchy between partition sequences. Blue nodes correspond to sequences with sub-dynamics that are distinct from those in sequences further up the hierarchy. These 5 distinct sub-dynamics are the subsystems. White nodes correspond to sequences that are just a combination of sequences further up the hierarchy, in D.

Table S3.2: Table showing the collections of subsystems that regulate each individual subsystem S_y (for the Boolean network model in Fig.S3.3 and subsystems in Fig.S3.4). Column 2: Each row shows a collection of subsystems ($\mathbb{S}_{group}$) that can trigger S_y

**Subsystem**	**Regulation set**
S_1	$\mathbb{S} = \{S_1\}$
S_2	$\mathbb{S} = \{S_2,S_5\}$
S_3	$\mathbb{S} = \{S_3,S_5\}$
S_4	$\mathbb{S} = \{S_4,S_5\}$
S_5	$\mathbb{S} = \{S_5\}$

S3.3 Example 3: Subsystems and their regulation

In order to show that the subsystem definitions are suitable for larger systems, we test it out on the Boolean network model shown in Fig.S3.6A and B (below). The primary objectives of this example is to (a) show that the new method gives sensible subsystems and (b) demonstrate the method for deciphering how each subsystem is regulated.

S3.3.1 Identifying subsystems

The model in Fig.S3.6A and B is relatively simple in that it is constructed from common network motifs, many of which are associated with genetic regulatory networks (e.g. Lee et al (2002), Shen-Orr et al (2002), Tyson et al (2003), Lahav et al (2004), Elowitz & Leibler (2000)). However, the global dynamics appear much more complicated, since it has 129 attractors.

This model has 20 subsystems (shown in Fig.S3.7), which can be found directly from the 129 attractor cycles and significantly simplify the description of the dynamics. In groups A, B, C, D, all of the subsystems are completely consistent with the network motifs. In group E, the subsystems are slightly more complex but still accurately capture key sub-dynamics in the system. n_{11} and n_{12} are part of a network motif called the toggle switch and appear together in 3 subsystems (S_{E1}, S_{E4} and S_{E5}), each one representing a different state the toggle switch can take. However, in all 3 subsystems, other nodes are involved because the states of those nodes are inseparable from the state of the toggle switch. Interestingly S_{E1}, S_{E4} and S_{E5} also capture the dynamics of the feed-forward loop involving n_{12}, n_{13} and n_{14}. In S_{E1} and S_{E4}, there is no permanent signal from n_{12}. In this case, n_{13} acts as a filter and appears in these subsystems, whilst n_{14} is left OFF and occurs in a separate subsystem on its own (S_{E3}). However, once n_{12} is permanently ON (S_{E5}), n_{13} and n_{14} both receive the signal and appear in the same subsystem.

A point to note here, is that subsystems (of size $q > 1$) are specifically associated with the type of updating scheme used in the default Boolean network model (i.e. deterministic and synchronous updates). For example, S_{C3}, S_{C4}, S_{C5} and S_{C6} might not occur within attractors from an equivalent model with asynchronous / stochastic updates. However, after identifying subsystems with a default Boolean network model, subsystems can be grouped together to isolate parts of the network worth studying in more detail. For example, because S_{C1}, S_{C2}, S_{C3}, S_{C4}, S_{C5} and S_{C6} are identified as subsystems and they involve the same set of nodes (n_5, n_6, n_7, n_8), they can be grouped together to isolate a part of the network that is possibly worth studying with more detailed mathematical models.

A

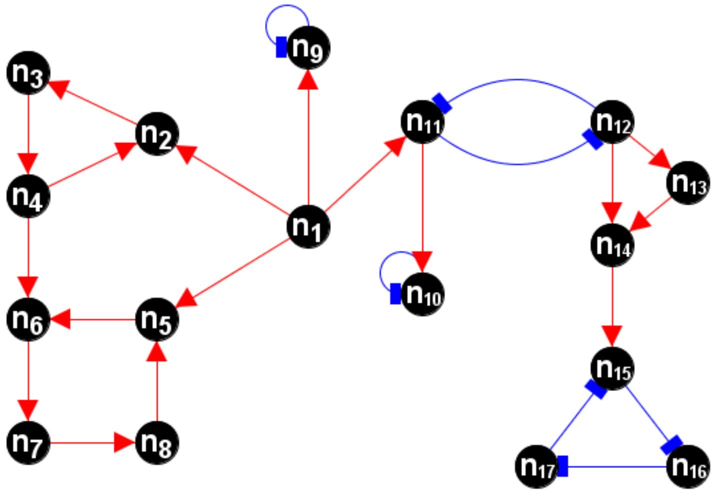

B

Node	Boolean function
n_1	$s_1(t+1) = 1$ IF $s_1(t) = 1$<br>$s_1(t+1) = 0$ otherwise
n_2	$s_2(t+1) = 1$ IF $s_1(t) = 1$ AND $s_4(t) = 1$<br>$s_2(t+1) = 0$ otherwise
n_3	$s_3(t+1) = 1$ IF $s_2(t) = 1$<br>$s_3(t+1) = 0$ otherwise
n_4	$s_4(t+1) = 1$ IF $s_3(t) = 1$<br>$s_4(t+1) = 0$ otherwise
n_5	$s_5(t+1) = 1$ IF $s_1(t) = 1$ AND $s_8(t) = 1$<br>$s_5(t+1) = 0$ otherwise
n_6	$s_6(t+1) = 1$ IF $s_4(t) = 1$ OR $s_5(t) = 1$<br>$s_6(t+1) = 0$ otherwise
n_7	$s_7(t+1) = 1$ IF $s_6(t) = 1$<br>$s_7(t+1) = 0$ otherwise
n_8	$s_8(t+1) = 1$ IF $s_7(t) = 1$<br>$s_8(t+1) = 0$ otherwise
n_9	$s_9(t+1) = 1$ IF $s_1(t) = 1$ AND $s_9(t) = 0$<br>$s_9(t+1) = 0$ otherwise

Node	Boolean function
n_{10}	$s_{10}(t+1) = 1$ IF $s_{11}(t) = 1$ AND $s_{10}(t) = 0$<br>$s_{10}(t+1) = 0$ otherwise
n_{11}	$s_{11}(t+1) = 1$ IF $s_1(t) = 1$ OR $s_{12}(t) = 0$<br>$s_{11}(t+1) = 0$ otherwise
n_{12}	$s_{12}(t+1) = 1$ IF $s_{11}(t) = 0$<br>$s_{12}(t+1) = 0$ otherwise
n_{13}	$s_{13}(t+1) = 1$ IF $s_{12}(t) = 1$<br>$s_{13}(t+1) = 0$ otherwise
n_{14}	$s_{14}(t+1) = 1$ IF $s_{12}(t) = 1$ AND $s_{13}(t) = 1$<br>$s_{14}(t+1) = 0$ otherwise
n_{15}	$s_{15}(t+1) = 1$ IF $s_{14}(t) = 1$ OR $s_{17}(t) = 0$<br>$s_{15}(t+1) = 0$ otherwise
n_{16}	$s_{16}(t+1) = 1$ IF $s_{15}(t) = 0$<br>$s_{16}(t+1) = 0$ otherwise
n_{17}	$s_{17}(t+1) = 1$ IF $s_{16}(t) = 0$<br>$s_{17}(t+1) = 0$ otherwise

Figure S3.6: Example Boolean network model made up from common network motifs. The subsystems for this model are shown in Fig.S3.7

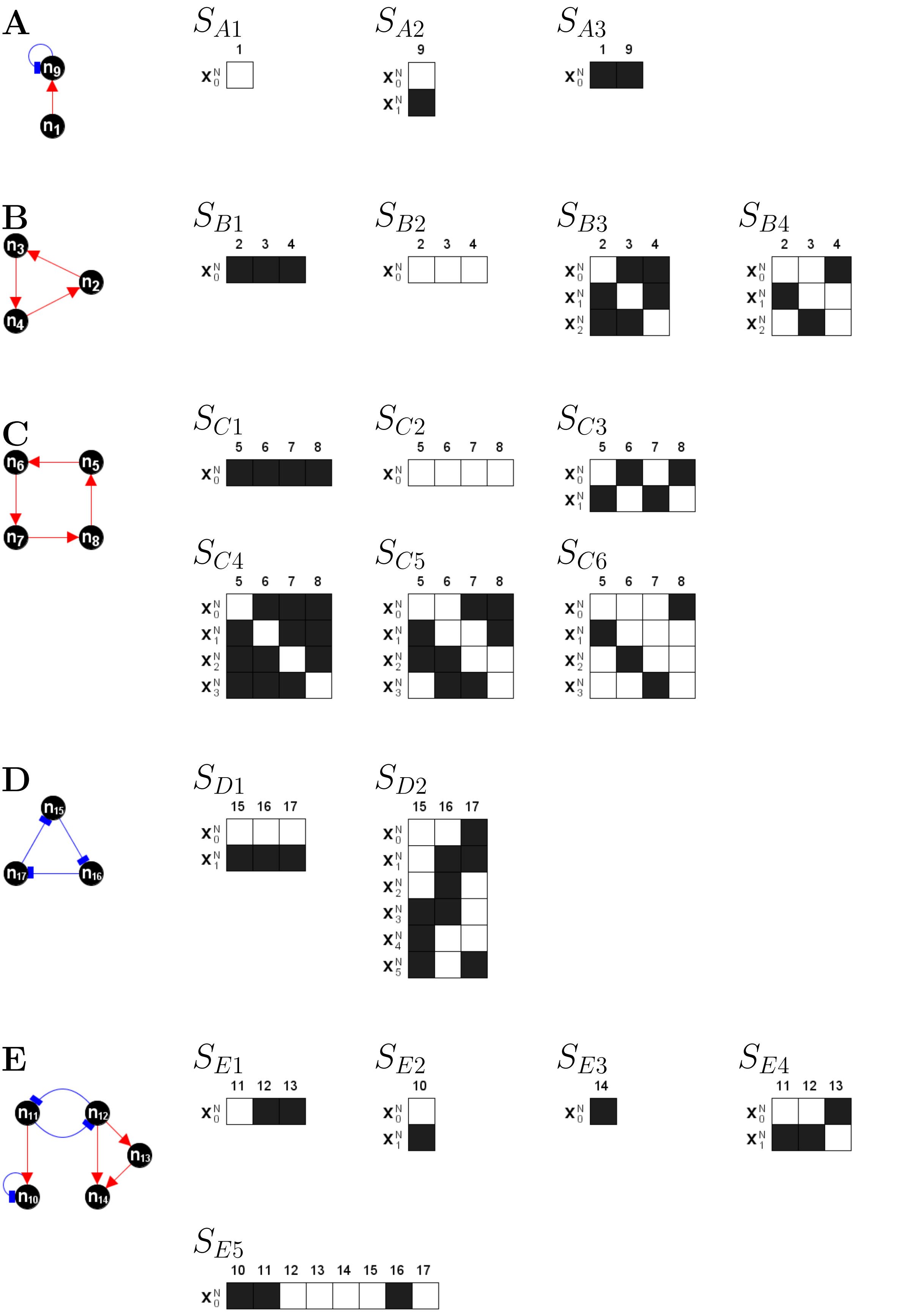

Figure S3.7: Diagrams showing subsystems for the Boolean network model in Fig.S3.6. Subsystems are grouped together into 5 groups A, B, C, D and E, depending on which network motifs they correspond to

S3.3.2 Regulation of subsystems

For each individual subsystem S_y (in this example), Table S3.3 gives a set of subsystem collections $\mathbb{S}_1$, ..., $\mathbb{S}_g$ that are responsible for regulating it. In each case, $\mathbb{S}_1$, ..., $\mathbb{S}_g$ is called the *regulation set* of S_y and satisfies the properties of Definition 7 (in the main text).

To demonstrate the methods employed, we discuss how the regulation set was determined for the subsystem S_{C2}. The basic idea is to look at every attractor A, containing S_{C2}, and look at which collections of subsystems $\mathbb{S}$ are responsible for *triggering* the occurrence of S_{C2} in A (i.e. which collections of subsystems $\mathbb{S}$ satisfy Definition S2.14 in *Supporting Text 2*).

Consider the subsystem S_{C2}

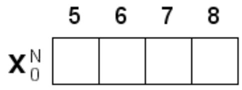

in the following attractor A

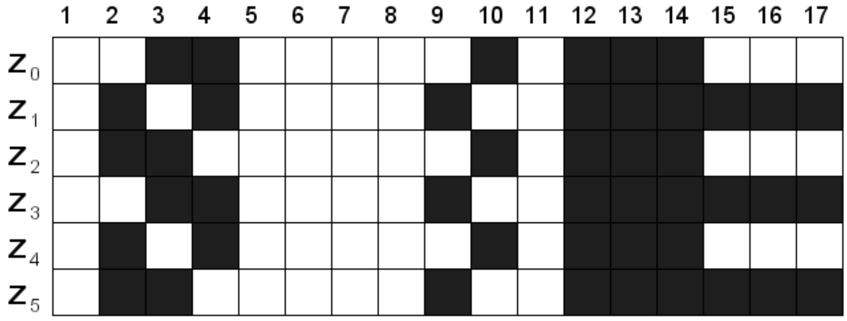

In Fig.S3.8,

A : We take the partial state $\mathbf{x}_0^N$ in the attractor state $\mathbf{z}_a = \mathbf{z}_0$

B : We take the partial state $\mathbf{x}_0^N$ in the attractor state $\mathbf{z}_a = \mathbf{z}_5$.

Then, in both cases, going *backwards* around the attractor and looking at the Boolean functions in Fig.S3.6B, it is possible to identify a sequence of partial states $\mathbf{z}_k^{P_k}$, ..., $\mathbf{z}_{k-t}^{P_{k-t}}$, ..., $\mathbf{z}_0^{P_0}$ for which

(a) $k = cp$, where c is the number of times we go backwards round the attractor and p is the number of attractor states

(b) $P_k = N$ and $\mathbf{z}_k^{P_k} = \mathbf{x}_0^N$

(c) For $i = 0, ..., k-1$ $(i = k-t)$, $\mathbf{z}_i^{P_i}$ ensures the occurrence of $\mathbf{z}_{i+1}^{P_{i+1}}$ in the following time step.

(d) For $i = 0, ..., k$ $(i = k-t)$, $\mathbf{z}_i^{P_i}$ is contained in the attractor state $\mathbf{z}_b \in A$ (where $b = a + i \pmod p = a - t \pmod p$, and a is the index of the initial attractor state)

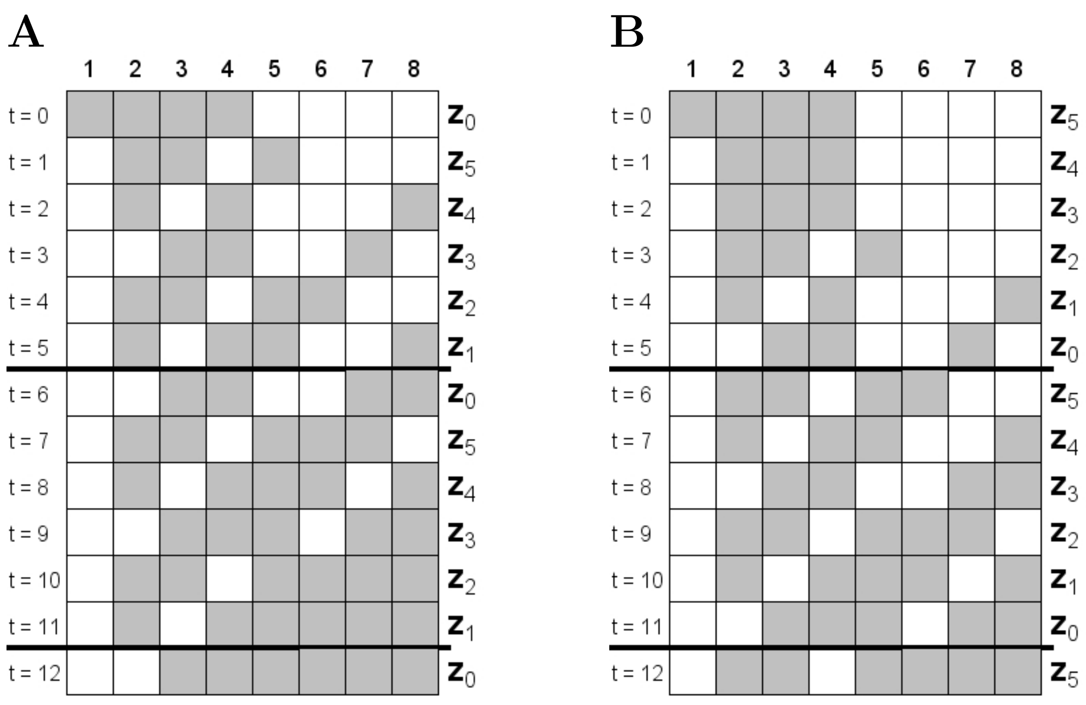

Figure S3.8: See text for details

Here, white implies a node has state 1 and grey implies the node is not involved in the partial state sequence (i.e. the state is not important). The bold lines correspond to progressions backwards through a single loop of the attractor (starting with the initial attractor state $\mathbf{z}_a$).

In the left hand figure (**A**), the occurrence of $\mathbf{x}_0^N$ in the attractor state $\mathbf{z}_0$ can be caused by the occurrence of the partial state

$$\mathbf{y}_0^{M_0} = \mathbf{z}_0^{P_0} = \{s_1 = 1, \, s_2 = 1\}$$

in $\mathbf{z}_0$ ($k = 12$ times steps previously). We say $\mathbf{y}_0^{M_0}$ is a k-predecessor of $\mathbf{x}_0^N$ in $\mathbf{z}_0$ (see Definition S2.9 in *Supporting Text 2*). Moreover,

$$\mathbf{y}_0^{M_0} = \{s_1 = 1, \, s_2 = 1\} \text{ is part of the subsystem collection } \mathbb{S} = \{S_{A1}, \, S_{B3}\}$$

In fact the occurrence of $S_{C2} = \{\mathbf{x}_0^N\}$ in $\mathbf{z}_0$, ..., $\mathbf{z}_5$ can be caused by the occurrence of partial states $\mathbf{y}_0^{M_0}$, ..., $\mathbf{y}_5^{M_5} \in \mathbb{S} = \{S_{A1}, \, S_{B3}\}$ in $\mathbf{z}_0$, ..., $\mathbf{z}_5$ (resp). The left hand figure (**A**) shows the case $a = 0$ (discussed above). The right hand figure (**B**), shows the case $a = 5$, with $\mathbf{y}_5^{M_5} = \{s_1 = 1, \, s_4 = 1\} \in \mathbb{S} = \{S_{A1}, \, S_{B3}\}$ (which occurs in $\mathbf{z}_5$). Other cases can be shown in a similar fashion.

Therefore, by following the model backwards in time, we can prove that the occurrence of $S_{C2} = \{\mathbf{x}_0^N\}$ in A can be explained by the combined occurrence of S_{A1} and S_{B3} at earlier points in time. Therefore, we say that the collection of subsystems $\mathbb{S} = \{S_{A1}, \, S_{B3}\}$ *triggers* S_{C2} in A.

Similarly, we can say that $\mathbb{S} = \{S_{A1}, S_{C2}\}$ *triggers* S_{C2} in A. This is because the occurrence of $S_{C2} = \{\mathbf{x}_0^N\}$ in $\mathbf{z}_0$, ..., $\mathbf{z}_5$ can be caused by the occurrence of partial states $\mathbf{y}_0^{M_0}$, ..., $\mathbf{y}_5^{M_5} \in \mathbb{S} = \{S_{A1}, S_{C2}\}$ in $\mathbf{z}_0$, ..., $\mathbf{z}_5$ (resp). The case $a = 0$ is shown below.

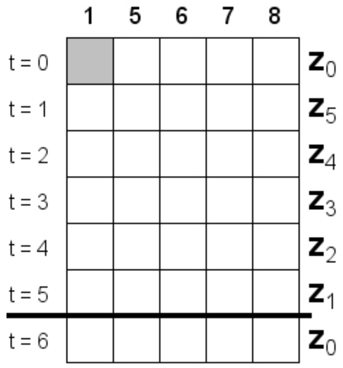

Different collections of subsystems are responsible for triggering S_{C2} in different attractors. Carrying out the above procedure, for every attractor that contains S_{C2}, we find that $\mathbb{S}_a = \{S_{A1}, S_{C2}\}$, $\mathbb{S}_b = \{S_{A1}, S_{B2}\}$, $\mathbb{S}_c = \{S_{A1}, S_{B3}\}$ and $\mathbb{S}_d = \{S_{A1}, S_{B4}\}$ are all capable of triggering S_{C2}. This gives us the regulation set in Table S3.3.

In *Supporting Text 2*, we describe an algorithm for finding every collection of subsystems $\mathbb{S}$ that *triggers* an individual subsystem S_y in an attractor A (Procedure S2.19 in Section S2.2.2 of *Supporting Text 2*). Carrying out this procedure for every attractor A, containing S_y, will give us the full regulation set.

The use of hierarchical links

Even without the Boolean functions, it is possible to identify relationships between subsystems. On a simple observational level, a subsystem S_x may be *hierarchically linked* to another subsystem S_y, because S_x only occurs in an attractor in conjunction with the 'higher order' S_y (Definition S2.21 in Section S2.3 of *Supporting Text 2*).

For this example, the hierarchical links between subsystems are given in Table S3.4. However, the direction of a hierarchical link does not necessarily correspond with the direction of the interaction in the underlying model. For example, returning to S_{C2};

S_{A1}, S_{B2}, S_{B3}, and S_{B4} are all involved in regulating S_{C2} but

(a) S_{C2} is hierarchically linked (directly) to S_{A1} (and additionally S_{A2}).

(b) S_{B2}, S_{B3}, and S_{B4} are all hierarchically linked (directly) to S_{C2}

First part(a). From Table S3.3, all 4 collections that trigger S_{C2}, require S_{A1} and so S_{C2} only occurs in an attractor also containing S_{A1}. Additionally, S_{A1} (on its own) directly triggers S_{A2}. Therefore, S_{C2} only occurs in attractors containing S_{A1} and S_{A2}.

Now part (b). S_{B2} can only occur alongside S_{A1} and, in this situation $Sgroup = \{S_{A1}, S_{B2}\}$ can trigger S_{C2}. Therefore, if S_{B2} occurs in an attractor, so must S_{C2}, implying S_{B2} is hierarchically linked to S_{C2}. The same is true for S_{B3} and S_{B4}.

Table S3.3: Table showing the collections of subsystems that regulate each individual subsystem S_y (for the Boolean network model in Fig.S3.6 and subsystems in Fig.S3.7). Column 2: Each row shows a collection of subsystems ($Sgroup$) that can directly trigger S_y

**Subsystem**	**Regulation set**
S_{A1}	$\mathbb{S} = \{S_{A1}\}$
S_{A2}	$\mathbb{S} = \{S_{A1}, S_{A2}\}$
S_{A3}	$\mathbb{S} = \{S_{A3}\}$
S_{B1}	$\mathbb{S} = \{S_{A3}\}$<br>$\mathbb{S} = \{S_{B1}\}$
S_{B2}	$\mathbb{S} = \{S_{A1}, S_{B2}\}$
S_{B3}	$\mathbb{S} = \{S_{A1}, S_{B3}\}$
S_{B4}	$\mathbb{S} = \{S_{A1}, S_{B4}\}$
S_{C1}	$\mathbb{S} = \{S_{A3}\}$<br>$\mathbb{S} = \{S_{B1}, S_{C1}\}$
S_{C2}	$\mathbb{S} = \{S_{A1}, S_{B2}\}$<br>$\mathbb{S} = \{S_{A1}, S_{B3}\}$<br>$\mathbb{S} = \{S_{A1}, S_{B4}\}$<br>$\mathbb{S} = \{S_{A1}, S_{C2}\}$
S_{C3}	$\mathbb{S} = \{S_{A1}, S_{B1}, S_{C3}\}$
S_{C4}	$\mathbb{S} = \{S_{A1}, S_{B1}, S_{C4}\}$
S_{C5}	$\mathbb{S} = \{S_{A1}, S_{B1}, S_{C5}\}$
S_{C6}	$\mathbb{S} = \{S_{A1}, S_{B1}, S_{C6}\}$
S_{D1}	$\mathbb{S} = \{S_{D1}, S_{E1}, S_{E3}\}$<br>$\mathbb{S} = \{S_{D1}, S_{E3}, S_{E4}\}$
S_{D2}	$\mathbb{S} = \{S_{D2}, S_{E1}, S_{E3}\}$<br>$\mathbb{S} = \{S_{D2}, S_{E3}, S_{E4}\}$
S_{E1}	$\mathbb{S} = \{S_{A1}\}$<br>$\mathbb{S} = \{S_{E1}\}$
S_{E2}	$\mathbb{S} = \{S_{E1}, S_{E2}\}$<br>$\mathbb{S} = \{S_{A3}, S_{E4}\}$
S_{E3}	$\mathbb{S} = \{S_{A1}\}$<br>$\mathbb{S} = \{S_{E1}\}$<br>$\mathbb{S} = \{S_{E4}\}$
S_{E4}	$\mathbb{S} = \{S_{A3}, S_{E4}\}$
S_{E5}	$\mathbb{S} = \{S_{A3}, S_{E5}\}$

Therefore, if we only have attractors (i.e. no mathematical model), the hierarchical links could potentially correspond to relationships between subsystem, that are worth studying in more detail. However, without details of a mathematical model, it is not possible to understand the nature of these relationships.

Table S3.4: Table showing hierarchical links between individual subsystems in Fig.S3.7 (for the Boolean network model in Fig.S3.6). For each subsystem, S_x, the final column shows all of the subsystems, S_y, that it is hierarchically linked to. From these links, the direct ones are shown in the second column.

**Subsystem**	**Direct Links**	**All Hierarchical links**
S_{A1}	S_{A2}, S_{E1}	S_{A2}, S_{E1}, S_{E2}, S_{E3}
S_{A2}	S_{A1}, S_{E1}	S_{A1}, S_{E1}, S_{E2}, S_{E3}
S_{A3}	S_{C1}	S_{B1}, S_{C1}
S_{B1}	–	–
S_{B2}	S_{C2}	S_{A1}, S_{A2}, S_{C2}, S_{E1}, S_{E2}, S_{E3}
S_{B3}	S_{C2}	S_{A1}, S_{A2}, S_{C2}, S_{E1}, S_{E2}, S_{E3}
S_{B4}	S_{C2}	S_{A1}, S_{A2}, S_{C2}, S_{E1}, S_{E2}, S_{E3}
S_{C1}	S_{B1}	S_{B1}
S_{C2}	S_{A1}, S_{A2}	S_{A1}, S_{A2}, S_{E1}, S_{E2}, S_{E3}
S_{C3}	S_{A1}, S_{A2}, S_{B1}	S_{A1}, S_{A2}, S_{B1}, S_{E1}, S_{E2}, S_{E3}
S_{C4}	S_{A1}, S_{A2}, S_{B1}	S_{A1}, S_{A2}, S_{B1}, S_{E1}, S_{E2}, S_{E3}
S_{C5}	S_{A1}, S_{A2}, S_{B1}	S_{A1}, S_{A2}, S_{B1}, S_{E1}, S_{E2}, S_{E3}
S_{C6}	S_{A1}, S_{A2}, S_{B1}	S_{A1}, S_{A2}, S_{B1}, S_{E1}, S_{E2}, S_{E3}
S_{D1}	S_{E2}, S_{E3}	S_{E2}, S_{E3}
S_{D2}	S_{E2}, S_{E3}	S_{E2}, S_{E3}
S_{E1}	S_{E2}, S_{E3}	S_{E2}, S_{E3}
S_{E2}	S_{E3}	S_{E3}
S_{E3}	S_{E2}	S_{E2}
S_{E4}	S_{A3}, S_{E2}, S_{E3}	S_{A3}, S_{B1}, S_{C1}, S_{E2}, S_{E3}
S_{E5}	S_{A3}	S_{A3}, S_{B1}, S_{C1}

References

Chen KC, Calzone L, Csikasz-Nagy A, Cross FR, Novak B, and Tyson JJ (2004) *Mol. Biol. Cell.* **15** 3841–3862.

Elowitz MB and Leibler S (2000) *Nature.* **403** 335–338.

Lahav G, Rosenfeld N, Signal A, Geva-Zatorsky N, Levine AJ, Elowitz MB and Alon U (2004) *Nat. Genet.* **36** 147–150.

Lee TI, Rinaldi NJ, Robert F, Odom DT, Bar-Joseph Z, Gerber GK, Hannett NM, Harbison CT, Thompson CM, Simon I, Zeitlinger J, Jennings EG, Murray HL, Gordon DB, Ren B, Wyrick JJ, Tagne J-B, Volkert TL, Fraenkel E, Gifford DK and Young RA (2002) *Science.* **298** 799–804.

Shen-Orr SS, Milo R, Mangan S and Alon U (2002) *Nat. Genet.* **31** 64–68.

Tyson JJ, Chen KC and Novak B (2003) *Curr. Opin. Cell. Biol.* **15** 221–231.
