# Supplementary material for: Identifying dynamical modules from genetic regulatory systems: applications to the segment polarity network
Source: BMC Bioinformatics. 2007 Oct 25;8:413. doi: 10.1186/1471-2105-8-413 (PMC2233651; doi:10.1186/1471-2105-8-413)
Supplement: Additional file 4 — Extra information associated with the model/analysis of the segment polarity network. This is given as a pdf file and is referred to in the main text. [file 1471-2105-8-413-S4.pdf]

# Supporting Text 4:
# Supplementary data for *Drosophila* segment polarity network

## S4.1 Boolean network model

In the main text, we consider an existing Boolean network model of the segment polarity network (from Albert & Othmer (2003) and Chaves *et al* (2005)).

This model corresponds to a 1 dimensional array of cells, with each individual cell having the same 13 Boolean functions, shown in Table S4.1 (and justified in Albert & Othmer (2003)). Some interactions, such as those involving *wingless* and *hedgehog* proteins (WG and HH respectively), are inter-cellular so that the protein in one cell can trigger a response in the neighbouring cell. Because of a previous developmental stage, *sloppy paired* protein (SLP) is only present in the posterior of each parasegment in this case (cells 3 and 4). Because the parasegments start off 4 cells wide and the expression pattern associated with the main proteins are assumed to repeat every 4 cells, previous models primarily considered a 4 cell ring of cells where inter-cellular interactions can occur between cells 1 and 4. It is this 4 cell version of the model that is considered here.

There are 10 attractors for this model that are all fixed points (i.e. attractors only have a single state). The 10 fixed points (shown in Fig.S4.1) were found analytically in Albert & Othmer (2003) and confirmed using the algorithm in Irons (2006). The absence of any limit cycles was also confirmed using the algorithm in Irons (2006).

The wild type expression pattern is represented by the attractor $A_1$. This is characterised by WG and EN / HH expression in 1 cell wide stripes either side of the parasegment boundary (in cell 4 and 1 resp). As discussed in Albert & Othmer (2003), the fixed points $A_2$ and $A_3$ correspond to expression patterns observed under experimental conditions, where certain genes are over-expressed or silenced. Temporarily inducing *hh* across the embryo, following heat shock, leads to the broader WG, EN and HH stripes seen in $A_2$. $A_3$ can occur whenever any of *en*, *wg* or *hh* is mutated. Significantly delaying *wg* initiation or disrupting inter-cellular signalling can also have the same result. The model has also been shown to be consistent with other mutations, caused by knocking out genes. However, these ′knock-outs′ change the underlying model and lead to novel fixed point attractor cycles. These attractor cycles are not considered here but provide further evidence that the model is suitable.

As an aside, all 10 attractors are fixed points and so they will still be attractors for a Boolean network model with asynchronous updates. Therefore, since identification of subsystems only depends on attractors, there is no real advantage in considering asynchronous updating schemes here. However, asynchronous updating schemes would alter the basins of attraction for the 10 attractors, and so may be advantageous when studying other aspects of the model.

## S4.2 Occurrence of subsystems in attractors

This model has 19 subsystems, shown in Figure 4 and Table 1 of the main text. For each of these subsystems, the set of attractors containing the subsystems (*occurrence set*) is shown in Table S4.2. These occurrence set can then be used to identify the *hierarchical links* in Table S4.3 (satisfying Definition S2.21 in Section S2.3 of *Supporting Text 2*)

Table S4.1: Boolean functions for each node in cell $i$. The subscript for a node corresponds to the cell index. For example if $i = 2$, then $HH_{i-1}$ corresponds to the protein HH in cell $i - 1 = 1$. Each row (containing node states) represents a set of conditions (at time $t$) that lead to the node taking state 1 at time $t + 1$. If none of these conditions are met, the node takes state 0 at time $t + 1$

| **Node** | **State at $t+1$** | **Condition** |
|---|---|---|
| $SLP_i$ | 1 | $i \pmod 4 = 0$ OR $i \pmod 4 = 3$ |
| | 0 | Otherwise |
| $wg_i$ | 1 | $CIR_i = 0$ AND $CIA_i = 1$ AND $SLP_i = 1$ |
| | 1 | $CIR_i = 0$ AND $CIA_i = 1$ AND $wg_i = 1$ |
| | 1 | $CIR_i = 0$ AND $SLP_i = 1$ AND $wg_i = 1$ |
| | 0 | Otherwise |
| $WG_i$ | 1 | $wg_i = 1$ |
| | 0 | Otherwise |
| $en_i$ | 1 | $SLP_i = 0$ AND ($WG_{i-1} = 1$ OR $WG_{i+1} = 1$) |
| | 0 | Otherwise |
| $EN_i$ | 1 | $en_i = 1$ |
| | 0 | Otherwise |
| $hh_i$ | 1 | $CIR_i = 0$ AND $EN_i = 1$ |
| | 0 | Otherwise |
| $HH_i$ | 1 | $hh_i = 1$ |
| | 0 | Otherwise |
| $ptc_i$ | 1 | $CIR_i = 0$ AND $EN_i = 0$ AND $CIA_i = 1$ |
| | 0 | Otherwise |
| $PTC_i$ | 1 | $ptc_i = 1$ |
| | 1 | $HH_{i-1} = 0$ AND $HH_{i+1} = 0$ AND $PTC_i = 1$ |
| | 0 | Otherwise |
| $ci_i$ | 1 | $EN_i = 0$ |
| | 0 | Otherwise |
| $CI_i$ | 1 | $ci_i = 1$ |
| | 0 | Otherwise |
| $CIA_i$ | 1 | $CI_i = 1$ AND $PTC_i = 0$ |
| | 1 | $CI_i = 1$ AND ($HH_{i-1} = 1$ OR $HH_{i+1} = 1$) |
| | 1 | $CI_i = 1$ AND ($hh_{i-1} = 1$ OR $hh_{i+1} = 1$) |
| | 0 | Otherwise |
| $CIR_i$ | 1 | $CI_i = 1$ AND $PTC_i = 1$ AND $HH_{i-1} = 0$ AND $HH_{i+1} = 0$ AND $hh_{i-1} = 0$ AND $hh_{i+1} = 0$ |
| | 0 | Otherwise |

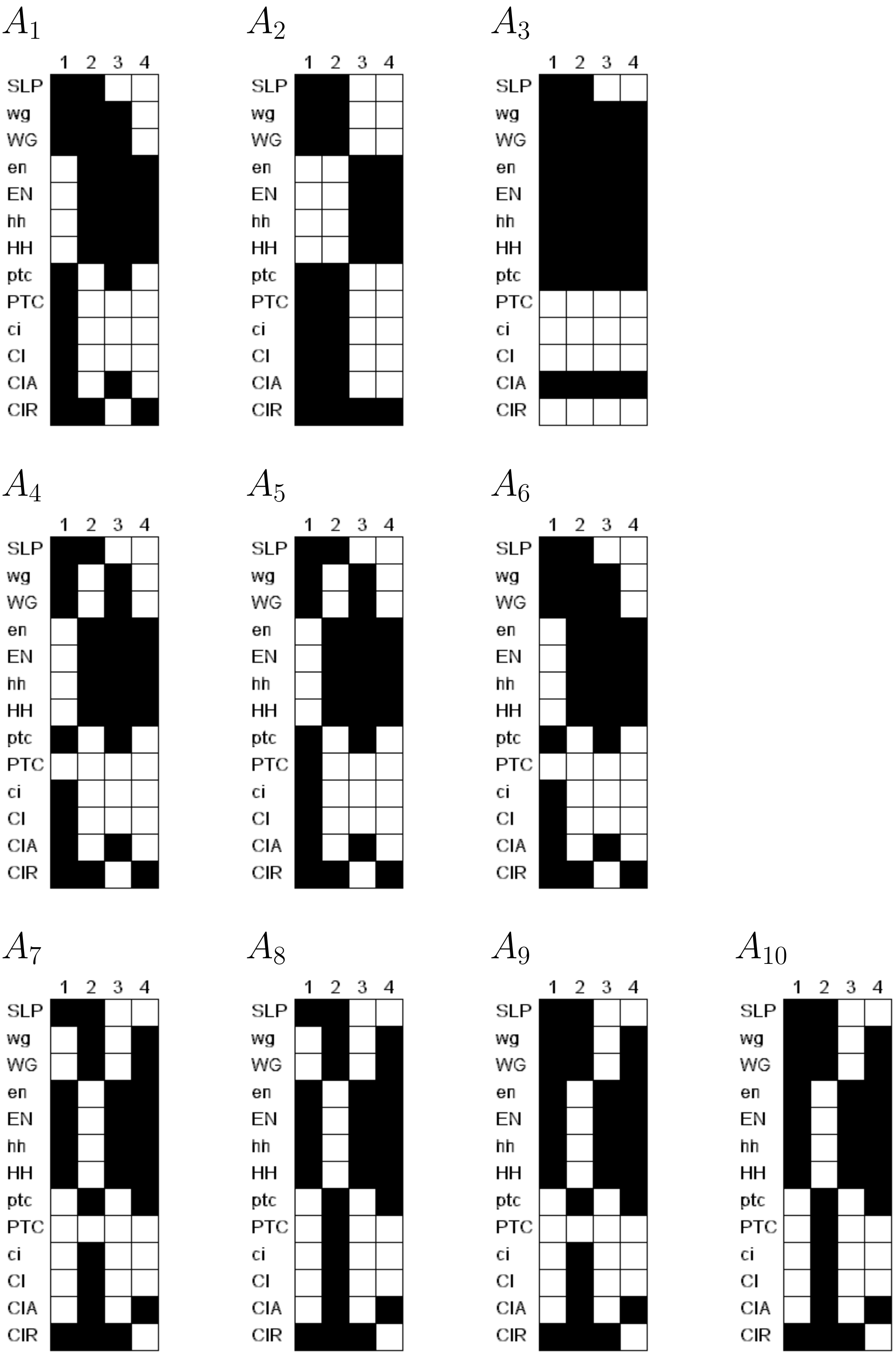


Figure S4.1: Visual representation of the 10 attractors, for the Boolean network model of the *Drosophila* segment polarity network. Each attractors is a fixed point (i.e. a single state) for $13 \times 4 = 52$ nodes corresponding to 13 genes / proteins in 4 cells (1 to 4).

Table S4.2: Table showing the occurrence sets for each of the 19 subsystems (in Fig.4 and Table 1 of the main text). For each subsystem $S_i$, its occurrence set $\mathbb{C}_i$ is the set of attractors that contain $S_i$

| **Subsystem** | **Occurrence set** |
|---|---|
| $S_A$ | $\mathbf{C}_A = \{A_1, A_2, A_3, A_4, A_5, A_6, A_7, A_8, A_9, A_{10}\}$ |
| $S_{B1}$ | $\mathbf{C}_{B1} = \{A_1, A_2, A_4, A_5, A_6\}$ |
| $S_{B2}$ | $\mathbf{C}_{B2} = \{A_3, A_7, A_8, A_9, A_{10}\}$ |
| $S_{C1}$ | $\mathbf{C}_{C1} = \{A_2, A_7, A_8, A_9, A_10\}$ |
| $S_{C2}$ | $\mathbf{C}_{C2} = \{A_1, A_3, A_4, A_5, A_6\}$ |
| $S_{D1}$ | $\mathbf{C}_{D1} = \{A_7, A_8\}$ |
| $S_{D2}$ | $\mathbf{C}_{D2} = \{A_1, A_2, A_3, A_4, A_5, A_6, A_9, A_{10}\}$ |
| $S_{E1}$ | $\mathbf{C}_{E1} = \{A_4, A_5\}$ |
| $S_{E2}$ | $\mathbf{C}_{E2} = \{A_1, A_2, A_3, A_6, A_7, A_8, A_9, A_{10}\}$ |
| $S_{F1}$ | $\mathbf{C}_{F1} = \{A_3, A_4, A_6, A_7, A_8, A_9, A_{10}\}$ |
| $S_{F2}$ | $\mathbf{C}_{F2} = \{A_1, A_2, A_5\}$ |
| $S_{G1}$ | $\mathbf{C}_{G1} = \{A_1, A_3, A_4, A_5, A_6, A_7, A_9\}$ |
| $S_{G2}$ | $\mathbf{C}_{G2} = \{A_2, A_8, A_{10}\}$ |
| $S_{H1}$ | $\mathbf{C}_{H1} = \{A_7, A_8, A_9, A_{10}\}$ |
| $S_{H2}$ | $\mathbf{C}_{H2} = \{A_1, A_2, A_3, A_4, A_5, A_6\}$ |
| $S_{I1}$ | $\mathbf{C}_{I1} = \{A_1, A_4, A_5, A_6\}$ |
| $S_{I2}$ | $\mathbf{C}_{I2} = \{A_2, A_3, A_7, A_8, A_9, A_{10}\}$ |
| $S_{J1}$ | $\mathbf{C}_{J1} = \{A_3\}$ |
| $S_{J2}$ | $\mathbf{C}_{J2} = \{A_1, A_2, A_4, A_5, A_6, A_7, A_8, A_9, A_{10}\}$ |

Table S4.3: Table showing hierarchical links between individual subsystems. For each subsystem, $S_x$, the final column shows all of the subsystems, $S_y$, that it is hierarchically linked to. From these links, the direct ones are shown in the second column.

| **Subsystem** | **Direct Links** | **All Hierarchical links** |
|---|---|---|
| $S_A$ | – | – |
| $S_{B2}$ | $S_{F1}$, $S_{I2}$ | $S_A$, $S_{E2}$, $S_{F1}$, $S_{I2}$ |
| $S_{B1}$ | $S_{H2}$, $S_{J2}$ | $S_A$, $S_{D2}$, $S_{H2}$, $S_{J2}$ |
| $S_{C2}$ | $S_{G1}$, $S_{H2}$ | $S_A$, $S_{D2}$, $S_{G1}$, $S_{H2}$ |
| $S_{C1}$ | $S_{I2}$, $S_{J2}$ | $S_A$, $S_{E2}$, $S_{I2}$, $S_{J2}$ |
| $S_{D2}$ | $S_A$ | $S_A$ |
| $S_{D1}$ | $S_{H1}$ | $S_A$, $S_{B2}$, $S_{C1}$, $S_{E2}$, $S_{F1}$, $S_{H1}$, $S_{I2}$, $S_{J2}$ |
| $S_{E2}$ | $S_A$ | $S_A$ |
| $S_{E1}$ | $S_{I1}$ | $S_A$, $S_{B1}$, $S_{C2}$, $S_{D2}$, $S_{G1}$, $S_{H2}$, $S_{I1}$, $S_{J2}$ |
| $S_{F2}$ | $S_{B1}$ | $S_A$, $S_{B1}$, $S_{D2}$, $S_{H2}$, $S_{J2}$ |
| $S_{F1}$ | $S_A$ | $S_A$ |
| $S_{G2}$ | $S_{C1}$ | $S_A$, $S_{C1}$, $S_{E2}$, $S_{I2}$, $S_{J2}$ |
| $S_{G1}$ | $S_A$ | $S_A$ |
| $S_{H2}$ | $S_{D2}$ | $S_A$, $S_{D2}$ |
| $S_{H1}$ | $S_{B2}$, $S_{C1}$ | $S_A$, $S_{B2}$, $S_{C1}$, $S_{E2}$, $S_{F1}$, $S_{I2}$, $S_{J2}$ |
| $S_{I2}$ | $S_{E2}$ | $S_A$, $S_{E2}$ |
| $S_{I1}$ | $S_{B1}$, $S_{C2}$ | $S_A$, $S_{B1}$, $S_{C2}$, $S_{D2}$, $S_{G1}$, $S_{H2}$, $S_{J2}$ |
| $S_{J2}$ | $S_A$ | $S_A$ |
| $S_{J1}$ | $S_{B2}$, $S_{C2}$ | $S_A$, $S_{B2}$, $S_{C2}$, $S_{D2}$, $S_{E2}$, $S_{F1}$, $S_{G1}$, $S_{H2}$, $S_{I2}$ |

## S4.3 Using subsystems to understand attractors

As can be seen in Figure 5 of the main text, it is possible to use 13 subsystems to provide a hierarchical breakdown of the 10 attractors. Here, we demonstrate the main regulatory mechanisms involving these subsystems. This then allows us to understand this breakdown.

In Figure 5 (of the main text), the attractors are split up into 4 main groups depending on the occurrence of $S_{B1}$, $S_{B2}$, $S_{C1}$ and $S_{C2}$. We first analyse how the 4 subsystem collections $\mathbb{S}_1 = \{S_{B1}, S_{C1}\}$, $\mathbb{S}_2 = \{S_{B2}, S_{C2}\}$, $\mathbb{S}_3 = \{S_{B1}, S_{C2}\}$ and $\mathbb{S}_4 = \{S_{B2}, S_{C1}\}$ are regulated. Table S4.4 and Table S4.5 then summarises these findings

The following analysis uses the results from Table 2 of the main text, which describes how each subsystem is regulated. It must be noted here that, if a subsystem collection $\mathbb{S}$ can trigger an individual subsystem $S_y$, then so can any collection $\mathbb{S}' \supset \mathbb{S}$ (when it occurs in the same attractor)

**Case :** $\mathbb{S}_1 = \{S_{B1}, S_{C1}\}$

From Table 2 of the main text, it is evident that the subsystem collection $\mathbb{S}_a = \{S_A, S_{B1}, S_{C1}\}$ will be sufficient to regulate $S_A$, $S_{B1}$ and $S_{C1}$. Therefore $S_A$, $S_{B1}$ and $S_{C1}$ form a regulatory unit whereby, once they are fixed in place, they can maintain their own occurrence (in an attractor).

Moreover, as can be seen from Table 2 of the main text, $\mathbb{S}_a = \{S_A, S_{B1}, S_{C1}\}$ is capable of regulating $S_{D2}$, $S_{E2}$, $S_{F2}$, $S_{G2}$, $S_{H2}$, $S_{I2}$ and $S_{J2}$. Therefore, $\mathbb{S}_a = \{S_A, S_{B1}, S_{C1}\}$ is sufficient to control the remainder of the network. In this case, we get attractor $A_2$.

**Case :** $\mathbb{S}_2 = \{S_{B2}, S_{C2}\}$

Both $\mathbb{S}_{b_1} = \{S_A, S_{B2}, S_{C2}, S_{F1}, S_{G1}\}$ and $\mathbb{S}_{b_2} = \{S_A, S_{B2}, S_{C2}, S_{D2}, S_{E2}\}$ form regulatory units. i.e. once they are fixed in place, they can maintain their own occurrence (in an attractor).

$\mathbb{S}_{b_1} = \{S_A, S_{B2}, S_{C2}, S_{F1}, S_{G1}\}$ is capable of regulating $S_{D2}$, $S_{E2}$, $S_{H2}$, $S_{I2}$ and $S_{J1}$. Therefore, $\mathbb{S}_{b_1} = \{S_A, S_{B2}, S_{C2}, S_{F1}, S_{G1}\}$ is sufficient to control the remainder of the network. In this case, we get attractor $A_3$.

If $\mathbb{S}_{b_2}$ is fixed in place, the system is forced to adopt subsystems $S_{F1}$, $S_{G1}$ and so we return to $\mathbb{S}_{b_1}$. This is because $S_{B1}$, $S_{D1}$ and $S_{E1}$ can't occur (since it is impossible to have both $S_B$'s, $S_D$'s or $S_E$'s in the same attractor). Therefore $S_{F2}$ / $S_{G2}$ would require $S_{H2}$ / $S_{I2}$, which would require $S_{F1}$ / $S_{G1}$ (but it is impossible to have both $S_F$'s or both $S_G$'s in the same attractor).

**Case :** $\mathbb{S}_3 = \{S_{B1}, S_{C2}\}$

$\mathbb{S}_c = \{S_A, S_{B1}, S_{C2}, S_{D2}\}$ form a regulatory unit. i.e. once they are fixed in place, they can maintain their own occurrence (in an attractor).

$\mathbb{S}_c = \{S_A, S_{B1}, S_{C2}, S_{D2}\}$ is then capable of regulating $S_{G1}$, $S_{H2}$, $S_{I1}$ and $S_{J2}$. Therefore, $\mathbb{S}_c = \{S_A, S_{B1}, S_{C2}, S_{D2}\}$ is sufficient to control the remainder of the network, except the nodes associated with $S_{E1}$, $S_{E2}$, $S_{F1}$, $S_{F2}$. Extra regulatory events specify these 4 subsystems to form the attractors $A_1$, $A_4$, $A_5$ and $A_6$.

**Case :** $\mathbb{S}_4 = \{S_{B2},\, S_{C1}\}$

$\mathbb{S}_d = \{S_A,\, S_{B2},\, S_{C1},\, S_{E2}\}$ form a regulatory unit. i.e. once they are fixed in place, they can maintain their own occurrence (in an attractor).

$\mathbb{S}_d = \{S_A,\, S_{B2},\, S_{C1},\, S_{E2}\}$ is then capable of regulating $S_{F1}$, $S_{H1}$, $S_{I2}$ and $S_{J2}$. Therefore, $\mathbb{S}_d = \{S_A,\, S_{B2},\, S_{C1},\, S_{E2}\}$ is sufficient to control the remainder of the network, except the nodes associated with $S_{D1}$, $S_{D2}$, $S_{G1}$, $S_{G2}$. Extra regulatory events specify these 4 subsystems to form the attractors $A_7$, $A_8$, $A_9$ and $A_{10}$.

Table S4.4: For the 4 main sets of attractors, the subsystems in the second column are those responsible for distinguishing them from the remaining sets. The last column shows the subsystems necessary to regulate and control these key identifiers

| **Attractor cycles** | **Key (group) identifiers** | **Key regulators** |
|---|---|---|
| $\mathbf{C}_a = \{A_2\}$ | $\{S_{B1},\, S_{C1}\}$ | $\mathbb{S}_a = \{S_A,\, S_{B1},\, S_{C1}\}$ |
| $\mathbf{C}_b = \{A_3\}$ | $\{S_{B2},\, S_{C2}\}$ | $\mathbb{S}_b = \{S_A,\, S_{B2},\, S_{C2},\, S_{F1},\, S_{G1}\}$ |
| $\mathbf{C}_c = \{A_1,\, A_4,\, A_5,\, A_6\}$ | $\{S_{B1},\, S_{C2}\}$ | $\mathbb{S}_c = \{S_A,\, S_{B1},\, S_{C2},\, S_{D2}\}$ |
| $\mathbf{C}_d = \{A_7,\, A_8,\, A_9,\, A_{10}\}$ | $\{S_{B2},\, S_{C1}\}$ | $\mathbb{S}_d = \{S_A,\, S_{B2},\, S_{C1},\, S_{E2}\}$ |

Table S4.5: Subsystems that distinguish each attractor from its group in Table S4.4

| **Attractor cycle** | **Key (individual) identifiers** |
|---|---|
| $A_1$ | $\mathbb{S} = \{S_{E2},\, S_{F2}\}$ |
| $A_2$ | n/a |
| $A_3$ | n/a |
| $A_4$ | $\mathbb{S} = \{S_{E1},\, S_{F1}\}$ |
| $A_5$ | $\mathbb{S} = \{S_{E1},\, S_{F2}\}$ |
| $A_6$ | $\mathbb{S} = \{S_{E2},\, S_{F1}\}$ |
| $A_7$ | $\mathbb{S} = \{S_{D1},\, S_{G1}\}$ |
| $A_8$ | $\mathbb{S} = \{S_{D1},\, S_{G2}\}$ |
| $A_9$ | $\mathbb{S} = \{S_{D2},\, S_{G1}\}$ |
| $A_{10}$ | $\mathbb{S} = \{S_{D2},\, S_{G2}\}$ |

# References


Albert R and Othmer HG (2003) *J. Theor. Biol.* **223** 1-18.

Chaves M, Albert R, and Sontag ED (2005) *J. Theor. Biol.* **235** 431-449.

Irons DJ (2006) *Physica D* **217** 7–21
